# Genome-wide significant regions in 43 Utah high-risk families implicate multiple genes involved in risk for completed suicide

**DOI:** 10.1038/s41380-018-0282-3

**Published:** 2018-10-23

**Authors:** Hilary Coon, Todd M. Darlington, Emily DiBlasi, W. Brandon Callor, Elliott Ferris, Alison Fraser, Zhe Yu, Nancy William, Sujan C. Das, Sheila E. Crowell, Danli Chen, John S. Anderson, Michael Klein, Leslie Jerominski, Dale Cannon, Andrey Shabalin, Anna Docherty, Megan Williams, Ken R. Smith, Brooks Keeshin, Amanda V. Bakian, Erik Christensen, Qingqin S. Li, Nicola J. Camp, Douglas Gray

**Affiliations:** 1 Department of Psychiatry, University of Utah School of Medicine, Salt Lake City, UT USA; 2 Utah State Office of the Medical Examiner, Utah Department of Health, Salt Lake City, UT USA; 3 Department of Neurobiology & Anatomy, University of Utah School of Medicine, Salt Lake City, UT USA; 4 Pedigree & Population Resource, Huntsman Cancer Institute, University of Utah, Salt Lake City, UT USA; 5 Department of Psychology, University of Utah, Salt Lake City, UT USA; 6 Health Sciences Center Core Research Facility, University of Utah, Salt Lake City, UT USA; 7 Department of Pediatrics, Intermountain Healthcare, Salt Lake City, UT USA; 8 Research Information Technology, Janssen Research & Development LLC, Pennington, NJ USA; 9 Department of Internal Medicine, University of Utah School of Medicine, Salt Lake City, UT USA

**Keywords:** Genetics, Depression

## Abstract

Suicide is the 10th leading cause of death in the United States. Although environment has undeniable impact, evidence suggests that genetic factors play a significant role in completed suicide. We linked a resource of ~ 4500 DNA samples from completed suicides obtained from the Utah Medical Examiner to genealogical records and medical records data available on over eight million individuals. This linking has resulted in the identification of high-risk extended families (7–9 generations) with significant familial risk of completed suicide. Familial aggregation across distant relatives minimizes effects of shared environment, provides more genetically homogeneous risk groups, and magnifies genetic risks through familial repetition. We analyzed Illumina PsychArray genotypes from suicide cases in 43 high-risk families, identifying 30 distinct shared genomic segments with genome-wide evidence (*p* = 2.02E-07–1.30E-18) of segregation with completed suicide. The 207 genes implicated by the shared regions provide a focused set of genes for further study; 18 have been previously associated with suicide risk. Although PsychArray variants do not represent exhaustive variation within the 207 genes, we investigated these for specific segregation within the high-risk families, and for association of variants with predicted functional impact in ~ 1300 additional Utah suicides unrelated to the discovery families. None of the limited PsychArray variants explained the high-risk family segregation; sequencing of these regions will be needed to discover segregating risk variants, which may be rarer or regulatory. However, additional association tests yielded four significant PsychArray variants (*SP110*, rs181058279; *AGBL2*, rs76215382; *SUCLA2*, rs121908538; *APH1B*, rs745918508), raising the likelihood that these genes confer risk of completed suicide.

## Introduction

Suicide is the 10th leading cause of death in the United States; over 44,000 individuals die by suicide in the United States every year [1]. Although environmental variables have undeniable impact, evidence suggests that genetic factors play a role in completed suicide, with heritability of close to 50% [2, 3]. Recent growth in the number of suicide genetic studies has resulted in promising findings from candidate gene and genome-wide association studies [4], though many remain to be replicated. Replication is hampered by sample differences across studies, including differences in demographics and primary diagnoses of study samples, as many studies of suicide risk have been conducted within cases ascertained for specific psychiatric disorders [4]. In addition, most studies of suicide have focused on suicidal ideation and behaviors; these phenotypes are much more common than completed suicide, allowing for ascertainment of sufficiently powered samples, but suicidal behaviors can be difficult to quantify, and represent individuals with a range of risk for later suicide. In addition, evidence suggests important differences in the etiology of suicidal behaviors versus the less ambiguous but much rarer outcome of completed suicide [5].

We implemented a unique study design to investigate genetic risk for suicide through the collection of DNA samples on > 4500 consecutive individuals who died by suicide in the state of Utah, providing an unparalleled population-based genetic resource. This sample results from a long-term collaboration with the Utah State Office of the Medical Examiner. The records from these cases have been linked to the Utah Population Database (UPDB, https://healthcare.utah.edu/huntsmancancerinstitute/research/updb), a comprehensive database including multi-generational genealogies, as well as death certificates, demographic data, and current medical information on over eight million individuals. Through this linking, we have identified very large families (7–9 generations) with significantly elevated suicide risk. Familial aggregation across distant relatives in these families minimizes the impact of shared environment on risk. High-risk families also provide more genetically homogeneous risk groups, increasing statistical power to detect familial variants associated with disease risk. The Utah extended family study design has already shown success in the study of other complex genetic diseases of extended families of similar size (e.g., colon cancer [6], breast cancer [7], and cardiac arrhythmia [8]).

This study reflects an analysis of 43 very large Utah families at significantly elevated risk for completed suicide.

The focus on completed suicide, statistically concentrated in these high-risk families, optimizes power to reveal regions of the genome likely to contain risk variants. Our design investigates genetic risk using the suicide cases in the extended families regardless of co-occurring psychopathology, and continues with follow-up studies from our population-wide ascertainment of all suicide deaths in Utah, again without regard to co-occurring psychopathology. We recognize that psychiatric diagnoses are critically important in suicide risk [9, 10]; it is likely that findings from our study are related to these associated risks. However, because of the familial aspect of our design, it is possible that our results may reveal risk variants that cross-cuts specific psychiatric diagnoses [11–14].

Genetic studies of psychiatric disease have revealed associations with multiple rare and common risk variants with reduced penetrance, which may interact in complex ways with each other, with background genetics, and with environmental risks [15]. Based on results to date, we expect that suicide will follow this complex genetic architecture. In this study, the familial analyses use a new statistical method (Shared Genomic Segments, SGS [16]) that is well-powered to identify rare genetic variants in large families, evidence that can then be used to prioritize searches for additional variants contributing to risk in other case samples. This design is complementary to the Genome-Wide Association Study (GWAS) approach in large case-control samples, which can also produce statistical evidence for risk genes to be followed up in independent case samples.

Using genome-wide single-nucleotide polymorphism (SNP) variants matched to the same variants in publicly available population control data, we identified regions of the genome that segregate in suicide cases within high-risk families. These statistically significant regions provide compelling genes as targets for follow-up. Although such follow-up studies would ideally use comprehensive sequence data, the SNP array platform used in this study contains putatively functional variants of high interest to psychiatric and medical disorders, both of which may share overlapping suicide risk. Functional content of the PsychArray was investigated first within familial cases responsible for the significant regions, and then within ~ 1300 additional Utah suicide cases unrelated to the original extended families analyzed.

This study adds to the growing knowledge of genetic risk for suicide. First, we have identified genes in regions of significant familial segregation in large high-risk families, providing replication for previously reported genes of high interest, and identifying target genes for additional follow-up. Second, we have identified novel risk variants using a large follow-up association analysis of PsychArray variants with predicted functional impact using a population-matched resource of suicide cases.

## Materials and methods

### Sample

This project is possible because of a collaboration with the Utah State Office of the Medical Examiner (OME), which has spanned two decades. With Institutional Review Board (IRB) permissions from the University of Utah, the Utah Department of Health and Utah Intermountain Healthcare, we have collected de-identified DNA samples from consecutive suicides since 1997. The collection numbers 4585 (3632 males and 953 females). DNA was extracted from blood using the Qiagen Autopure LS automated DNA extractor (www.qiagen.com). Identifying information from cases with DNA was linked to data within the UPDB’s secure computer servers. All identifying data were then stripped before providing data to the research team; suicide cases and family structure data are referenced by anonymous IDs. DNA for this research project is shared with the NIMH Repository and Genomics Resource, project number 315 (2880 samples are now at the repository; additional samples are being sent on an ongoing basis).

### Determination of familial risk, selection of families/cases

**Fig. 1:**
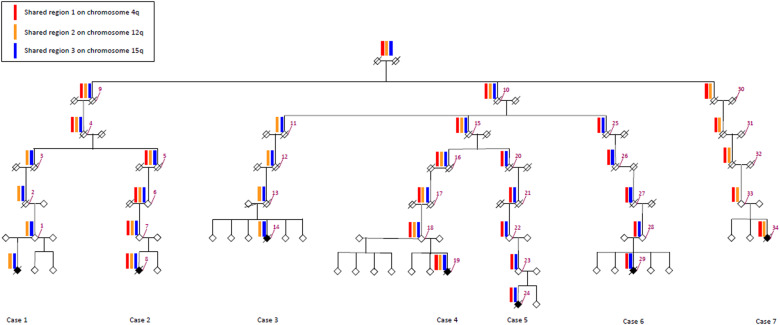
Extended structure of Family 66494 that links seven suicides (shaded in black) used for Shared Genomic Segment (SGS) analyses. Suicide cases are not as evident in upper generations because suicide status from death certificates is only available back to 1904. Note that gender is disguised and sibship order is randomized in order to protect the privacy of family members. Family size: there are 34 total meioses between the seven cases in this family; this counting is shown in purple on the drawing. SGS requires a total of at least 15 meioses between cases for adequate statistical power. Shared segments: three genomic segments provided significant evidence of sharing between cases in this family. The pattern of segregation of each segment is shown. Cases 2, 4, 5, 6, and 7 share region 1 (red). Cases 1, 2, 3, 4, 5, and 6 share region 2 (gold). Cases 1, 2, 3, 4, and 7 share region 3 (blue). Essential segregation is shown; however, when cases do not share, the region can actually be lost at any meiosis above the case in the family tree. The exact point of this loss is unknown

Genealogical data in the UPDB was used to construct family trees and identify those families at high risk for suicide. Beyond the suicide cases with DNA, the UPDB contains records of all known suicides from Utah death certificates dating from 1904 (*N* = 14,288). All 14,288 cases were used to estimate familial risk of suicide. To determine the extended families at highest risk, we used the Familial Standardized Incidence Ratio (FSIR) statistic [17], calculated by comparing the incidence of suicide in each extended family to its expected incidence determined by the statewide distribution for suicide stratified by sex and age. We identified 241 high-risk families containing significant excess of suicides (*p* < 0.05) and at least three suicides with DNA. We selected 43 of these families for analysis (Table 1) based on significance of the FSIR risk statistic, number of cases with DNA, and overall count of meioses between these cases (see Table 1). The 43 families included 2.04–4.41 times the expected number of suicide cases as reflected in the FSIR statistic (range *p* = 0.003–1E-12, average *p* = 0.0007). The average number of cases per family with genotyping was 6.2 (range 3–13), and the average number of meioses between analyzed cases was 29.6 (range 15–70; see Fig. 1 for an example of how meioses are counted in a family of moderate size from our resource). Family-specific significance thresholds for genomic sharing (see analysis section below) depend upon family size, structure, dispersion of cases, and number of cases analyzed. Permission for use of family structure data were granted by the Resource for Genetic and Epidemiologic Research (RGE, https://rge.utah.edu), the oversight committee for use of UPDB data.

**Table 1:** Characteristics of 43 extended families at high risk for suicide

Family	FSIR	FSIR *P* value	Total *N* obs. cases	Total *N* exp. cases	*N* cases in Study	*N* meioses between analyzed cases	SGS threshold: significant	SGS threshold: suggestive	≥ 1 Significant region	≥ 1 Suggestive region
709	2.69	< 0.0001	27	10.04	8	35	6.36E-08	6.41E-07	Yes	Yes
1881	3.12	< 0.0001	21	9.31	4	17	2.03E-06	3.00E-05		
2082	3.99	< 0.0001	19	4.77	3	15	3.93E-06	9.78E-05		
7785	2.78	< 0.0001	28	10.08	6	26	1.45E-07	1.82E-06		Yes
8556	2.85	< 0.0001	41	14.39	7	37	8.99E-08	1.01E-06		Yes
11593	2.31	0.0002	25	10.82	6	29	1.28E-07	1.70E-06		Yes
12291	2.43	0.0001	23	9.48	6	29	1.59E-07	1.96E-06		Yes
27251	2.43	0.0001	36	16.93	12	52	1.16E-09	1.41E-08		Yes
36667	3.61	0.0039	7	1.61	4	17	7.99E-07	1.56E-05		Yes
37661	2.06	0.0031	19	9.21	5	20	1.79E-06	1.91E-05		Yes
40780	3.61	0.0038	7	1.94	4	19	1.59E-06	2.57E-05		Yes
41469	2.29	0.0013	18	7.86	4	20	4.24E-06	5.86E-05		Yes
43035	2.46	< 0.0001	36	14.63	7	31	1.09E-07	1.15E-06		Yes
43580	2.45	0.0004	19	7.75	6	29	2.93E-07	3.08E-06		Yes
46547	2.48	< 0.0001	29	11.71	6	28	2.75E-07	2.92E-06		Yes
60205	2.26	0.0007	21	9.31	5	26	5.07E-07	6.52E-06	Yes	
66494	2.45	0.0001	24	9.8	7	34	5.45E-08	6.47E-07	Yes	Yes
68939	2.21	0.0007	22	9.97	8	41	3.66E-08	3.95E-07		Yes
91500	3.19	0.0003	13	4.07	4	16	1.24E-06	2.18E-05		
129334	2.75	< 0.0001	25	9.09	7	36	6.92E-08	8.32E-07		Yes
148039	3.65	0.0002	12	3.29	4	15	5.14E-06	5.73E-05		
176860	2.71	< 0.0001	43	15.88	9	39	3.28E-08	3.08E-07		Yes
185855	2.96	0.0003	15	5.06	6	25	2.46E-07	2.24E-06		Yes
209487	2.82	< 0.0001	26	9.23	7	32	1.19E-07	1.25E-06	Yes	Yes
233769	2.5	< 0.0001	33	13.19	11	50	1.58E-08	1.40E-07	Yes	Yes
265545	4.05	0.001	8	1.98	5	17	2.74E-06	3.82E-05		
540295	2.59	< 0.0001	43	16.59	5	31	1.73E-07	3.10E-06		Yes
540775	2.46	0.003	13	5.28	7	34	5.88E-08	7.45E-07	Yes	Yes
544252	3.21	0.0025	9	2.8	4	19	7.48E-07	1.43E-05		Yes
553615	2.04	< 0.0001	81	39.7	13	69	2.69E-10	3.74E-09	Yes	Yes
554151	2.38	0.003	14	5.89	7	32	9.98E-08	1.05E-06		
587072	2.39	0.003	14	5.86	5	25	1.39E-06	1.55E-05		Yes
590241	2.47	0.0004	19	7.7	8	38	2.86E-08	3.16E-07		Yes
595955	2.48	< 0.0001	39	15.73	7	39	5.35E-08	6.62E-07		Yes
601627	2.86	< 0.0001	69	24.14	12	70	7.06E-10	9.14E-09	Yes	Yes
603481	2.64	0.0003	18	6.83	9	37	5.09E-08	5.35E-07	Yes	Yes
622459	2.54	0.0001	22	8.68	4	18	1.75E-06	2.60E-05		Yes
755858	2.28	0.001	19	8.33	5	24	3.77E-07	5.48E-06		
756794	2.7	0.0002	18	6.68	5	24	3.94E-07	5.67E-06		Yes
791533	3.67	0.001	9	2.45	3	17	3.09E-06	8.38E-05		Yes
807334	2.5	0.0001	24	9.58	6	30	3.25E-07	3.57E-06	Yes	Yes
923763	4.41	< 0.0001	14	3.18	3	15	3.33E-06	8.86E-05		
957634	3.7	0.0001	12	3.24	3	15	3.22E-06	8.40E-05		Yes

The 198 families not selected for analysis in this study exhibited less-significant risk, and/or had too few cases with DNA, and/or had insufficient distance between cases. The average number of suicide cases per family with DNA in these 198 families was 3.23 (SD = 0.88, range 3–5). The average *p* value associated with these FSIRs was 0.0028 (SD = 0.0074, range 0.0458–1E-4).

### Diagnostic data

In addition to basic demographic and cause of death information, we had access to diagnostic data for psychiatric conditions associated with suicide using electronic medical records data through the UPDB. Codes were linked to case numeric case identifiers within the Utah Population Database; de-identified results were provided for analysis. Conditions were defined by groups of diagnostic codes aggregated according to the International Classification of Diseases (ICD) system (www.icd9data.com; see Supplemental Table S1 for the list of codes used to define diagnostic categories in this study). Importantly, because our cases are not derived from a clinical population, cases can exhibit with no co-occurring diagnoses. Missing diagnostic data can occur for many reasons, including: (1) existence of diagnostic codes other than the 359 codes in Table S1; (2) a case who did not seek medical attention for the psychiatric disorders in question owing to stigma, lack of insurance, cultural barriers, or other lack of access to services, age-related lack of recognition of pathology, or symptoms not perceived to require medical attention; (3) diagnostic data not contained in the UPDB, including diagnoses prior to the storage of electronic diagnoses, or diagnoses given out of state or outside the ~ 85% coverage of electronic medical records data available in the UPDB. We treated missing data as unknown rather than assuming the absence of pathology.

### Molecular data

The SGS analyses used variants from the Illumina Infinium PsychArray platform, version 1.0 (https://www.illumina.com/products/by-type/microarray-kits/infinium-psycharray.html) genotyped on 216 suicide cases in the 43 selected families. This PsychArray includes 265,000 common informative tag SNPs, 245,000 variants selected from exome sequencing studies of medical and psychiatric conditions, and 50,000 rare variants associated specifically with psychiatric conditions. Supplemental Figure S1 shows the use of genotype data for the study. Genotyped array content was oriented to 1000 Genomes Project data. For initial analyses of familial sharing, we included all variants contained in 1000 Genomes Project control data, omitting variants where orientation was ambiguous, and variants which were not polymorphic. Using PLINK [16], we also removed 17,058 variants with > 5% missing calls and 176 variants that failed Hardy–Weinberg equilibrium (*p* < 0.001). In addition, one case from family 553615 was removed owing to a low call rate (> 5% missing). Our initial familial analyses used 237,415 variants from 215 completed suicide cases to reveal familial variation that defines the boundaries of the segments shared among related cases. Rare putatively functional array variants meeting QC criteria, including psychiatric and medical disease-specific variants, were used to follow-up additional variants in the shared regions.

### Analysis

(See Supplemental Figure S1 for a flow diagram of the study). We began by using a new analytical method, Shared Genomic Segments (SGS) [18] developed for analyzing large high-risk families to identify subsets of cases that share regions beyond sharing expected by chance. SGS identifies excessive lengths of consecutive SNPs with allelic sharing between relatives to infer genomic segments that are inherited. Theoretically, chance inherited genomic sharing in distant relatives is extremely improbable; thus, the method has power in large families such as those in our study [19]. The significance of each shared segment is assessed empirically using gene-drop simulations (independent of case status) to create a null distribution of expected sharing within each family. The method assigns haplotypes to family founders according to a publicly available linkage disequilibrium map from 1000 Genomes European data, followed by simulated segregation through each specific family structure, repeated a minimum of 500,000 times. The observed sharing is compared with simulated sharing to determine significance. See Supplemental Figure S2 for a hypothetical simplified example of SGS sharing. Genome-wide significance thresholds are calculated specific to each family, as statistical power varies with family structure, number of cases, and distance between cases. Significance thresholds account for multiple testing and linkage disequilibrium, and also adjust for within-family heterogeneity by including adjustment for all possible subsets of within-family sharing among cases. Model fitting to determine theoretical genome-wide thresholds used these distributions of gene-drop results. The overwhelming majority of the genome will be null (does not contain a suicide risk variant); we acknowledge a slight conservative bias as these distributions also contain a small number of true positives [16]. The genome-wide significant threshold corresponds to a false-positive rate of 0.5 per genome per family, whereas the suggestive threshold corresponds to one false-positive result per genome per family. In this study, we report regions with family-specific genome-wide significant evidence, and regions overlapping in more than one family where family-specific evidence was at least genome-wide suggestive. *P* values for these overlapping multiple-family regions were approximated using Fisher’s combined probability test [20]. The SGS analysis software is freely available (https://uofuhealth.utah.edu/huntsman/labs/camp/analysis-tool/shared-genomic-segment.php).

Power of SGS was previously investigated for a range of genetic models involving rare variants in extended family data [19], showing appropriate power for large families with at least 15 meioses between cases. For all scenarios considered in this study, genome-wide association studies would have had negligible power. Given these results, we selected only extended families with at least 15 meioses between cases (see Table 1 and Fig. 1).

### Follow-up analyses

All follow-up work focused on the targeted set of genes identified by the significant SGS regions. Genes were considered within significant segments for follow-up if coding or regulatory sequence (defined using Genomic Regions Enrichment of Annotations Tool [21]) fell within the shared segment. Genes determined in previous research to be highly likely to represent false-positive results [22] were deleted (in our data, a cluster of 56 olfactory receptor genes in one region on chromosome 11, *FAT1, CTC-432M15.3*, and *TRIM51*). Three phases of follow-up work were pursued (see Figure S1).

### Corroborating evidence from the literature

As a first investigation of the genes indicated by significant SGS regions, we conducted a comprehensive search of the literature for all suicide-related risk genes against which to compare with SGS location-specific evidence. Suicide risk was identified by searching the Web of Science database for the terms: suicid* and gene* (captures variants including *suicide, suicidal, suicidality*, *gene, genetic*, etc.). We included reviews on the genetics of suicide as well as linkage, GWAS, candidate gene, expression and epigenetic studies. As secondary information about the suicide-related genes, we also queried DisGeNET [23, 24] for gene associations or involvement with neuropsychiatric disorders and inflammation owing to their known association with suicide risk [9, 10, 25].

### PsychArray variants in cases subsets in high-risk families with SGS sharing

The next phase of follow-up comprised a search of the specific familial cases that generated each SGS region using available array variants within each region to determine whether any particular array variant could be responsible each result. Although the variants available to us on the PsychArray are far from complete, a search of relatively rare coding-region variants provides an efficient, immediate, potentially interpretable screen of our results in lieu of large-scale sequencing data [26]. We therefore checked for sharing of the minor allele of non-synonymous variants within specific cases responsible for SGS results, and strictly within region boundaries. Because the SGS method is most powerful for the detection of rare familial variants, we selected a minor allele frequency < 10% in the publicly available Exome Aggregation Consortium (ExAC, www.exac.broadinstitute.org) European, non-Finnish data (matching in ancestry to our sample).

### Gene-based evidence in additional Utah suicide cases

The final follow-up phase focused on genes from SGS regions as targets for further study in additional sample resources. Although familial variants may be private to the extended family/families producing the SGS evidence, it is also possible that the evidence implicates genes with additional risk variants in independent case samples (allelic heterogeneity). We screened an independent cohort of Utah suicides for potential functional variants in SGS-targeted genes; this case sample most closely matches the discovery families, as it was derived from the Utah population, and is comprised of completed suicides. Owing to the same population ascertainment source, it is predicted to match the familial discovery sample regarding demographics and diagnostics. To maximize statistical power in our relatively small follow-up cohort of 1300 completed suicide cases, we focused on the potential to discover moderately penetrant, potentially interpretable functional causal variation. To this end, we used the following criteria to select variants: (1) in coding sequence of genes identified by the significant SGS regions, (2) non-synonymous and predicted to be damaging from either PolyPhen [27] or Sift [28], (3) minor allele frequency < 20% in ExAC European, non-Finnish data. We tested for significant allelic association compared with ExAC European, non-Finnish data using Fisher’s exact test, or with chi-square tests for variants with > 10 observed chromosomes with the minor allele in cases and controls. Tests for additional variants within SGS regions excluded suicide cases responsible for original sharing evidence in that region. Significance was adjusted for multiple tests.

## Results

### High-risk families

SGS analyses were performed on all 43 families. Most of the families (35/43 = 81.4%) showed at least one genome-wide suggestive region, and 10 families (23.3%) showed at least one genome-wide significant region. High-risk genealogies have additional complexity. Although the total number of cases across all families listed in Table 1 is 267, 52 of these cases occurred in multiple families (see Figure S3 for an example of this complexity). Because analyses to identify genomic shared regions are done within family, we included cases each time they occurred under each founder, as we do not know a priori where true sharing may occur. It is possible that cases share risk variant(s) from one set of founders with other cases in that family, but then also share other risk variant(s) with cases in a second family through connections with the other founding couple. The complexities in relationships may allow for future studies of gene × gene interactions once risk variants have been established.

Descriptive characteristics of the 215 independent discovery cases from the 43 families were compared with the other 4370 unselected Utah suicide cases with DNA. Within the 215 high-risk familial cases, 172 were male (80.0%), similar to the 79.2% rate in the unselected sample. Average age at death in the family sample was 34.28 years (standard deviation = 16.28), significantly lower than the average age of 40.01 years (standard deviation = 17.39) in the unselected sample (*t* = 4.74; *p* < 0.0001). Method of suicide in the family sample was predominantly gun-related (110/215 = 51.1%), followed by other violent methods (78/215 = 36.3%), then overdose (27/215 = 12.6%). These rates are similar to those in the unselected sample of 52.6%, 32.0%, and 15.3%, respectively. Death certificate data identified 212/215 cases as European-Non-Hispanic, and three as African-Non-Hispanic (one case each in families 41469, 233769, and 587072), similar to the unselected sample, where death certificate data identified 96.89% as European-Non-Hispanic. An ancestry principal component analysis of genotype data from the 215 cases and 1000 Genomes population data confirmed the three African ancestry cases (two showed Hispanic admixture), and identified 11 other cases with evidence of Asian and/or Hispanic ancestry, resulting in overall rates of 3.3% non-European and 5.6% Hispanic cases.

We found similar percentages of cases with presence of diagnoses from electronic records in the 215 familial cases as compared with the unselected 4370 cases, with the exception of an increase in cases with personality disorders and an increase in prior attempts/suicidal ideation in the familial sample. Percentages of cases with at least one code in each of the diagnostic categories vs. the unselected sample were as follows: depression, 41.4% vs. 36.2%; bipolar, 13.0% vs. 10.6%; anxiety, 23.3% vs. 22.8%; psychosis, 2.3% vs. 2.9%; substance use/abuse, 9.8% vs. 12.4%; personality disorders, 14.4% vs. 9.7% (chi-square = 5.06, *p* = 0.02); ADHD, 4.7% vs. 3.7%; previous attempts/ideation, 37.2% vs. 28.9% (chi-square = 6.82, *p* = 0.009).

### SGS results

SGS analyses revealed 16 single-family regions with genome-wide significance (Table 2). Several families generated more than one region; these were the larger families, where there was more opportunity for multiple different case subsets to show sharing evidence (see Fig. 1 for a specific example of sharing in family 66,494; see Supplemental Figure S4a for drawings of all families with genome-wide significance). Table 2 also presents 15 regions where sharing evidence overlapped across more than one family (Figure S4b); in each of these regions, the single-family evidence was at least at the genome-wide suggestive level. For the region on chromosome 5q23.3–q31.1, person 112,304 is a descendant of both 553,615 and 603,471, and person 95,765 is a descendant in both 553,615 and 176,860. To satisfy the independence requirement for computing the Fisher’s combined *p* value [20], we computed the *p* value for this region omitting these cases. There are 207 genes with coding or regulatory sequence in the 31 SGS regions (Table S2).

**Table 2:** SGS regions with (1) genome-wide significant evidence or (2) overlapping evidence in more than one family meeting at least suggestive significance^a^

Sharing families	Chromosome	Start	End	Region length	*N* sharing cases	*P* value^a^
709, 8556	1p34.2	40,433,771	40,555,321	121,550	6, 5	3.47E-12
791533, 540775	1q31.1–q31.2	190,694,813	191,590,362	895,549	3, 7	4.63E-09
601627	2p16.3	50,902,522	51,820,543	918,021	6	1.94E-10
176860, 11593	2q32.2–q32.3	191,029,604	192,020,729	991,125	5, 6	4.31E-12
601627	2q36.3 – q37.1	230,899,765	231,454,354	554,589	6	2.39E-10
553615	3p14.1	64,735,531	65,289,530	553,999	8	8.87E-11
129334, 11593	3q26.33	181,074,751	181,229,833	155,082	4, 5	7.94E-12
603481	4q26	117,379,825	118,257,841	878,016	7	3.08E-08
807334	4q28.3	131,561,136	132,902,055	1,340,919	5	2.02E-07
8556, 66494	4q35.1 – q35.2	187,072,383	187,513,585	441,202	4, 5	1.82E-12
553615^b^	5q23.3–q31.1	129,199,151	131,819,921	2,620,770	9	2.39E-10
553615, 603481, 176860^c^	5q23.3–q31.1	129,684,909	131,819,921	2,135,012	7, 7, 7	1.30E-18
601627	5q33.3 – q34	159,633,484	160,328,128	694,644	7	5.47E-10
553615	6q11.1 – q12	62,563,817	64,139,997	1,576,180	8	1.34E-10
60205	6q24.3	148,162,328	148,621,930	459,602	5	4.02E-07
601627	7p21.2	14,144,663	15,001,308	856,645	6	2.04E-10
957634, 595955	7q36.1	150,239,676	151,123,529	883,853	3, 4	6.44E-11
587072, 595955	8p23.1	9,157,884	10,032,894	875,010	4, 5	4.71E-11
233769	10p15.3	2,408,852	2,881,331	472,479	7	1.11E-09
11593, 8556	10p12.33	17,391,660	17,576,227	184,567	4, 5	3.11E-11
27251, 233769	10q21.3	67,735,584	68,057,063	321,479	7, 5	8.22E-15
209487	11p11.2 – q12.1	47,312,689	56,518,769	9,206,070	6	6.60E-08
540775	11q13.3	69,482,091	69,933,696	451,605	7	6.20E-09
209487, 66494	12q.12	41,899,312	42,298,882	399,570	5, 5	2.14E-12
709	13q12.3	29,886,987	30,492,217	605,320	7	1.86E-08
27251, 41469	13q14.2	48,526,833	49,283,795	756,962	5, 4	2.93E-12
590241, 601627	14q23.1 – q23.2	60,699,751	62,360,464	1,660,713	5, 8	5.91E-14
709	15q21.3 – q22.2	58,601,804	59,646,991	1,045,187	6	2.74E-08
66494	15q22.2	62,914,165	63,686,327	772,162	6	5.44E-08
27251, 233769	18q11.2	24,414,687	24,494,344	79,657	8, 6	5.22E-15
27251, 622459	19q13.12	35,836,530	36,136,449	299,919	8,3	2.89E-12

^a^For regions shared by > 1 family, *p* value was estimated using Fisher’s combined probability test [20].

^b^This region was significantly shared by 553615 on its own, but a smaller overlapping region was also shared by 553615, 603481, and 176860

^c^Two cases were omitted from family 553615 to satisfy the independence requirement for computing Fisher’s combined *p* value [20]. Significance thresholds were re-computed for family 553615 eliminating these cases. Person 112304 is a descendant of both 553615 and 603471, and person 95765 is a descendant of both 553615 and 176860

### Follow-up studies (see Figure S1 for overview)

1. Supporting literature evidence: we did not find any overlap between significant SGS regions and genomic regions identified by previous family-based linkage studies of suicidal behaviors (Table S3). At the gene level, we reviewed the 207 SGS-targeted genes, first investigating specific supporting evidence of suicide risk. From our comprehensive literature search, a total of 755 genes have been associated with suicide with varying levels of statistical support (Table S4). Eighteen SGS-targeted genes were among these 755 suicide-risk genes (see Table 3; also highlighted in Table S2; a detailed description of these 18 genes follows Table S2). Given an estimated number of ~ 19,000 genes in the human genome [29], we estimate that 755/19000 = 4% of genes in the genome have current evidence associated with suicide risk. If the SGS regions were a random sample of the genome and unassociated with the suicide phenotype, we would expect that only ~ 4% of the genes in SGS regions (8/207 genes) would have corroborating evidence from the literature. However, we found that 18/207 = 8.7% of genes had supporting literature evidence, a significantly greater number than expected by chance (*Z* = 2.41, *p* = 0.008). This result suggests that the SGS regions are indeed segments of the genome that are enriched for prior evidence of suicide risk.

**Table 3:** Genes in significant SGS regions with supporting evidence of association with suicide

Ensembl ID	Chr	Start	End	Gene name	Suicide associations; other neuropsychiatric associations^a^	Inflammation/immune associations	Families	Type of Sequence within region
ENSG00000162670	1	190066792	190446759	*BRINP3*	GWAS suicidal behavior [38]; smoking cessation	Peri-implantitis [39] and ulcerative colitis [40]	791533, 504775	Regulatory
ENSG00000150681	1	192127587	192154945	*RGS18*	GWAS suicidality in MDD [41]; anorexia; neuroticism	Controls platelet function [42]	791533, 504775	Regulatory
ENSG00000151689	2	191208196	191236391	*INPP1*	Candidate gene study in suicide attempts in patients with BD [43]; lithium response; autism; neurodevelopment	Systemic lupus erythematosus and Sjögren’s syndrome [44]	176860, 11593	Coding
ENSG00000115419	2	191745553	191830278	*GLS*	Decreased PM brain tissue gene expression, suicide, and depression [45]; glutamate synthesis; schizophrenia	Immune function [46]	176860, 11593	Coding
ENSG00000115415	2	191829084	191885686	*STAT1*	Increased gene expression in PM brain tissue of suicides [47]; dementia; Alzhimer’s	Autoinflammatory disorder [48], vascular inflammation [49], and autoimmune disorders [50, 51]	176860, 11593	Coding
ENSG00000164867	7	150688083	150711676	*NOS3*	Candidate gene study, suicide attempt, and aggression [52]; bipolar; major depression; schizophrenia	Asthma, inflammatory bowel disease, and arthritis [53]	957634, 595955	Coding
ENSG00000164885	7	150750899	150755617	*CDK5*	Gene expression, completed suicide [54]; autism; Alzheimer’s	Neuroinflammation [55]	957634, 595955	Coding
ENSG00000146926	7	150872785	150884919	*ASB10*	MDD and completed suicide [56]	Systemic sclerosis [57]	957634, 595955	Coding
ENSG00000013374	7	151038785	151075535	*NUB1*	Increased expression in peripheral blood of suicides with BD [58]; Parkinson’s	None known	957634, 595955	Coding
ENSG00000106615	7	151163098	151217206	*RHEB*	GWAS of antidepressant-emergent suicidal ideation [59]; pain threshold	Allergic asthma [60]	957634, 595955	Coding
ENSG00000106617	7	151253197	151574210	*PRKAG2*	MDD and completed suicide [56]; schizophrenia	Chronic inflammatory skin disease [61]	957634, 595955	Coding
ENSG00000026025	10	17270258	17279592	*VIM*	Increased PM brain tissue gene expression in suicides [62]; anorexia; bulimia; Alzheimer’s	Rheumatoid arthritis [63]	11593, 8556	Regulatory
ENSG00000183230	10	67672276	69455927	*CTNNA3*	GWAS of antidepressant-emergent suicidal ideation [59]; bipolar; major depression; Alzheimer’s; schizophrenia	Asthma [64]	27251, 233769	Coding
ENSG00000110092	11	69455855	69469242	*CCND1*	Decreased gene expression in veterans with suicide attempts [65]; major depression	Rheumatoid arthritis [66]	540775	Regulatory
ENSG00000151233	12	42475647	42538681	*GXYLT1*	Sequence study, MDD, and completed suicide [56]	None known	209487, 66494	Regulatory
ENSG00000102468	13	47405685	47471169	*HTR2A*	Candidate gene, gene expression, studies of suicidal behavior [67–74]; affective disorders; alcoholism; Alzheimer’s; anxiety; bipolar; eating disorders; pain; psychosis; schizophrenia; Tourette syndrome	Regulation of immune response [75]	27251, 41469	Regulatory
ENSG00000027075	14	61909885	62016549	*PRKCH*	Increased gene expression in veterans with suicide attempts [65]; major depression	Rheumatoid arthritis [76]	590241, 601627	Coding
ENSG00000128923	15	59063391	59154099	*MINDY2* (*FAM63B*)	Meta-analysis of three suicide cohorts [58]; cognition in schizophrenia	None known	709	Coding

^a^Broad evidence for additional neuropsychatric disease associations were obtained from a search of the HuGE Literature Finder, part of the Centers for Disease Control and Prevention Public Health Genomics Knowledge Base (v3.1; https://phgkb.cdc.gov/PHGKB/)
2. Studies of variants in specific cases giving SGS evidence: we selected the 431 non-synonymous PsychArray variants with either benign or damaging functional predictions falling strictly within the significant SGS regions with ExAC European, non-Finnish minor allele frequency of < 10%, reflecting the greater power of SGS to detect more rare risk variants. Considering each group of familial cases supporting each SGS signal, we screened the SNPs strictly within each region for sharing of the selected rare, non-synonymous array variants. There was no instance where this limited array content explained the identified SGS sharing.
3. Studies of variants in ~1300 independent population-based cases: additional population-ascertained Utah suicides with PsychArray genotype data were available for follow-up. This sample was well matched to the family discovery sample; a comparison of these two genotyped samples resulted in no significant demographic or diagnostic differences. These comparisons included the three variables that showed significant differences in our comparison between the family discovery sample and the larger Utah cohort of 4370 suicides with DNA described above. Specifically, in the follow-up sample, age at death was 34.96 (standard deviation = 16.76), percentage with personality disorders was 15.3%, and percentage with suicidal ideation/previous attempt was 36.9% (compared with 34.28 years, 14.4%, and 37.2%, respectively, in the discovery family sample).
  When we analyzed the selected 352 potentially damaging, relatively rare array variants within genes targeted by significant SGS regions, we found four variants with significantly increased presence of the minor allele compared with ExAC European non-Finnish frequencies, adjusting for multiple testing correction (Table 4: rs181058279, *p* = 5.45E-06; rs76215382, *p* = 8.48E-05; rs121908538, *p* = 3.14E-12; rs745918508, *p* = 5.40E-29). Specific characteristics of cases with each of these rare variants are described in Table S5. This evidence suggests rates of psychopathology similar to rates seen in the overall follow-up genotyped sample. Demographics were also similar, though cases with the *AGBL2* variant were significantly more likely to be female (chi-square = 7.82, *p* = 0.003).

**Table 4:** Putatively functional SNPs in target SGS genes with significantly elevated minor allele frequency in Utah suicide cases

Gene	SNP	Location^a^	change	Sift/polyphen	ExAC Euro non-Finn freq (chroms)^b^	Suicide freq (chroms)^c^	*p* value^d^	Function; disease association
*SP110*	rs181058279	chr2: 231033860	G:C; missense	Damaging; possibly damaging	0.00006 (4/66714)	0.0019 (5/2624)	5.45E-06	Gene transcription; [25] immune deficiency [31, 32]
*AGBL2*	rs76215382	chr11: 47711820	G:A; missense	Damaging; probably damaging	0.0148 (986/66668)	0.0247 (65/2622)	8.48E-05	ATP/GTP binding; brain structure and function [33]
*SUCLA2*	rs121908538	chr13: 48528645	A:G; missense	Damaging; probably damaging	0.00003 (2/66524)	0.0034 (9/2621)	3.14E-12	Mitochondrial protein, energy to synapse [34]
*APH1B*	rs745918508	chr15: 63594615	A; frameshift	LOF	0.00007 (5/66712)	0.0088 (23/2620)	5.40E-29	Transmembrane protein; Alzheimer’s [36]; and Parkinson’s [37] diseases

^a^Base pair location hg19 genome build

^b^Minor allele frequency and number of chromosomes with the minor allele from European non-Finnish samples in the Exome Aggregation Consortium (ExAC) data

^c^Minor allele frequency and number of chromosomes with the minor allele derived from 1294–1312 Utah suicide cases with Illumina PsychArray data. For each variant, cases responsible for the original sharing were excluded. No case with a rare allele among those shown in this table had a known relationship (< 15th degree of relatedness) to the original high- risk family with SGS evidence

^d^Comparison of UT suicide cases with the ExAC data; Fisher’s exact test used for rs181058279, rs121908538, and rs745918508; chi-square test used for rs76215382. *P* values exceed the significance threshold of 1.42E-04 correcting for 352 multiple tests

## Discussion

We have ascertained and studied a unique resource of 43 extended families at high risk for suicide. The design uses the distantly related, high-risk cases to magnify genetic effects, enrich for genetic homogeneity, and minimize shared environmental effects. Families were identified from cases sampled from population-wide ascertainment, resulting in a study design independent of specific psychiatric diagnosis. Cases in the high-risk families were significantly younger at death, by 5.73 years on average, perhaps reflecting enhanced familial genetic risk over and above accumulated environmental risks that may play a greater role in suicide at later ages. The follow-up sample of ~ 1300 genotyped cases more closely matched this familial discovery sample. The mean age at death was similarly young, 5.05 years younger than the unelected sample. The matching of the replication cohort may be due to the fact that we have thus far targeted our overall genotyping efforts to cases with increased evidence of at least one other extended relative who is at suicide risk. Diagnostic data, when present, suggested similar rates of psychopathology across our entire research resource, with somewhat elevated rates of personality disorders and of suicidal ideation and previous attempt in both the family discovery sample and the follow-up genotyped sample.

Cases in families were analyzed with a statistically powerful method, SGS [18], resulting in the identification of genome-wide significant regions likely to harbor risk variants. This family evidence implicated 207 genes for targeted follow-up. We found significant overlap with a comprehensive survey of 18 genes implicated in suicide, lending further support for these genes. Of note, 15 of these 18 genes also show previous associations with inflammatory conditions (Table 3), supporting accumulating evidence for a cross-association between inflammation and suicide risk [30]. Because our method discovers familial genomic regions, we also reviewed prior family linkage studies of suicide risk, but did not find overlaps. This result is perhaps not surprising owing to differences in ascertainment and outcome measures in these previous studies.

The additional rare disease-associated content of the array did not immediately reveal functional rare variants shared across cases responsible for the familial sharing. This result is likely due to the limited number of potentially risk-causing variants captured on the array; sequencing will be required to discover the causal variants shared across the high-risk discovery cases. Alternatively, one or more regions may be false positives.

SGS also provides target genes for other follow-up studies. Genes truly associated with suicide risk may harbor multiple risk-associated variants (allelic heterogeneity). By focusing our follow-up studies to find additional risk alleles to the much reduced number of high-interest target variants in genes identified by SGS, statistical power is increased. An independent population-based cohort of ~ 1300 Utah completed suicide cases, well matched for ascertainment, resulted in four variants associated with suicide (*SP110*, *AGBL2*, *SUCLA2*, and *APH1B*). *SP110* is part of a leukocyte-specific nuclear body protein complex, and likely plays a role in gene transcription [25]. It has been implicated in pathogen resistance and immunodeficiency [31, 32], and may relate to suicide risk through a growing body of evidence implicating immune risk and inflammation [30]. *AGBL2* is an ATP/GTP binding protein implicated in brain structure and function [33]. S*UCLA2* is a mitochondrial tricarboxylic acid cycle protein recently implicated in energy supply to the synapse [34], and is possibly associated with recent findings linking suicide risk and hypoxia [35]. *APH1B* is a transmembrane protein associated with risk of Alzheimer’s [36] and Parkinson’s [37] diseases.

Characteristics of cases with each of these four variants did not reveal any striking patterns of association with specific psychopathology, though cases with the *AGBL2* variant were significantly more likely to be female. Follow-up in additional research cohorts will be required to clarify diagnostic associations, and to replicate the association with gender found with the *AGBL2* variant.

### Limitations

Suicide cases were predominantly of Northern European ancestry, as verified with genotype data, so results may be limited to this race/ethnic group. The genome-wide background of the PsychArray contains ~ 265,000 common variants, which is relatively sparse for a genome-wide array. A denser array could have provided additional precision to region boundaries, or may have revealed that some regions were false positives. Diagnostic data were limited to available diagnoses in the electronic medical record. Cases with no diagnostic data are not assumed to have an absence of psychopathology. Rather, missing data more likely reflect either diagnoses outside the scope of our data resources, or lack of connection to services owing to insurance, stigma, cultural factors, or a perception that symptoms did not warrant treatment.

### Conclusions

Our study has found significant associations using only on the relatively rare, potentially functional variants captured on the PsychArray; these results have been discovered through a rigorous statistical prioritization and variant selection based only on functional annotation and frequency. As new data on our resource become available, it is likely that additional potential risk variation will be found. However, the current work has produced several important lines of evidence. First, the genome-wide significant SGS regions identify 207 target genes for suicide risk. Second, follow-up analyses of these regions in an independent population-based cohort of suicides highlighted four genes with potential functional risk variants, pending replication. Finally, the SGS regions contained 18 genes with corroborating evidence for suicide risk, suggesting these as strong candidates for future work.

## Electronic supplementary material

Supporting Information Legends

Supplemental Tables 1, 2, 3, 4, and 5

Supplementary Materials
