# Supplementary material for: Genome-wide significant regions in 43 Utah high-risk families implicate multiple genes involved in risk for completed suicide
Source: Mol Psychiatry. 2018 Oct 23;25(11):3077–90. doi: 10.1038/s41380-018-0282-3 (PMC6478563; doi:10.1038/s41380-018-0282-3)
Supplement: Supplementary file 1 — Supporting Information Legends [file 41380_2018_282_MOESM1_ESM.pdf]

Supporting Information Legends

Table S1. Diagnostic codes comprising possible co-occurring psychiatric conditions considered in this study. A case was defined as having evidence for a condition if one or more codes from the list below was present in the available electronic medical records data

Table S2. 207 genes with coding or regulatory sequence in regions with significance evidence for familial sharing from SGS analyses. Genes supporting evidence of suicide risk are highlighted in yellow. Psychiatric and/or neuronal associations are in bold type.

Table S3. Significant SGS regions in relation to previous linkage studies of suicidal ideation and behaviors.

Table S4. Genes with existing evidence for association with suicide risk from a comprehensive literature search.

Table S5. Case characteristics of follow-up Utah suicides with significant SNP findings. No cases were related (out to 15$^{th}$ degree) to any case in the high risk family responsible for SGS evidence for the region containing that gene.

Figure S1. Flow diagram of the study.

Figure S2. Simplified hypothetical data defining familial shared genomic segments.

Figure S3. Example of suicide cases with complex family relationships to more than one founding couple.

Figure S4a. Extended family structures linking cases used for SGS analyses in the 10 families with genome-wide significant results within a single family. All analyzed cases are represented by a numeric ID. Only suicide cases in the line of descent to analyzed cases are shown. Gender is disguised and sibship order is randomized in order to protect the privacy of family members. NOTE: Suicide cases are not as evident in upper generations because suicide status from death certificates is only available back to 1904.

**Figure S4b**. Detail of extended family structures in the families with SGS evidence of shared regions overlapping across more than one family. All regions shared across two families are presented in drawings i through xiv. The region on chromosome five, shared across three families, is presented in drawing xv.
