# Supplementary material for: Genome-wide significant regions in 43 Utah high-risk families implicate multiple genes involved in risk for completed suicide
Source: Mol Psychiatry. 2018 Oct 23;25(11):3077–90. doi: 10.1038/s41380-018-0282-3 (PMC6478563; doi:10.1038/s41380-018-0282-3)
Supplement: Supplementary file 2 — Supplemental Tables 1, 2, 3, 4, and 5 [file 41380_2018_282_MOESM2_ESM.pdf]

Table S1. Diagnostic codes comprising possible co-occurring psychiatric conditions considered in this study. A case was defined as having evidence for a condition if one or more codes from the list below was present in the available electronic medical records data.

**Major depression and related conditions**	
296.2	major depressive disorder single episode
296	affective disorders
296.2	major depressive disorder single episode unspecified degree
296.21	major depressive disorder single episode mild degree
296.22	major depressive disorder single episode moderate degree
296.23	major depressive disorder single episode severe degree without psychotic behavior
296.24	major depressive disorder single episode severe degree with psychotic behavior
296.25	major depressive disorder single episode in partial or unspecified remission
296.26	major depressive disorder single episode in full remission
296.3	major depressive disorder recurrent episode
296.3	major depressive disorder recurrent episode unspecified degree
296.31	major depressive disorder recurrent episode mild degree
296.32	major depressive disorder recurrent episode moderate degree
296.33	major depressive disorder recurrent episode severe degree without psychotic behavior
296.34	major depressive disorder recurrent episode severe degree with psychotic behavior
296.35	major depressive disorder recurrent episode in partial or unspecified remission
296.36	major depressive disorder recurrent episode in full remission
296.82	atypical depressive disorder
300.9	unspecified nonpsychotic mental disorder
311	depressive disorder not elsewhere classified
313.1	misery and unhappiness disorder specific to childhood/adolescence
300.4	dysthymic disorder
301.1	affective personality disorder
301.1	affective personality disorder unspecified
301.12	chronic depressive personality disorder
648.4	mental disorders complicating pregnancy childbirth or the puerperium
648.4	mental disorders of mother, unspecified episode
648.41	mental disorders of mother, delivered, unspecified antepartum condition
648.42	mental disorders of mother, delivered, with postpartum complication
648.43	mental disorders of mother, antepartum condition or complication
648.44	mental disorders of mother, postpartum condition or complication
**Bipolar Disorder and related conditions**	
296	bipolar I disorder, single episode, unspecified degree
296.01	bipolar I disorder, single episode, mild degree
296.02	bipolar I disorder, single episode, moderate degree
296.03	bipolar I disorder, single episode severe degree without psychotic behavior
296.04	bipolar I disorder, single episode severe degree with psychotic behavior
296.05	bipolar I disorder, single episode in partial or unspecified remission
296.06	bipolar I disorder, single episode in full remission
296.1	manic disorder recurrent episode
296.1	manic affective disorder recurrent episode unspecified degree
296.11	manic affective disorder recurrent episode mild degree

296.12	manic affective disorder recurrent episode moderate degree
296.13	manic affective disorder recurrent episode severe degree without psychotic behavior
296.14	manic affective disorder recurrent episode severe degree with psychotic behavior
296.15	manic affective disorder recurrent episode in partial or unspecified remission
296.16	manic affective disorder recurrent episode in full remission
296.4	bipolar I disorder, most recent episode (or current) manic
296.4	bipolar I disorder, most recent episode (or current) manic unspecified degree
296.41	bipolar I disorder, most recent episode (or current) manic mild degree
296.42	bipolar I disorder, most recent episode (or current) manic moderate degree
296.43	bipolar I disorder, most recent episode (or current) manic severe degree without psychotic behavior
296.44	bipolar I disorder, most recent episode (or current) manic severe degree with psychotic behavior
296.45	bipolar I disorder, most recent episode (or current) manic in partial or unspecified remission
296.46	bipolar I disorder, most recent episode (or current) manic in full remission
296.5	bipolar I disorder, most recent episode (or current) depressed
296.5	bipolar I disorder, most recent episode (or current) depressed unspecified degree
296.51	bipolar I disorder, most recent episode (or current) depressed mild degree
296.52	bipolar I disorder, most recent episode (or current) depressed moderate degree
296.53	bipolar I disorder, most recent episode (or current) depressed severe degree without psychotic behavior
296.54	bipolar I disorder, most recent episode (or current) depressed severe degree with psychotic behavior
296.55	bipolar I disorder, most recent episode (or current) depressed in partial or unspecified remission
296.56	bipolar I disorder, most recent episode (or current) depressed in full remission
296.6	bipolar I disorder, most recent episode (or current) mixed
296.6	bipolar I disorder, most recent episode (or current) mixed unspecified degree
296.61	bipolar I disorder, most recent episode (or current) mixed mild degree
296.62	bipolar I disorder, most recent episode (or current) mixed moderate degree
296.63	bipolar I disorder, most recent episode (or current) mixed severe degree without psychotic behavior
296.64	bipolar I disorder, most recent episode (or current) mixed severe degree with psychotic behavior
296.65	bipolar I disorder, most recent episode (or current) mixed in partial or unspecified remission
296.66	bipolar I disorder, most recent episode (or current) mixed in full remission
296.7	bipolar I disorder, most recent episode (or current) unspecified
296.8	other and unspecified bipolar disorders
296.8	bipolar disorder, unspecified
296.81	atypical manic disorder
296.89	bipolar disorder, other
296.9	other and unspecified episodic mood disorder
296.9	unspecified episodic mood disorder
296.99	other specified episodic mood disorder
301.13	cyclothymic disorder
301.11	chronic hypomanic personality disorder
**Anxiety disorders**	
300	anxiety states
300	anxiety state, unspecified
300.01	panic disorder without agoraphobia
300.02	generalized anxiety disorder
300.09	other anxiety states
300.1	dissociative, conversion, and factitious disorders

300.1	hysteria, unspecified
300.11	conversion disorder
300.12	dissociative amnesia
300.13	dissociative fugue
300.14	dissociative identity disorder
300.15	dissociative disorder or reaction, unspecified
300.2	phobia, unspecified
300.21	agoraphobia with panic disorder
300.22	agoraphobia without panic attacks
300.23	social phobia
300.29	other isolated or specific phobias
300.3	obsessive-compulsive disorders
313	overanxious disorder, childhood/adolescence
313.2	sensitivity shyness and social withdrawal specific to childhood/adolescence
313.21	shyness disorder of childhood
313.22	introverted disorder of childhood
313.23	slective mutism
313.3	relationship problems specific to childhood/adolescence
300.6	depersonalization disorder
F44	Dissociative Disorders
308	acute reaction to stress
308	predominant disturbance of emotions
308.1	predominant disturbance of consciousness
308.2	predominant psychomotor disturbance
308.3	other acute reactions to stress
308.4	mixed disorders as reaction to stress
308.9	unspecified acute reaction to stress
309	adjustment disorder, depressive
309	adjustment reaction
309.1	adjustment reaction with prolonged depressive reaction
309.2	adjustment reaction with disturbance of other emotion
309.21	adjustment reaction with separation anxiety
309.22	adjustment reaction emancipation disorder of adolescence
309.23	adjustment reaction work inhibition
309.24	adjustment reaction anxiety
309.28	adjustment reaction mixed anxeity and depressed mood
309.29	adjustment reaction other emotions
309.3	adjustment reaction disturbance of conduct
309.4	adjustment reaction mixed emotion and conduct
309.8	adjustment reaction other specified reaction
309.82	adjustment reaction with physical symptoms
309.83	adjustment reaction with withdrawal
313.89	other emotional disturbances of childhood or adolescence
309.9	adjustment reaction unspecified
313.8	other or mixed emotional disturbances of childhood/adolescence
313.9	unspecified emotional disturbance of childhood/adolescence

301	paranoid personality disorder
301.21	introverted personality
313.82	identity disorder of childhood/adolescence
	**Psychotic disorders and related conditions**
295	schizophrenic disorders
295	simple type schizophrenia
295	simple type schizophrenia unspecified state
295.01	simple type schizophrenia subchronic state
295.02	simple type schizophrenia chronic state
295.03	simple type schizophrenia subchronic state with acute exacerbation
295.04	simple type schizophrenia chronic state with acute exacerbation
295.05	simple type schizophrenia in remission
295.1	disorganized type schizophrenia
295.1	disorganized type schizophrenia unspecified state
295.11	disorganized type schizophrenia subchronic state
295.12	disorganized type schizophrenia chronic state
295.13	disorganized type schizophrenia subchronic state with acute exacerbation
295.14	disorganized type schizophrenia chronic state with acute exacerbation
295.15	disorganized type schizophrenia in remission
295.2	catatonic type schizophrenia
295.2	catatonic type schizophrenia unspecified state
295.21	catatonic type schizophrenia subchronic state
295.22	catatonic type schizophrenia chronic state
295.23	catatonic type schizophrenia subchronic state with acute exacerbation
295.24	catatonic type schizophrenia chronic state with acute exacerbation
295.25	catatonic type schizophrenia in remission
295.3	paranoid type schizophrenia
295.3	paranoid type schizophrenia unspecified state
295.31	paranoid type schizophrenia subchronic state
295.32	paranoid type schizophrenia chronic state
295.33	paranoid type schizophrenia subchronic state with acute exacerbation
295.34	paranoid type schizophrenia chronic state with acute exacerbation
295.35	paranoid type schizophrenia in remission
295.4	schizophreniform disorder
295.4	schizophreniform disorder unspecified state
295.41	schizophreniform disorder subchronic state
295.42	schizophreniform disorder chronic state
295.43	schizophreniform disorder subchronic state with acute exacerbation
295.44	schizophreniform disorder chronic state with acute exacerbation
295.45	schizophreniform disorder in remission
295.5	latent schizophrenia
295.5	latent schizophrenia unspecified state
295.51	latent schizophrenia subchronic state
295.52	latent schizophrenia chronic state
295.53	latent schizophrenia subchronic state with acute exacerbation
295.54	latent schizophrenia chronic state with acute exacerbation

295.55	latent schizophrenia in remission
295.6	schizophrenic disorders, residual type
295.6	schizophrenic disorders, residual type unspecified state
295.61	schizophrenic disorders, residual type subchronic state
295.62	schizophrenic disorders, residual type chronic state
295.63	schizophrenic disorders, residual type subchronic state with acute exacerbation
295.64	schizophrenic disorders, residual type chronic state with acute exacerbation
295.65	schizophrenic disorders, residual type in remission
295.7	schizoaffective disorder
295.7	schizoaffective disorder, unspecified state
295.71	schizoaffective disorder, subchronic state
295.72	schizoaffective disorder, chronic state
295.73	schizoaffective disorder, subchronic state with acute exacerbation
295.74	schizoaffective disorder, chronic state with acute exacerbation
295.75	schizoaffective disorder, in remission
295.8	other specified types of schizophrenia
295.8	other specified types of schizophrenia, unspecified state
295.81	other specified types of schizophrenia, subchronic state
295.82	other specified types of schizophrenia, chronic state
295.83	other specified types of schizophrenia, subchronic state with acute exacerbation
295.84	other specified types of schizophrenia, chronic state with acute exacerbation
295.85	other specified types of schizophrenia, in remission
295.9	unspecified type schizophrenia
295.9	unspecified type schizophrenia, unspecified state
295.91	unspecified type schizophrenia, subchronic state
295.92	unspecified type schizophrenia, chronic state
295.93	unspecified type schizophrenia, subchronic state with acute exacerbation
295.94	unspecified type schizophrenia, chronic state with acute exacerbation
295.95	unspecified type schizophrenia, in remission
301.2	schizoid personality disorder
301.2	schizoid personality disorder, unspecified
301.22	schizotypal personality disorder
**Substance use and abuse, and related disorders**	
291	alcohol-induced mental disorders
291	alcohol withdrawal delirium
291.1	alcohol-induced persisting amnestic disorder
291.2	alcohol-induced persisting dementia
291.3	alcohol-induced psychotic disorder with hallucinations
291.4	idiosyncratic alcohol intoxication
291.5	alcohol-induced psychotic disorder with delusions
291.8	other specified alcohol-induced mental disorders
291.81	alcohol withdrawal
291.82	alcohol-induced sleep disorder
291.89	other alcoholic psychosis
291.9	unspecified alcohol-induced mental disorders
303	alcohol dependence syndrome

303	acute alcoholic intoxication in alcoholism, unspecified drinking behavior
303.01	acute alcoholic intoxication in alcoholism, continuous drinking behavior
303.02	acute alcoholic intoxication in alcoholism, episodic drinking behavior
303.03	acute alcoholic intoxication in alcoholism, in remission
303.9	other and unspecified alcohol dependence
303.9	other and unspecified alcohol dependence, unspecified drinking behavior
303.91	other and unspecified alcohol dependence, continuous drinking behavior
303.92	other and unspecified alcohol dependence, episodic drinking behavior
303.93	other and unspecified alcohol dependence, in remission
v61.41	alcoholism family
305	nondependent alcohol abuse
305.01	nondependent alcohol abuse, continuous drinking behavior
305.02	nondependent alcohol abuse, episodic drinking behavior
305.03	nondependent alcohol abuse, in remission
V11.3	personal history of alcoholism
304	drug dependence
304	opioid type dependence
304.1	sedative, hypnotic or anxiolytic dependence
304.2	cocaine dependence
304.3	cannabis dependence
304.4	amphetamine and other psychostimulant dependence
304.5	hallucinogen dependence
304.6	other specified drug dependence
304.7	combinations of opioid type drug with any other drug dependence
304.8	combinations of drug dependence excluding opioid type drug
304.9	unspecified drug dependence
305.2	nondependent cannabis abuse
305.3	nondependent hallucinogen abuse
305.4	nondependent sedative, hypnotic, or anxiolytic abuse
305.5	nondependent opioid abuse
305.6	nondependent cocaine abuse
305.7	nondependent amphetamine or related acting sympathomimetic abuse
305.8	nondependent antidepressant type abuse
v61.42	substance abuse in family
v65.42	counseling on substance use and abuse
**Personality disorders and related conditions**	
301.4	obsessive-compulsive personality disorders
301.6	dependent personality disorder
301.82	avoidant personality disorder
301.7	antisocial personality disorder
301.81	narcissistic personality disorder
313.83	academic underachievement disorder childhood/adolescence
301.83	borderline personality disorder
312	disturbance of conduct, not elsewhere classified
312.9	conduct disorder, unspecified
313.81	oppositional defiant disorder

301.5	histrionic personality disorder
301.59	other histrionic personality disorder
301.84	passive-aggressive personality disorder
301.89	other personality disorders
301.9	unspecified personality disorder
309.81	post-traumatic stress disorder
**ADHD and impulse-related disorders**	
301.3	explosive personality disorder
312.3	impulse control disorder, unspecified
312.31	pathological gambling
312.32	kleptomania
312.33	pyromania
312.34	intermittent explosive disorder
312.35	isolated explosive disorder
314	hyperkinetic syndrome of childhood
314	attention deficit disorder
314	attention deficit w/o hyperactivity
314.01	attention deficit w/ hyperactivity
314.9	hyperkinetic conduct disorder
v69.2	high-risk sexual behavior
v69.3	gambling and betting
**Previous suicide attempt/ideation**	
V62.84	suicidal ideation
E950	suicide and self-inflicted poisoning by solid or liquid substances
E950.0	suicide and self-inflicted poisoning by analgesics/antipyretics/antirheumatics
E950.1	suicide and self-inflicted poisoning by barbiturates
E950.2	suicide and self-inflicted poisoning by sedatives/hypnotics
E950.3	suicide and self-inflicted poisoning by tranquilizers/psychotropics
E950.4	suicide and self-inflicted poisoning by other specified drugs, medicinal substances
E950.5	suicide and self-inflicted poisoning by unspecified drug/medicinal substance
E950.6	suicide and self-inflicted poisoning by agricultural/horticultural chemical other than plant foods/fertilizers
E950.7	suicide and self-inflicted poisoning by corrosive/caustic substances
E950.8	suicide and self-inflicted poisoning by arsenic
E950.9	suicide and self-inflicted poisoning by other unspecified solid or liquid substances
E980.0	poisoning by analgesics, antipyreltics, and antirheumatics, motive undetermined
E980.1	poisoning by barbituates, motive undetermined
E980.2	poisoning by sedatives and hypnotics, motive undetermined
E980.3	poisoning by tranquilizers and other psychotropic agents, motive undetermined
E980.4	poisoning by other specified drugs and medicinal substances, motive undetermined
E980.7	poisoning by agricultural chemical preparations other than plant foods and fertilizers, motive undetermined
E980.8	poisoning by arsenic and its compounds, motive undetermined
E980.9	poisoning by other unspecified solid/liquid substance, motive undetermined
E980.5	poisoning by unspecified drug or medicinal substance, motive undetermined
E951	suicide and self-inflicted injury by gases in domestic use
E951.0	suicide and self-inflicted injury by gas distributed by pipeline
E951.1	suicide and self-inflicted injury by liquefied petroleum in mobile containers

E951.8	suicide and self-inflicted injury by other utility gas
E952	suicide and self-inflicted injury by other gases and vapors
E952.0	suicide and self-inflicted injury by motor vehicle exhaust gas
E952.1	suicide and self-inflicted injury by other carbon monoxide
E981	poisoning by gases in domestic use undetermined accidental/purposely inflicted
E982	poisoning by other gases/domestic use undetermined accidental/purposely inflicted
E952.8	suicide and self-inflicted injury by other specified gas
E952.9	suicide and self-inflicted injury by unspecified gas/vapor
E953	suicide and self-inflicted injury by hanging, strangulation, suffocation
E953.0	suicide and self-inflicted injury by hanging
E953.1	suicide and self-inflicted injury by suffocation by plastic bag
E953.8	suicide and self-inflicted injury by other specified means
E953.9	suicide and self-inflicted injury by unspecified means
E983	hanging/strangulation/suffocation undetermined
E984	drowning, undetermined intent
E954	suicide and self-inflicted injury by drowning
E955	suicide and self-inflicted injury by firearms/air guns/explosives
E955.0	suicide and self-inflicted injury by handgun
E955.1	suicide and self-inflicted injury by shotgun
E955.2	suicide and self-inflicted injury by hunting rifle
E985	injury firearms/explosives undetermined intent
E955.3	suicide and self-inflicted injury by military firearms
E955.4	suicide and self-inflicted injury by other/unspecified firearm
E955.6	suicide and self-inflicted injury by air gun
E955.7	suicide and self-inflicted injury by paintball gun
E955.9	suicide and self-inflicted injury by firearms/explosives, unspecified
E957	suicide and self-inflicted injury by jumping from high places
E957.0	suicide and self-inflicted injury by jumping from a residence
E957.1	suicide and self-inflicted injury by jumping from other man-made structures
E957.2	suicide and self-inflicted injury by jumping from natural sites
E957.9	suicide and self-inflicted injury by jumping from unspecified high place
E987	falling high place, undetermined intent
E958.0	suicide and self-inflicted injury by jumping or lying before a moving object
E955.5	suicide and self-inflicted injury by explosives
E958.1	suicide and self-inflicted injury by burns/fire
E958.2	suicide and self-inflicted injury by scald
E958.4	suicide and self-inflicted injury by electrocution
E958.7	suicide and self-inflicted injury by caustic substances (except poisoning)
E958.3	suicide and self-inflicted injury by extremes of cold
E958.5	suicide and self-inflicted injury by crashing of a motor vehicle
E958.6	suicide and self-inflicted injury by crashing of aircraft
E956	suicide and self-inflicted injury by cutting/piercing instrument
E986	injury cutting/piercing, undetermined intent
E988	injury, unspecified means, undetermined intent
E958.8	suicide and self-inflicted injury by other specific means
E958.9	suicide and self-inflicted injury by unspecified means

E959	late effects of self-injury
E989	late effects of self-injury, undetermined intent

Table S2. 207 Genes with coding or regulatory sequence in genomic regions with significant evidence for familial sharing from SGS analyses. Genes with previous associations with suicide risk are highlighted. Psychiatric and/or neuronal associations are in bold type.

ENSEMBL ID	chr	start	end	Gene Name	Association	Coding Sequence within Region
ENSG00000168389	1	40420802	40435638	***MFSD2A***	**intellectual disability; speech disorders**; neoplasms; microcephaly	YES
ENSG00000131236	1	40505905	40538321	*CAP1*	nephropathy; cardiovascular; cancer	YES
ENSG00000131238	1	40538379	40563375	***PPT1***	**many psychiatric diagnoses**	YES
ENSG00000162670	1	190066792	190446759	***BRINP3***	**suicide[4]; smoking cessation;** vascular inflammation; ulcerative colitis	NO
ENSG00000150681	1	192127587	192154945	***RGS18***	**suicide[3]; anorexia; neuroticism; neoplasms**	NO
ENSG00000170820	2	49189296	49381676	***FSHR***	**SSRI response**	NO
ENSG00000179915	2	50145643	51259674	***NRXN1***	**many psychiatric diagnoses**	YES
ENSG00000115239	2	53759810	54087170	*GPR75-ASB3*	unknown	NO
ENSG00000143942	2	53994929	54002333	*CHAC2*	cardiovascular disease	NO
ENSG00000187699	2	190744335	191068210	*C2orf88*	juvenile arthritis	YES
ENSG00000138379	2	190920423	190927455	*MSTN*	body weight	NO
ENSG00000198130	2	191054461	191208919	***HIBCH***	**cognitive delay; mental and motor retardation**	YES
ENSG00000151689	2	191208196	191236391	***INPP1***	**suicide[7]; lithium response; neurodevelopment; autism**	YES
ENSG00000151690	2	191273081	191373931	***MFSD6***	**ASD**	YES
ENSG00000189362	2	191369068	191399448	*TMEM194B (NEMP2)*	Unknown	YES
ENSG00000138386	2	191511472	191557492	***NAB1***	**neoplasms; nervous system diseases**	YES
ENSG00000115419	2	191745553	191830278	***GLS***	**suicide[6]; schizophrenia; glutamate synthesis**	YES
ENSG00000115415	2	191829084	191885686	***STAT1***	**suicide[5]; Alzheimer's, dementia;** COPD; sclerosis	YES
ENSG00000138378	2	191894302	192016322	***STAT4***	**neuronal survival/regeneration**; lupus; RA	YES
ENSG00000153827	2	230628554	230787955	***TRIP12***	**intellectual disability/ASD**	NO
ENSG00000153832	2	230787018	230877825	*FBXO36*	unknown	NO
ENSG00000163053	2	230899698	230933715	*SLC16A14*	immune diseases	YES
ENSG00000135899	2	231032009	231090444	*SP110*	immune diseases	YES
ENSG00000079263	2	231067826	231223762	*SP140*	immune diseases	YES
ENSG00000067066	2	231280657	231408805	*SP100*	immune diseases	YES
ENSG00000135932	2	231577560	231685790	*CAB39*	ischemic brain damage following stroke	NO
ENSG00000163638	3	64501333	64673676	***ADAMTS9***	**many psychiatric diagnoses**	NO
ENSG00000151276	3	65339200	66024509	***MAGI1***	**mood disorders; schizophrenia; neuroticism**	NO
ENSG00000205981	3	180701497	180707562	*DNAJC19*	metabolic disorder; cardiovascular disease; nervous system disease	NO
ENSG00000181449	3	181429714	181432221	***SOX2***	**many psychiatric diagnoses**	NO
ENSG00000270394	4	117220016	117221520	*MTRNR2L13*	unknown	NO
ENSG00000174599	4	118004718	118006736	***TRAM1L1***	**substance use disorders**; type II diabetes	YES
ENSG00000164100	4	118954773	119179803	***NDST3***	**schizophrenia; bipolar disorder**	NO
ENSG00000151466	4	129786076	130014764	***SCLT1***	**problem ETOH use**; cancer	NO
ENSG00000109794	4	187025573	187093821	*FAM149A*	unknown	YES
ENSG00000269302	4	187111913	187112626	*AC110771.1*	unknown	YES
ENSG00000145476	4	187112674	187134610	*CYP4V2*	cardiovascular; immune and nervous system diseases	YES
ENSG00000164344	4	187130133	187179625	*KLKB1*	cardiovascular disease	YES
ENSG00000088926	4	187187099	187210835	***F11***	**many psychiatric diagnoses**	YES
ENSG00000272297	4	187347700	187476464	***PRPF31*** *(RP11-215A19.2)*	**many psychiatric diagnoses**	YES
ENSG00000168412	4	187454809	187476721	***MTNR1A***	**many psychiatric diagnoses**	YES
ENSG00000198108	5	129240165	129522327	*CHSY3*	colorectal cancer	YES
ENSG00000169567	5	130494720	130507428	***HINT1***	**many psychiatric diagnoses**	YES

ENSG00000186687	5	130506503	130541119	***LYRM7***	**brain disease**	YES
ENSG00000158985	5	130581186	130734140	***CDC42SE2***	**schizophrenia**; neoplasms; immune system diseases	YES
ENSG00000158987	5	130759614	130970929	***RAPGEF6***	**schizophrenia;** breast cancer	YES
ENSG00000217128	5	130977407	131132710	***FNIP1***	**schizophrenia**; neoplasms	YES
ENSG00000164398	5	131142683	131347936	***ACSL6***	**many psychiatric diagnoses**	YES
ENSG00000164399	5	131396222	131398897	***IL3***	**many psychiatric diagnoses**	YES
ENSG00000164400	5	131409483	131411859	***CSF2***	**schizophrenia in females;** leukemia; inflammation; arthritis	YES
ENSG00000072682	5	131527531	131631008	*P4HA2*	arthritis; Chron's disease; neoplasms	YES
ENSG00000131435	5	131593364	131609147	*PDLIM4*	bone density; type II diabetes	YES
ENSG00000197208	5	131630136	131679899	***SLC22A4***	**Schizophrenia**	YES
ENSG00000197375	5	131705444	131731306	***SLC22A5***	**mood disorder;** carnitine deficiency	YES
ENSG00000197536	5	131746328	131811736	*C5orf56*	unknown	YES
ENSG00000125347	5	131817301	131826490	*IRF1*	Immune response; cancer	YES
ENSG00000170231	5	159626114	159665666	*FABP6*	diabetes; digestive system diseases	YES
ENSG00000135083	5	159680385	159738930	*CCNJL*	unknown	YES
ENSG00000145861	5	159776175	159797644	*C1QTNF2*	unknown	YES
ENSG00000221886	5	159820713	159822497	*ZBED8*	unknown	YES
ENSG00000164609	5	159830292	159842301	***SLU7***	**ASD; Parkinson's**	YES
ENSG00000164611	5	159849320	159855688	***PTTG1***	metabolic disorders; cancers; **ASD**	YES
ENSG00000118322	5	159992460	160115081	*ATP10B*	unknown	YES
ENSG00000255633	6	62284008	62284534	*MTRNR2L9*	unknown	NO
ENSG00000112232	6	62389865	62996132	***KHDRBS2***	**Alzheimers;** melanoma	YES
ENSG00000198225	6	63921351	63922929	*FKBP1C*	unknown	YES
ENSG00000146166	6	63985856	64029882	***LGSN***	**male-specific MDD**	YES
ENSG00000112245	6	64231666	64293492	***PTP4A1***	**stress/alcohol**	NO
ENSG00000203727	6	147830063	148058683	*SAMD5*	unknown	NO
ENSG00000111961	6	148593440	148873186	*SASH1*	skin; digestive system diseases	YES
ENSG00000111962	6	149068464	149398126	***UST***	**antidepressant response;** endometriosis	NO
ENSG00000122584	7	8473585	8792593	***NXPH1***	**many psychiatric diagnoses**	NO
ENSG00000189043	7	10971578	10979883	*NDUFA4*	**Leigh Disease**	NO
ENSG00000006468	7	13930853	14031050	***ETV1***	**neuronal regulation/migration**	NO
ENSG00000136267	7	14184674	15014402	***DGKB***	**many psychiatric diagnoses**	YES
ENSG00000179144	7	150211918	150218161	*GIMAP7*	diabetes; blood disorder	NO
ENSG00000133574	7	150264365	150271041	*GIMAP4*	immune system diseases; diabetes	YES
ENSG00000133561	7	150322463	150329473	*GIMAP6*	unknown	YES
ENSG00000106560	7	150382785	150390729	*GIMAP2*	unknown	YES
ENSG00000213203	7	150413645	150421372	*GIMAP1*	unknown	YES
ENSG00000196329	7	150419341	150447121	*GIMAP5*	immune system and digestive diseases; diabetes	YES
ENSG00000106565	7	150488373	150498448	***TMEM176B***	**Alzheimers; neoplasms**	YES
ENSG00000002933	7	150497491	150502208	*TMEM176A*	unknown	YES
ENSG00000002726	7	150521715	150558592	***AOC1***	**Parkinson's; schizophrenia**	YES
ENSG00000055118	7	150642049	150675403	***KCNH2***	**many psychiatric diagnoses**	YES
ENSG00000164867	7	150688083	150711676	***NOS3***	**suicide[10]; bipolar; major depression; schizophrenia;** inflammatory conditions; immune & autoimmune conditions	YES
ENSG00000181652	7	150709297	150721586	*ATG9B*	atherosclerosis; diabetes	YES
ENSG00000197150	7	150725510	150744869	*ABCB8*	neoplasms	YES
ENSG00000213199	7	150745379	150749843	*ASIC3*	hyperalgesia; fibromyalgia; chronic fatigue syndrome	YES
ENSG00000164885	7	150750899	150755617	***CDK5***	**suicide[11]; autism; Alzheimers; tobacco use disorder;** diabetes; Crohn's; obesity	YES
ENSG00000164889	7	150754297	150773614	*SLC4A2*	Liver disease; cancer	YES
ENSG00000164896	7	150773711	150777953	*FASTK*	blood disorder; deafness; neoplasms; myopathies	YES

ENSG00000164897	7	150778167	150780633	*TMUB1*	bone disease	YES
ENSG00000133612	7	150782918	150841523	***AGAP3***	**Schizophrenia**	YES
ENSG00000164900	7	150845676	150871832	*GBX1*	cancer	YES
ENSG00000146926	7	150872785	150884919	***ASB10***	**suicide[9];** scleroderma; pulmonary hypertension	YES
ENSG00000033050	7	150904923	150924316	*ABCF2*	neoplasms	YES
ENSG00000033100	7	150929575	150935908	*CHPF2*	unknown	YES
ENSG00000082014	7	150935850	150974982	*SMARCD3*	cardiovascular disease; neoplasms	YES
ENSG00000013374	7	151038785	151075535	***NUB1***	**suicide; Parkinson's; Huntington's;** diabetes	YES
ENSG00000187260	7	151072995	151107813	*WDR86*	unknown	YES
ENSG00000127377	7	151125921	151137899	*CRYGN*	unknown	YES
ENSG00000106615	7	151163098	151217206	***RHEB***	**suicide[8]; pain threshold**	YES
ENSG00000106617	7	151253197	151574210	***PRKAG2***	**suicide[9]; schizophrenia;** drug toxicity; cardiovascular disease; diabetes	YES
ENSG00000173281	8	8993765	9009084	*PPP1R3B*	digestive system diseases; cardiovascular diseases; diabetes	NO
ENSG00000253426	8	9009252	9025646	*RP11-10A14.4*	unknown	NO
ENSG00000173273	8	9413424	9639856	***TNKS***	**smoking behavior**; blood pressure; cancer	YES
ENSG00000175806	8	9911778	10286401	***MSRA***	**many psychiatric diagnoses**	YES
ENSG00000184647	8	10383056	10411676	***PRSS55***	**Parkinson's**	NO
ENSG00000185736	10	1228073	1779670	***ADARB2***	**dementia; substance disorders;** neoplasms	NO
ENSG00000067057	10	3108525	3179904	*PFKP*	digestive system diseases; metabolic disorders; neoplasms	NO
ENSG00000026025	10	17270258	17279592	***VIM***	**suicide[12]; anorexia; bulimia; Alzheimer's;** asthma; arthritis; Crohn's	NO
ENSG00000148488	10	17360382	17496329	*ST8SIA6*	unknown	YES
ENSG00000165996	10	17631958	17659376	***HACD1 (PTPLA)***	**Huntington's**	NO
ENSG00000165476	10	65281123	65384883	***REEP3***	**ASD**	NO
ENSG00000183230	10	67672276	69455927	***CTNNA3***	**suicide[8]; Alzheimer's; Bipolar; major depression; schizophrenia; tobacco use disorder;** inflammation; asthma; diabetes; cardiovascular disease	YES
ENSG00000198739	10	68685764	68859588	***LRRTM3***	**ASD; Alzheimer's**; amyloidosis	NO
ENSG00000025434	11	47269851	47290396	***NR1H3***	**Anorexia**	NO
ENSG00000110514	11	47290712	47351582	***MADD***	**Alzheimer's;** diabetes; cardiovascular diseases	YES
ENSG00000134571	11	47352957	47374253	*MYBPC3*	cardiovascular diseases	YES
ENSG00000066336	11	47376411	47400127	***SPI1***	**methylation abnormalities in ASD**	YES
ENSG00000165915	11	47428683	47438047	***SLC39A13***	**Psychosis;** cardiovascular disease; musculoskeletal disease	YES
ENSG00000165916	11	47440320	47447993	*PSMC3*	immune system diseases	YES
ENSG00000165917	11	47459308	47470730	*RAPSN*	myasthenic syndrome	YES
ENSG00000149187	11	47487496	47587121	***CELF1***	**Alzheimers;** neuromuscular disorder; MS; digestive and cardiovascular diseases	YES
ENSG00000213619	11	47586888	47606114	***NDUFS3***	**many psychiatric diagnoses**	YES
ENSG00000110536	11	47586982	47595013	*PTPMT1*	unknown	YES
ENSG00000123444	11	47593749	47600567	*KBTBD4*	unknown	YES
ENSG00000196666	11	47608198	47610746	*FAM180B*	unknown	YES
ENSG00000172247	11	47611216	47616211	*C1QTNF4*	unknown	YES
ENSG00000109919	11	47638867	47664175	***MTCH2***	**hippocampal dysfunction;** asthma; metabolic disorders	YES
ENSG00000165923	11	47681143	47736941	***AGBL2***	**mendelian neurologic disease;** arthritis	YES
ENSG00000109920	11	47738072	47788995	*FNBP4*	Unknown	YES
ENSG00000030066	11	47799639	47870107	***NUP160***	**Alzheimer's**	YES
ENSG00000149177	11	48002113	48189670	*PTPRJ*	leptin resistence; fibrosis; cancer	YES
ENSG00000182053	11	49050504	49059579	*TRIM49B*	cancer	YES
ENSG00000214891	11	49075266	49080664	*TRIM64C*	unknown	YES
ENSG00000086205	11	49168187	49230222	***FOLH1***	**many psychiatric diagnoses**	YES

ENSG00000172927	11	69061605	69182494	***MYEOV***	**ethanol consumption;** neoplasms	NO
ENSG00000110092	11	69455855	69469242	***CCND1***	**suicide[1]; major depression;** asthma; cardiovascular disease; rheumatoid arthritis	NOF
ENSG00000162344	11	69513000	69519410	***FGF19***	**regulates proneural genes;** Neoplams	YES
ENSG00000075388	11	69587797	69590171	*FGF4*	embryonic development; cell survival	YES
ENSG00000186895	11	69624992	69633792	***FGF3***	**sensory neuropathy**	YES
ENSG00000131620	11	69924408	70035634	*ANO1*	neoplasms; cystic fibrosis	YES
ENSG00000168040	11	70049269	70053496	***FADD***	**dementia; obesity**	NO
ENSG00000165966	12	41582250	41968392	*PDZRN4*	lung disease	YES
ENSG00000151233	12	42475647	42538681	***GXYLT1***	**suicide[9]**	NO
ENSG00000132938	13	29598748	30077892	***MTUS2***	**nervous system development**	YES
ENSG00000139514	13	30083547	30169825	*SLC7A1*	neoplasms; cardiovascular and digestive disease	YES
ENSG00000122042	13	30338508	30424821	*UBL3*	iron metabolism disorder	YES
ENSG00000102781	13	30776767	30881621	*KATNAL1*	Unknown	NO
ENSG00000102468	13	47405685	47471169	***HTR2A***	**suicide[13–20]; affective disorders; alcoholism; Alzheimer's; anxiety; bipolar; eating disorders; pain; psychosis; schizophrenia; Tourette syndrome**	NO
ENSG00000136143	13	48510622	48612125	***SUCLA2***	**many psychiatric diagnoses;** mitochondrial disorder	YES
ENSG00000136159	13	48611703	48621358	*NUDT15*	digestive system and skin diseases	YES
ENSG00000136146	13	48627459	48669267	*MED4*	endometriosis; neoplasms	YES
ENSG00000136156	13	48807294	48837063	***ITM2B***	**many psychiatric diagnoses**	YES
ENSG00000139687	13	48877887	49056122	***RB1***	**many psychiatric diagnoses**	YES
ENSG00000139679	13	48963707	49018840	*LPAR6*	alopecia; cancer	YES
ENSG00000136161	13	49063095	49107369	*RCBTB2*	immune system diseases; neoplasms	YES
ENSG00000152207	13	49280951	49283498	*CYSLTR2*	vagal sensation; inflammation response	YES
ENSG00000102531	13	49550048	49783915	***FNDC3A***	**Frontotemporal Lobar Degeneration; bipolar disorder**	NO
ENSG00000100614	14	60712566	60759200	*PPM1A*	amyloidosis; neoplasms	YES
ENSG00000179008	14	60903563	60951796	*C14orf39*	unknown	YES
ENSG00000184302	14	60976117	60977970	*SIX6*	development	YES
ENSG00000100625	14	61180125	61190792	*SIX4*	development	YES
ENSG00000020426	14	61201581	61435067	***MNAT1***	**many psychiatric diagnoses**	YES
ENSG00000126814	14	61441827	61447691	*TRMT5*	mitochondrial disorders	YES
ENSG00000139974	14	61447996	61519147	*SLC38A6*	Neoplasms	YES
ENSG00000182107	14	61746810	61747865	*TMEM30B*	neoplasms	YES
ENSG00000027075	14	61909885	62016549	***PRKCH***	**suicide[1]; major depression; tobacco use disorder;** rheumatoid arthritis	YES
ENSG00000100644	14	62162523	62214977	***HIF1A***	**many psychiatric diagnoses**	YES
ENSG00000023608	14	62229179	62261701	*SNAPC1*	neoplasms	YES
ENSG00000128918	15	58245622	58790065	***ALDH1A2***	**Schizophrenia; Alzheimer's**	YES
ENSG00000103569	15	58430368	58478110	***AQP9***	**response to TBI**	NO
ENSG00000166035	15	58702768	58861151	***LIPC***	**Alzheimer's;** dementia	YES
ENSG00000137845	15	58887403	59042177	***ADAM10***	**many psychiatric diagnoses**	YES
ENSG00000128923	15	59063391	59154099	***MINDY2 (FAM63B)***	**suicide; cognition in schizophrenia**	YES
ENSG00000157450	15	59157374	59389618	*RNF111*	asthma; cardiovascular diseases ; Neoplasms	YES
ENSG00000137776	15	59171244	59225852	*SLTM*	Unknown	YES
ENSG00000157456	15	59397277	59417244	*CCNB2*	Cell growth control; cancer	YES
ENSG00000157483	15	59427113	59665099	*MYO1E*	urogenital diseases; neoplasms; respiratory diseases	YES
ENSG00000171989	15	59499042	59500705	*LDHAL6B*	Neoplasms	YES
ENSG00000157470	15	59664892	59815748	*FAM81A*	Unknown	NO
ENSG00000171914	15	62682725	63136830	***TLN2***	**epilepsy;** neoplasms	YES
ENSG00000140416	15	63334831	63364114	*TPM1*	Cardiomyopathy	YES
ENSG00000103642	15	63413999	63434260	*LACTB*	Obesity	YES
ENSG00000185088	15	63418071	63450220	*RPS27L*	Neoplasms	YES

ENSG00000166128	15	63481668	63559981	***RAB8B***	**antidepressant effects; presenilin; depression;** digestive system diseases	YES
ENSG00000138613	15	63568217	63601325	***APH1B***	**Parkinson's; Alzheimer's;** cardiovascular diseases; immune diseases	YES
ENSG00000074410	15	63613577	63674360	*CA12*	hyperchlorhidrosis	YES
ENSG00000140455	15	63796793	63886839	*USP3*	heart failure	NO
ENSG00000134504	18	24034874	24237365	*KCTD1*	skin disorders; neoplasms	NO
ENSG00000171885	18	24432002	24445782	***AQP4***	**many psychiatric diagnoses**	YES
ENSG00000154080	18	24495595	24765281	*CHST9*	Neoplasms	NO
ENSG00000012124	19	35820072	35838264	***CD22***	**many psychiatric diagnoses**	YES
ENSG00000126266	19	35842455	35843357	*FFAR1*	diabetes; obesity; digestive system diseases; neoplasms	YES
ENSG00000185897	19	35849793	35850833	*FFAR3*	Inflammation; obesity	YES
ENSG00000126262	19	35940617	35941609	*FFAR2*	Inflammation	YES
ENSG00000188408	19	35978330	35981344	*KRTDAP*	Unknown	YES
ENSG00000161249	19	35989618	36004377	*DMKN*	skin disease; neoplasms	YES
ENSG00000189001	19	36014412	36019183	*SBSN*	Neoplasms	YES
ENSG00000105679	19	36024430	36036072	***GAPDHS***	**Alzheimer's**	YES
ENSG00000105677	19	36036642	36038349	*TMEM147*	Unknown	YES
ENSG00000105675	19	36041517	36054531	***ATP4A***	**many psychiatric diagnoses**	YES
ENSG00000249115	19	36103697	36113895	*HAUSS*	Unknown	YES
ENSG00000126254	19	36120056	36128456	*RBM42*	Unknown	YES
ENSG00000105672	19	36133363	36135754	*ETV2*	Unknown	YES

Detail of 18 genes with prior suicide evidence.

RGS18 and *BRINP3*, have regulatory sequence within the region on chromosome 1q31.1–31.2. *RGS18* is significantly associated with serious suicide attempts from a study of major depression,[1] and is also significantly associated with neuroticism and a mouse model of emotionality.[2] *RGS18* controls platelet function; platelets play an important role in inflammation.[3] *BRINP3* (*FAM5C*) is associated with suicidal ideation,[4] and also heart disease, vascular inflammation,[5] peri-implantitis[6] and ulcerative colitis.[7]

STAT1, GLS and *INPP1* have coding sequence in the chromosome 2q32.2-q32.3 shared region. *STAT1* is a transcription activator regulated by interferon-alpha (IFN-alpha); treatment with IFN-alpha is known to result in serious neuropsychiatric complications.[8] *STAT1* gene expression is significantly increased in individuals with severe depression, and in postmortem brain tissue of individuals who died by suicide.[9] *STAT1* has been implicated in the auto-inflammatory disorder, atypical neutrophilic dermatosis, [10] vascular inflammation[11] and has multiple immune system functions.[12,13] *GLS* is involved in removing glutamate, an excitatory neurotransmitter, from synapses in the brain and has downregulated expression in both suicide and depressed patients[14] and also influences immune function.[15] *INPP1* is involved intracellular signaling; *INPP1* variants are associated with an increased risk of suicide attempts in patients with bipolar disorder.[14]

INPP1 is also involved in lithium response[16] and with the autoimmine disorders, systemic lupus erythematosus and Sjögren's syndrome[17]

The genes *NUB1*, *RHEB*, *PRKAG2*, *ASB10*, *NOS3* and *CDK5* all have coding sequence in the chromosome 7q36.1 shared region. Of note, evidence for these co-located genes emerged from studies with different ascertainment and methods. *NUB1* has increased expression in the blood of males with bipolar disorder who died by suicide.[18] *RHEB* is a involved in rapamycin (mTOR) signaling and has been implicated in a study of suicidal thoughts following antidepressant treatment.[19] In addition, *RHEB* is part of the signaling pathway involved in the action of the antidepressant ketamine[20] and has been implicated in the induction of allergic asthma.[21] *PRKAG2* and *ASB10* have been identified as candidate genes in suicide cases with major depressive disorder through identification of rare disruptive protein variants in whole exome sequencing.[22] *PRKAG2* is associated with the chronic inflammatory skin disease, atopic dermatitis,[23] and *ASB10* with the autoimmune disorder, systemic sclerosis.[24] *NOS3* is involved in neurotransmission; variants in this gene have been associated with suicide behavior.[25] *NOS3* has also been implicated in asthma, inflammatory bowel disease and arthritis[26] The *CDK5* gene is involved in neuronal migration and synaptic plasticity; this gene has shown increased expression in the prefrontal cortex of individuals who died by suicide.[27] The CDK5 pathway has also been implicated in behavioral changes following chronic stressors in a mouse model,[28] in stress-induced neuronal cell death[29] and in neuroinflammation.[30]

VIM has regulatory sequence in the chromosome shared region 10p12.33. This gene encodes a cytoskeletal structural protein that maintains cell shape. *VIM* gene expression was found to be upregulated in the amygdala of suicide cases[31] and has been associated with schizophrenia,[32] Alzheimer's[33] and rheumatoid arthritis.[34]

The *CTNNA3* gene has coding sequence in the chromosome 10q21.3 shared region. A SNP in this gene was one of the most significant findings for suicide attempt in the large genome-wide study of suicidal behavior.[4] This gene has also been implicated in both heart disease and asthma,[35] again suggesting a role of inflammation risk in suicide.

CCND1 has regulatory sequence in the chromosome shared region 11q13.3 and has significantly decreased gene expression in veterans who have attempted suicide compared with veterans who have never attempted suicide.[36] *CCND1* has also been associated with Alzheimer's Disease[37] , schizophrenia[38] anxiety[39] and rheumatoid arthritis[40]

GXYLT1 is a xylosyltransferase that has regulatory sequence in the shared SGS region on chromosome 12q12. Whole exome sequencing of Hungarian suicide cases with major depressive disorder identified that four of 23 suicide

cases had rare variants in *GXYLT1* compared to controls without psychiatric diagnoses who did not die by suicide.[22]

The *HTR2A* serotonin receptor gene has regulatory sequence in the shared region on chromosome 13q14.2 and is considered a strong suicide biomarker with replicated functional evidence for involvement in suicidiality.[18] Serotonin is a neurotransmitter that has been implicated in both suicide and several psychiatric disorders.[41,42] Suicide risk may be tied to the association between *HTR2A* and response to antidepressant treatment.[43] *HTR2A* is also involved in the regulation of immune response.[44]

PRKCH has coding sequence in the chromosome shared region 14q23.1-q23.2; mutations in this gene are associated with susceptibility to cerebral infraction. *PRKCH* has significantly increased gene expression in veterans who have attempted suicide compared with veterans who have never attempted suicide.[36] *PRKCH* is also involved in rheumatoid arthritis[45]

MINDY2 (FAM63B) has coding sequence in the chromosome shared region 15q21.3 – q22.2. *MINDY2* had significantly decreased gene expression in a cohort of 45 suicide completers with varying psychiatric diagnoses[18] and is also associated with bipolar disorder[46] and schizophrenia.[47]

Table S3. Significant SGS regions in relation to previous linkage studies on suicidal ideation and behaviors.

Study	Analysis Type	Genome Wide Sig.	Chr.	Region	Marker	Region Start (GRCh37/hg19)	Region Stop (GRCh37/hg19)
This study	SGS	N	1	1p34.2	NA	40,433,771.00	40,555,321.00
This study	SGS	N	1	1q31.1 – q31.2	NA	190,694,813.00	191,590,362.00
Suicide attempts or a quantitative suicidality index in alcohol dependent families[30]	Linkage	N	1	1q41	D1S1602	215,095,408.00	215,295,710.00
This study	SGS	Y	2	2p16.3	NA	50,902,522.00	51,820,543.00
Recurrent early-onset MDD with suicide attempt included as a covariate[31]	Linkage	N	2	2p12	D2S1777	78,420,289.00	78,620,740.00
BD with suicide attempt included as a covariate[32]	Linkage	Y	2	2p12	D2S1777	78,420,289.00	78,620,740.00
Suicidality in MDD[33]	Linkage	N	2	2p12	D2S428	82,881,763.00	83,082,057.00
Suicide attempts or a quantitative suicidality index in alcohol dependent families[30]	Linkage	Y	2	2p11.2	D2S1790	84,975,280.00	85,175,634.00
BD, psychosis, suicidal behavior, panic disorder[34]	Linkage	N	2	2q24.1	D2S1353	159,458,931.00	159,659,082.00
This study	SGS	N	2	2q32.2 – q32.3	NA	191,029,604.00	192,020,729.00
This study	SGS	Y	2	2q36.3 – q37.1	NA	230,899,765.00	231,454,354.00
Suicidality in MDD[33]	Linkage	N	3	3p14.2	D3S1234	60,007,362.00	60,207,549.00
This study	SGS	Y	3	3p14.1	NA	64,735,531.00	65,289,530.00
This study	SGS	N	3	3q26.33	NA	181,074,751.00	181,229,833.00
Suicide attempts or a quantitative suicidality index in alcohol dependent families[30]	Linkage	N	3	3q26	D3S2398	189,320,989.00	189,521,304.00
BD, psychosis, suicidal behaviour, panic disorder[34]	Linkage	N	4	4p16.1	D4S2366	6,384,678.00	6,584,947.00
This study	SGS	Y	4	4q26	NA	117,379,825.00	118,257,841.00
This study	SGS	Y	4	4q28.3	NA	131,561,136.00	132,902,055.00
This study	SGS	N	4	4q35.1 – q35.2	NA	187,072,383.00	187,513,585.00
This study	SGS	Y	5	5q23.3 – q31.1	NA	129,199,151.00	131,819,921.00
This study	SGS	N	5	5q23.3 – q31.1	NA	129,684,909.00	131,819,921.00
Recurrent early-onset MDD with suicide attempt included as a covariate[31]	Linkage	Y	5	5q31 – q33	D5S1480	144,043,648.00	144,243,979.00
This study	SGS	Y	5	5q33.3 – q34	NA	159,633,484.00	160,328,128.00
BD, psychosis, suicidal behaviour, panic disorder[34]	Linkage	N	6	6q24.3	D6S1848	35,976,225.00	36,176,624.00
This study	SGS	Y	6	6q11.1 – q12	NA	62,563,817.00	64,139,997.00
Recurrent early-onset MDD with suicide attempt included as a covariate[31]	Linkage	N	6	6q12	D6S1053	64,490,155.00	64,690,642.00
This study	SGS	Y	6	6q24.3	NA	148,162,328.00	148,621,930.00

BD, psychosis, suicidal behaviour, panic disorder[34]	Linkage	N	6	6q25.2	D6S2436	154,036,056.00	154,236,509.00
This study	SGS	Y	7	7p21.2	NA	14,144,663.00	15,001,308.00
This study	SGS	N	7	7q36.1	NA	150,239,676.00	151,123,529.00
This study	SGS	N	8	8p23.1	NA	9,157,884.00	10,032,894.00
Recurrent early-onset MDD with suicide attempt included as a covariate[31]	Linkage	N	8	8p22 – p21	D8S1145	18,252,480.00	18,452,816.00
Suicidality in MDD[33]	Linkage	N	9	9p24.3	D9S1779	416,800.00	617,069.00
This study	SGS	Y	10	10p15.3	NA	2,408,852.00	2,881,331.00
This study	SGS	N	10	10p12.33	NA	17,391,660.00	17,576,227.00
This study	SGS	N	10	10q21.3	NA	67,735,584.00	68,057,063.00
BD, psychosis, suicidal behaviour, panic disorder[34]	Linkage	N	10	10q25.3	D10S1237	116,019,979.00	116,220,583.00
This study	SGS	Y	11	11p11.2 – q12.1	NA	47,312,689.00	56,518,769.00
This study	SGS	Y	11	11q13.3	NA	69,482,091.00	69,933,696.00
Recurrent early-onset MDD with suicide attempt included as a covariate[31]	Linkage	Y	11	11q25	D11S968	133,718,376.00	133,918,617.00
This study	SGS	N	12	12q12	NA	41,899,312.00	42,298,882.00
This study	SGS	Y	13	13q12.3	NA	29,886,987.00	30,492,217.00
This study	SGS	N	13	13q14.2	NA	48,526,833.00	49,283,795.00
This study	SGS	Y	15	15q21.3 – q22.2	NA	58,601,804.00	59,646,991.00
This study	SGS	Y	15	15q22.2	NA	62,914,165.00	63,686,327.00
Suicidality in MDD[33]	Linkage	N	15	15q23	D15S145	70,761,176.00	70,961,309.00
This study	SGS	N	18	18q11.2	NA	24,414,687.00	24,494,344.00
Suicidality in MDD[33]	Linkage	N	18	18q22.1	D18S979	65,810,092.00	66,010,388.00

Table S4. Genes with existing evidence for association with suicide risk (TableS4_Suicide-Genes-Literature.xlsx).

PUBMED ID	REFERENCE	SOURCE TYPE	GENE
26666204	Sokolowski et al. 2016	GWAS	A1CF
28769055	Tombacz et al. 2017	WES Study	AADACL2
25178164	Sokolowski et al. 2015	Review Article	ABCB1
29331709	Bozorgmehr et al. 2018	Review Article	ABCG1
28769055	Tombacz et al. 2017	WES Study	ABI1
25178164	Sokolowski et al. 2015	Review Article	ABI3BP
22059935	Galfalvy et al. 2013	GWAS study not in Catalog	ACCN1
29331709	Bozorgmehr et al. 2018	Review Article	ACE
28769055	Tombacz et al. 2017	WES Study	ACOT11
29331709; 28809398	Bozorgmehr et al. 2018; Niculescu et al. 2017	Review Article; Gene Expression	ACP1
28809398	Niculescu et al. 2017	Gene Expression	ACSM3
25178164	Sokolowski et al. 2015	Review Article	ACTB
25178164	Sokolowski et al. 2015	Review Article	ACTN2
28809398	Niculescu et al. 2017	Gene Expression	ADAL
28769055	Tombacz et al. 2017	WES Study	ADAMDEC1
26079190	Galfalvy et al. 2015	GWAS Catalog	ADAMTS14
25732952	Karanovic et al. 2015	Candidate gene	ADARB1
25178164	Sokolowski et al. 2015	Review Article	ADH1B
28809398	Niculescu et al. 2017	Gene Expression	ADIRF
28809398	Niculescu et al. 2017	Gene Expression	ADK
28809398	Niculescu et al. 2017	Gene Expression	ADORA1
28769055	Tombacz et al. 2017	WES Study	ADRA1A
27721799	Mirkovic et al. 2016	Review Article	ADRA2A
25178164	Sokolowski et al. 2015	Review Article	ADRA2B
25178164	Sokolowski et al. 2015	Review Article	ADRB2
25178164	Sokolowski et al. 2015	Review Article	ADRBK1
25178164	Sokolowski et al. 2015	Review Article	ADRBK2
28769055	Tombacz et al. 2017	WES Study	AFF2
28809398	Niculescu et al. 2017	Gene Expression	AGA
25178164	Sokolowski et al. 2015	Review Article	AGO1
28872639	Flory et al. 2017	Gene Expression	AGO2
29331709	Bozorgmehr et al. 2018	Review Article	AGT
28809398	Niculescu et al. 2017	Gene Expression	AHCYL1
28809398	Niculescu et al. 2017	Gene Expression	AHCYL2
28809398	Niculescu et al. 2017	Gene Expression	AIMP1
28809398	Niculescu et al. 2017	Gene Expression	AK2
28809398	Niculescu et al. 2017	Gene Expression	AKAP10
28809398	Niculescu et al. 2017	Gene Expression	AKAP13
28809398	Niculescu et al. 2017	Gene Expression	AKAP2
27721799	Mirkovic et al. 2016	Review Article	AKT1
28872639	Flory et al. 2017	Gene Expression	AKT1S1
27721799	Mirkovic et al. 2016	Review Article	AKTIP
25178164	Sokolowski et al. 2015	Review Article	ALDH2
28809398	Niculescu et al. 2017	Gene Expression	ALDH3A2
28809398	Niculescu et al. 2017	Gene Expression	ALDH7A1
28769055	Tombacz et al. 2017	WES Study	ALG13
21081163	Fiori et al. 2012	Gene Expression	AMD1
25178164	Sokolowski et al. 2015	Review Article	ANK3
28809398	Niculescu et al. 2017	Gene Expression	ANKMY1
28769055	Tombacz et al. 2017	WES Study	ANKRD2
23958961	Le-Niculescu et al. 2013	Gene Expression	AP1S2
28769055	Tombacz et al. 2017	WES Study	APLP2
25178164	Sokolowski et al. 2015	Review Article	APOB
29331709; 28809398	Bozorgmehr et al. 2018; Niculescu et al. 2017	Review Article; Gene Expression	APOE
20877300	Perroud et al. 2012	GWAS	APOO
23958961	Le-Niculescu et al. 2013	Gene Expression	ARHGAP15
28809398	Niculescu et al. 2017	Gene Expression	ARHGAP26
28769055	Tombacz et al. 2017	WES Study	ARHGEF17
28809398	Niculescu et al. 2017	Gene Expression	ARRB1
28769055	Tombacz et al. 2017	WES Study	ARRDC2
28769055	Tombacz et al. 2017	WES Study	ASB10
25178164	Sokolowski et al. 2015	Review Article	ASIC2
28872639	Flory et al. 2017	Gene Expression	ATF4
21041247	Perlis et al. 2010	GWAS	ATL2
28769055	Tombacz et al. 2017	WES Study	ATP11C
28809398	Niculescu et al. 2017	Gene Expression	ATP13A2
28809398	Niculescu et al. 2017	Gene Expression	ATP6V0E1
25178164	Sokolowski et al. 2015	Review Article	AUTS2
25178164	Sokolowski et al. 2015	Review Article	AVP
25178164	Sokolowski et al. 2015	Review Article	AVPR1B
28809398	Niculescu et al. 2017	Gene Expression	B2M

21041247	Perlis et al. 2010	GWAS Catalog	B3GALT5
28769055	Tombacz et al. 2017	WES Study	BACE1
28809398	Niculescu et al. 2017	Gene Expression	BCKDHB
28809398	Niculescu et al. 2017	Gene Expression	BCL2
27721799	Mirkovic et al. 2016	Review Article	BDNF
28809398	Niculescu et al. 2017	Gene Expression	BF114768
28769055	Tombacz et al. 2017	WES Study	BIK
28872639	Flory et al. 2017	Gene Expression	BMP6
28872639	Flory et al. 2017	Gene Expression	BMP7
28769055	Tombacz et al. 2017	WES Study	BOD1L1
28872639	Flory et al. 2017	Gene Expression	BRAF
28809398	Niculescu et al. 2017	Gene Expression	BRCC3
26079190	Galfalvy et al. 2015	GWAS Catalog	BRINP3
28769055	Tombacz et al. 2017	WES Study	BRMS1L
28809398	Niculescu et al. 2017	Gene Expression	C14orf180
23958961	Le-Niculescu et al. 2013	Gene Expression	C18orf54
28769055	Tombacz et al. 2017	WES Study	C1orf226
28769055	Tombacz et al. 2017	WES Study	C1QTNF7
28809398	Niculescu et al. 2017	Gene Expression	C20orf27
28769055	Tombacz et al. 2017	WES Study	C6
28809398	Niculescu et al. 2017	Gene Expression	C7orf73
26079190; 28809398	Galfalvy et al. 2015; Niculescu et al. 2017	GWAS Catalog; Gene Expression	C8orf74
25178164	Sokolowski et al. 2015	Review Article	CACNA1C
28769055	Tombacz et al. 2017	WES Study	CACNA2D4
28809398	Niculescu et al. 2017	Gene Expression	CALR
28769055	Tombacz et al. 2017	WES Study	CAND2
21041247	Perlis et al. 2010	GWAS	CAPN13
28809398	Niculescu et al. 2017	Gene Expression	CAT
25178164	Sokolowski et al. 2015	Review Article	CBR1
25917933	Zai et al. 2015	GWAS Catalog	CCDC7
28769055	Tombacz et al. 2017	WES Study	CCDC93
29331709	Bozorgmehr et al. 2018	Review Article	CCK
27721799	Mirkovic et al. 2016	Review Article	CCKBR
28872639	Flory et al. 2017	Gene Expression	CCND1
28809398	Niculescu et al. 2017	Gene Expression	CD109
28809398	Niculescu et al. 2017	Gene Expression	CD164
23958961	Le-Niculescu et al. 2013	Gene Expression	CD200R1
22059935	Galfalvy et al. 2013	GWAS study not in Catalog	CD300LB
22059935	Galfalvy et al. 2013	GWAS study not in Catalog	CD44
28809398	Niculescu et al. 2017	Gene Expression	CD47
23958961	Le-Niculescu et al. 2013	Gene Expression	CD84
28769055	Tombacz et al. 2017	WES Study	CDC34
28809398	Niculescu et al. 2017	Gene Expression	CDC42EP4
25178164	Sokolowski et al. 2015	Review Article	CDCA7L
25178164	Sokolowski et al. 2015	Review Article	CDH10
25178164	Sokolowski et al. 2015	Review Article	CDH12
22059935	Galfalvy et al. 2013	GWAS study not in Catalog	CDH13
28872639	Flory et al. 2017	Gene Expression	CDH2
25178164	Sokolowski et al. 2015	Review Article	CDH9
28769055	Tombacz et al. 2017	WES Study	CDK14
28809398	Niculescu et al. 2017	Gene Expression	CDKAL1
28769055	Tombacz et al. 2017	WES Study	CELSR3
28769055	Tombacz et al. 2017	WES Study	CENPC
28809398	Niculescu et al. 2017	Gene Expression	CENPH
23958961	Le-Niculescu et al. 2013	Gene Expression	CEP44
28769055	Tombacz et al. 2017	WES Study	CEP72
28769055	Tombacz et al. 2017	WES Study	CEP85L
28769055	Tombacz et al. 2017	WES Study	CES4A
28769055	Tombacz et al. 2017	WES Study	CFAP57
28769055	Tombacz et al. 2017	WES Study	CFAP70
25178164	Sokolowski et al. 2015	Review Article	CKB
28809398	Niculescu et al. 2017	Gene Expression	CLN5
28809398	Niculescu et al. 2017	Gene Expression	CLTA
28809398	Niculescu et al. 2017	Gene Expression	CNOT3
21081163	Fiori et al. 2012	Gene Expression	CNP
25178164	Sokolowski et al. 2015	Review Article	CNR1
28769055	Tombacz et al. 2017	WES Study	COL12A1
22059935	Galfalvy et al. 2013	GWAS study not in Catalog	COL14A1
28769055	Tombacz et al. 2017	WES Study	COL6A6
27721799	Mirkovic et al. 2016	Review Article	COMT
28769055	Tombacz et al. 2017	WES Study	CPEB2
28809398	Niculescu et al. 2017	Gene Expression	CRAYAB

29331709	Bozorgmehr et al. 2018	Review Article	CREB1
29331709	Bozorgmehr et al. 2018	Review Article	CRH
27721799	Mirkovic et al. 2016	Review Article	CRHBP
27721799	Mirkovic et al. 2016	Review Article	CRHR
27721799	Mirkovic et al. 2016	Review Article	CRHR1
27721799	Mirkovic et al. 2016	Review Article	CRHR2
23958961	Le-Niculescu et al. 2013	Gene Expression	CROT
25178164	Sokolowski et al. 2015	Review Article	CRP
28872639	Flory et al. 2017	Gene Expression	CSNK1G2
28872639	Flory et al. 2017	Gene Expression	CTGF
22030708	Menke et al. 2012	GWAS study not in Catalog	CTNNA3
28872639	Flory et al. 2017	Gene Expression	CTNNB1
25178164	Sokolowski et al. 2015	Review Article	CTSD
28809398	Niculescu et al. 2017	Gene Expression	CTTN
28769055	Tombacz et al. 2017	WES Study	CTTNBP2
28769055	Tombacz et al. 2017	WES Study	CWC27
28769055	Tombacz et al. 2017	WES Study	CXorf23
28872639	Flory et al. 2017	Gene Expression	CXXC4
22059935	Galfalvy et al. 2013	GWAS study not in Catalog	CYP19A1
29331709	Bozorgmehr et al. 2018	Review Article	CYP2C19
29331709	Bozorgmehr et al. 2018	Review Article	CYP2D6
28769055	Tombacz et al. 2017	WES Study	D2HGDH
28809398	Niculescu et al. 2017	Gene Expression	DAB2
25178164	Sokolowski et al. 2015	Review Article	DBH
25178164	Sokolowski et al. 2015	Review Article	DBI
23958961	Le-Niculescu et al. 2013	Gene Expression	DCAF5
28769055	Tombacz et al. 2017	WES Study	DCC
29331709	Bozorgmehr et al. 2018	Review Article	DDC
28769055	Tombacz et al. 2017	WES Study	DEDD2
25178164	Sokolowski et al. 2015	Review Article	DGCR8
28769055	Tombacz et al. 2017	WES Study	DGKA
25178164	Sokolowski et al. 2015	Review Article	DHCR7
22059935	Galfalvy et al. 2013	GWAS study not in Catalog	DISC1
28809398	Niculescu et al. 2017	Gene Expression	DLG1
28769055	Tombacz et al. 2017	WES Study	DLG2
25178164	Sokolowski et al. 2015	Review Article	DLK1
25178164	Sokolowski et al. 2015	Review Article	DLK2
28809398; 25178164	Niculescu et al. 2017; Sokolowski et al. 2015	Gene Expression; Review Article	DLL1
25178164	Sokolowski et al. 2015	Review Article	DLL4
28769055	Tombacz et al. 2017	WES Study	DMD
28769055	Tombacz et al. 2017	WES Study	DNAH5
25178164	Sokolowski et al. 2015	Review Article	DNMT3B
28769055	Tombacz et al. 2017	WES Study	DOT1L
26079190	Galfalvy et al. 2015	GWAS Catalog	DPP10
25178164	Sokolowski et al. 2015	Review Article	DPYSL2
27721799	Mirkovic et al. 2016	Review Article	DRD2
29331709	Bozorgmehr et al. 2018	Review Article	DRD4
22059935	Galfalvy et al. 2013	GWAS study not in Catalog	DSC2
28809398	Niculescu et al. 2017	Gene Expression	DSPP
23958961	Le-Niculescu et al. 2013	Gene Expression	DTWD2
28809398	Niculescu et al. 2017	Gene Expression	DUSP13
28872639	Flory et al. 2017	Gene Expression	DVL1
28809398	Niculescu et al. 2017	Gene Expression	DYRK2
28769055	Tombacz et al. 2017	WES Study	EBF4
28809398	Niculescu et al. 2017	Gene Expression	ECHDC1
25178164	Sokolowski et al. 2015	Review Article	EFEMP1
28809398	Niculescu et al. 2017	Gene Expression	EFEMP2
25178164	Sokolowski et al. 2015	Review Article	EFHD2
28872639	Flory et al. 2017	Gene Expression	EIF1AX
28872639	Flory et al. 2017	Gene Expression	EIF2B1
28872639	Flory et al. 2017	Gene Expression	EIF3E
28872639	Flory et al. 2017	Gene Expression	EIF3F
28872639	Flory et al. 2017	Gene Expression	EIF3H
28872639	Flory et al. 2017	Gene Expression	EIF4G2
28769055	Tombacz et al. 2017	WES Study	ENST00000358775
28769055	Tombacz et al. 2017	WES Study	ENST00000376267
28769055	Tombacz et al. 2017	WES Study	ENST00000424383
28769055	Tombacz et al. 2017	WES Study	ENST00000520137
26079190	Galfalvy et al. 2015	GWAS Catalog	EPB41L4A
23958961	Le-Niculescu et al. 2013	Gene Expression	EPB41L5
28769055	Tombacz et al. 2017	WES Study	EPHA1
28769055	Tombacz et al. 2017	WES Study	EPS15

28769055	Tombacz et al. 2017	WES Study	ERCC5
23958961	Le-Niculescu et al. 2013	Gene Expression	ERP27
25178164	Sokolowski et al. 2015	Review Article	ESR1
25178164	Sokolowski et al. 2015	Review Article	ESR2
25178164	Sokolowski et al. 2015	Review Article	EVC
28769055	Tombacz et al. 2017	WES Study	EVPL
28809398	Niculescu et al. 2017	Gene Expression	EZR
25178164	Sokolowski et al. 2015	Review Article	FAAH
27721799	Mirkovic et al. 2016	Review Article	FABP5
28809398; 25178164	Niculescu et al. 2017; Sokolowski et al. 2015	Gene Expression; Review Article	FADS1
21423239	Willour et al. 2012	GWAS Catalog	FAM110C - LOC105373324
23958961	Le-Niculescu et al. 2013	Gene Expression	FAM173B
28769055	Tombacz et al. 2017	WES Study	FAM175A
28809398	Niculescu et al. 2017	Gene Expression	FAM63B
22030708	Menke et al. 2012	GWAS	FBXL18
28769055	Tombacz et al. 2017	WES Study	FFAR4
21081163	Fiori et al. 2012	Gene Expression	FGFR2
21081163	Fiori et al. 2012	Gene Expression	FGFR3
28809398	Niculescu et al. 2017	Gene Expression	FH
28809398	Niculescu et al. 2017	Gene Expression	FIGNL1
25178164	Sokolowski et al. 2015	Review Article	FKBP4
27721799	Mirkovic et al. 2016	Review Article	FKBP5
22059935	Galfalvy et al. 2013	GWAS study not in Catalog	FLJ23312
28769055	Tombacz et al. 2017	WES Study	FLJ45079
28809398	Niculescu et al. 2017	Gene Expression	FLOT2
25178164	Sokolowski et al. 2015	Review Article	FOXD4
22059935	Galfalvy et al. 2013	GWAS study not in Catalog	FOXN3
23958961	Le-Niculescu et al. 2013	Gene Expression	FOXP1
28769055	Tombacz et al. 2017	WES Study	FSD1L
28872639	Flory et al. 2017	Gene Expression	FZD5
28872639	Flory et al. 2017	Gene Expression	FZD9
28809398	Niculescu et al. 2017	Gene Expression	G2E3
21081163	Fiori et al. 2012	Gene Expression	GABARAPL1
25178164	Sokolowski et al. 2015	Review Article	GABRA1
25178164	Sokolowski et al. 2015	Review Article	GABRA2
21081163	Fiori et al. 2012	Gene Expression	GABRA4
21081163	Fiori et al. 2012	Gene Expression	GABRA5
21081163	Fiori et al. 2012	Gene Expression	GABRB3
21081163	Fiori et al. 2012	Gene Expression	GABRD
25178164	Sokolowski et al. 2015	Review Article	GABRG1
29331709	Bozorgmehr et al. 2018	Review Article	GABRG2
25178164	Sokolowski et al. 2015	Review Article	GABRG3
25178164	Sokolowski et al. 2015	Review Article	GABRP
21081163	Fiori et al. 2012	Gene Expression	GABRR1
29331709	Bozorgmehr et al. 2018	Review Article	GAD1
23958961	Le-Niculescu et al. 2013	Gene Expression	GANC
28872639	Flory et al. 2017	Gene Expression	GAS1
28872639	Flory et al. 2017	Gene Expression	GAS6
28809398	Niculescu et al. 2017	Gene Expression	GATM
20877300	Perroud et al. 2012	GWAS Catalog	GDA
28809398	Niculescu et al. 2017	Gene Expression	GDI2
25178164	Sokolowski et al. 2015	Review Article	GFAP
21750702	Schosser et al. 2011	GWAS study not in Catalog	GFRA1
21081163	Fiori et al. 2012	Gene Expression	GJA1
21081163	Fiori et al. 2012	Gene Expression	GLS
21081163	Fiori et al. 2012	Gene Expression	GLUD1
25178164	Sokolowski et al. 2015	Review Article	GLUL
28769055	Tombacz et al. 2017	WES Study	GMEB2
28872639	Flory et al. 2017	Gene Expression	GNA12
25178164	Sokolowski et al. 2015	Review Article	GNAI1
25178164	Sokolowski et al. 2015	Review Article	GNAI2
28769055	Tombacz et al. 2017	WES Study	GNAL
28872639	Flory et al. 2017	Gene Expression	GNAZ
28872639	Flory et al. 2017	Gene Expression	GNG8
21081163	Fiori et al. 2012	Gene Expression	GPM6B
27721799	Mirkovic et al. 2016	Review Article	GR1A3
21081163	Fiori et al. 2012	Gene Expression	GRABRB1
21081163	Fiori et al. 2012	Gene Expression	GRABRB3
25178164	Sokolowski et al. 2015	Review Article	GRIA1
21081163	Fiori et al. 2012	Gene Expression	GRIA2
25178164	Sokolowski et al. 2015	Review Article	GRIA3

21081163	Fiori et al. 2012	Gene Expression	GRIA4
21081163	Fiori et al. 2012	Gene Expression	GRIK1
27721799	Mirkovic et al. 2016	Review Article	GRIK2
25178164	Sokolowski et al. 2015	Review Article	GRIN2A
27721799	Mirkovic et al. 2016	Review Article	GRIN2B
21081163	Fiori et al. 2012	Gene Expression	GRINA
21081163	Fiori et al. 2012	Gene Expression	GRINL1A
24964207	Mullins et al. 2014	GWAS Catalog	GRIP1
21081163	Fiori et al. 2012	Gene Expression	GRM3
29331709; 28809398	Bozorgmehr et al. 2018; Niculescu et al. 2017	Review Article; Gene Expression	GSK3B
23958961	Le-Niculescu et al. 2013	Gene Expression	GTF3C2
28769055	Tombacz et al. 2017	WES Study	GXYLT1
28769055	Tombacz et al. 2017	WES Study	H2AFZ
28769055	Tombacz et al. 2017	WES Study	HAPLN1
23958961	Le-Niculescu et al. 2013	Gene Expression	HAVCR2
28872639	Flory et al. 2017	Gene Expression	HDGF
25178164	Sokolowski et al. 2015	Review Article	HES1
28769055	Tombacz et al. 2017	WES Study	HIPK2
28809398	Niculescu et al. 2017	Gene Expression	HIST1H2BO
27721799	Mirkovic et al. 2016	Review Article	HOMER1
28769055	Tombacz et al. 2017	WES Study	HR
25178164	Sokolowski et al. 2015	Review Article	HSPA8
27721799	Mirkovic et al. 2016	Review Article	HTR1A
27721799	Mirkovic et al. 2016	Review Article	HTR1B
25178164	Sokolowski et al. 2015	Review Article	HTR1E
27721799	Mirkovic et al. 2016	Review Article	HTR2A
25178164	Sokolowski et al. 2015	Review Article	HTR2B
29331709	Bozorgmehr et al. 2018	Review Article	HTR2C
25178164	Sokolowski et al. 2015	Review Article	HTR4
25178164	Sokolowski et al. 2015	Review Article	HTR6
23958961	Le-Niculescu et al. 2013	Gene Expression	HTRA1
28809398	Niculescu et al. 2017	Gene Expression	ICAM4
25178164	Sokolowski et al. 2015	Review Article	IFNG
28872639	Flory et al. 2017	Gene Expression	IGF2
28809398	Niculescu et al. 2017	Gene Expression	IGHG1
25178164	Sokolowski et al. 2015	Review Article	IL10
28872639; 28809398	Flory et al. 2017; Niculescu et al. 2017	Gene Expression; Gene Expression	IL13
23958961	Le-Niculescu et al. 2013	Gene Expression	IL1R1
25178164	Sokolowski et al. 2015	Review Article	IL2
28872639	Flory et al. 2017	Gene Expression	IL23A
19724245	Laje et al. 2009	GWAS study not in Catalog	IL28RA
28809398; 25178164	Niculescu et al. 2017; Sokolowski et al. 2015	Gene Expression; Review Article	IL6
25178164	Sokolowski et al. 2015	Review Article	IL8
29331709	Bozorgmehr et al. 2018	Review Article	IMPA2
25178164	Sokolowski et al. 2015	Review Article	INA
28769055	Tombacz et al. 2017	WES Study	ING5
29331709	Bozorgmehr et al. 2018	Review Article	ING-G
23958961	Le-Niculescu et al. 2013	Gene Expression	INO80D
29331709	Bozorgmehr et al. 2018	Review Article	INPP1
23958961	Le-Niculescu et al. 2013	Gene Expression	INPP4A
28872639	Flory et al. 2017	Gene Expression	ITGA8
28809398	Niculescu et al. 2017	Gene Expression	ITGB1BP1
28872639	Flory et al. 2017	Gene Expression	ITGB3
28872639	Flory et al. 2017	Gene Expression	ITGB5
23958961	Le-Niculescu et al. 2013	Gene Expression	ITLN1
28809398	Niculescu et al. 2017	Gene Expression	ITPKB
25178164	Sokolowski et al. 2015	Review Article	JAG1
25178164	Sokolowski et al. 2015	Review Article	JAG2
28809398	Niculescu et al. 2017	Gene Expression	JMJD1C
23958961	Le-Niculescu et al. 2013	Gene Expression	JRK
23958961	Le-Niculescu et al. 2013	Gene Expression	JUN
22059935	Galfalvy et al. 2013	GWAS study not in Catalog	KBTBD2
28809398	Niculescu et al. 2017	Gene Expression	KBTBD2
28769055	Tombacz et al. 2017	WES Study	KCNB1
20877300	Perroud et al. 2012	GWAS Catalog	KCNIP4
23958961	Le-Niculescu et al. 2013	Gene Expression	KCTD5
28769055	Tombacz et al. 2017	WES Study	KIAA1147
21750702	Schosser et al. 2011	GWAS study not in Catalog	KIAA1244
28769055	Tombacz et al. 2017	WES Study	KIAA1429
28769055	Tombacz et al. 2017	WES Study	KIAA1462
28769055	Tombacz et al. 2017	WES Study	KIAA1549
21423239	Willour et al. 2012	GWAS Catalog	KIAA1549L

28769055	Tombacz et al. 2017	WES Study	KIAA1875
23958961	Le-Niculescu et al. 2013	Gene Expression	KIR2DL4
28769055	Tombacz et al. 2017	WES Study	KLF7
28809398	Niculescu et al. 2017	Gene Expression	KLK7
28769055	Tombacz et al. 2017	WES Study	KRT77
28769055	Tombacz et al. 2017	WES Study	KRTAP2-4
28769055	Tombacz et al. 2017	WES Study	LAMA3
28809398	Niculescu et al. 2017	Gene Expression	LDLRAP1
28872639	Flory et al. 2017	Gene Expression	LEFTY2
28809398; 25178164	Niculescu et al. 2017; Sokolowski et al. 2015	Gene Expression; Review Article	LEPR
28809398	Niculescu et al. 2017	Gene Expression	LHFP
23958961	Le-Niculescu et al. 2013	Gene Expression	LINC00342
28769055	Tombacz et al. 2017	WES Study	LINC00632
25178164	Sokolowski et al. 2015	Review Article	LIPA
28769055	Tombacz et al. 2017	WES Study	LOC100507443
26079190	Galfalvy et al. 2015	GWAS Catalog	LOC101928174 - LOC100132824
26079190	Galfalvy et al. 2015	GWAS Catalog	LOC105372912
21041247	Perlis et al. 2010	GWAS Catalog	LOC105376477
25917933	Zai et al. 2015	GWAS Catalog	LOC107986892
28809398	Niculescu et al. 2017	Gene Expression	LPAR1
28872639	Flory et al. 2017	Gene Expression	LRP5
28872639	Flory et al. 2017	Gene Expression	LRP6
28769055	Tombacz et al. 2017	WES Study	LRRC37A4P
28769055	Tombacz et al. 2017	WES Study	LRRC74A
28809398	Niculescu et al. 2017	Gene Expression	LRRN3
25178164	Sokolowski et al. 2015	Review Article	LRRTM4
22059935	Galfalvy et al. 2013	GWAS study not in Catalog	LSAMP
25178164	Sokolowski et al. 2015	Review Article	MACROD2
28769055	Tombacz et al. 2017	WES Study	MAMLD1
28769055	Tombacz et al. 2017	WES Study	MAN1B1
27721799	Mirkovic et al. 2016	Review Article	MAOA
25178164	Sokolowski et al. 2015	Review Article	MAOB
28872639	Flory et al. 2017	Gene Expression	MAP2K2
28809398	Niculescu et al. 2017	Gene Expression	MAP2K5
28769055	Tombacz et al. 2017	WES Study	MAP3K14-AS1
28769055	Tombacz et al. 2017	WES Study	MAP3K15
25178164	Sokolowski et al. 2015	Review Article	MAP3K3
28872639	Flory et al. 2017	Gene Expression	MAP3K7
28872639	Flory et al. 2017	Gene Expression	MAPK2
28872639	Flory et al. 2017	Gene Expression	MAPK3
22059935; 28769055	Galfalvy et al. 2013; Tombacz et al. 2017	GWAS, WES	MARCH1
25178164	Sokolowski et al. 2015	Review Article	MARCKS
22059935	Galfalvy et al. 2013	GWAS study not in Catalog	MBNL2
21081163; 28809398	Fiori et al. 2012; Niculescu et al. 2017	Gene Expression; Gene Expression	MBP
28769055	Tombacz et al. 2017	WES Study	MCAM
28769055	Tombacz et al. 2017	WES Study	MCCD1
28769055	Tombacz et al. 2017	WES Study	MDN1
28809398	Niculescu et al. 2017	Gene Expression	MED28
23958961	Le-Niculescu et al. 2013	Gene Expression	METTL15
28769055	Tombacz et al. 2017	WES Study	MIA3
26666204	Sokolowski et al. 2016	GWAS	MIR548AA1
26666204	Sokolowski et al. 2016	GWAS	MIR548D1
28769055	Tombacz et al. 2017	WES Study	MIXL1
25178164	Sokolowski et al. 2015	Review Article	MLC1
28872639	Flory et al. 2017	Gene Expression	MMP7
28769055	Tombacz et al. 2017	WES Study	MNX1
23958961	Le-Niculescu et al. 2013	Gene Expression	MOB3B
21081163	Fiori et al. 2012	Gene Expression	MOG
28769055	Tombacz et al. 2017	WES Study	MPDZ
28769055	Tombacz et al. 2017	WES Study	MRAP2
28769055	Tombacz et al. 2017	WES Study	MRPL45
28809398	Niculescu et al. 2017	Gene Expression	MRPS14
28809398	Niculescu et al. 2017	Gene Expression	MRTO4
28809398	Niculescu et al. 2017	Gene Expression	MT1E
28809398	Niculescu et al. 2017	Gene Expression	MTERF4
25178164	Sokolowski et al. 2015	Review Article	MTHFR
25917933	Zai et al. 2015	GWAS Catalog	MTL3P
28769055	Tombacz et al. 2017	WES Study	MYBPC1
28872639	Flory et al. 2017	Gene Expression	MYC
22059935	Galfalvy et al. 2013	GWAS study not in Catalog	MYO3A
28809398	Niculescu et al. 2017	Gene Expression	N4BP2L2

21081163	Fiori et al. 2012	Gene Expression	NAPA
28769055	Tombacz et al. 2017	WES Study	NAV2
28769055	Tombacz et al. 2017	WES Study	NBEAP1
24964207	Mullins et al. 2014	GWAS Catalog	NCAM1
23958961	Le-Niculescu et al. 2013	Gene Expression	NDRG1
28809398	Niculescu et al. 2017	Gene Expression	NDUFS1
28809398	Niculescu et al. 2017	Gene Expression	NEAT1
21750702	Schosser et al. 2011	GWAS	NEBL
25178164	Sokolowski et al. 2015	Review Article	NEFL
25178164	Sokolowski et al. 2015	Review Article	NEFM
28809398	Niculescu et al. 2017	Gene Expression	NEK9
25178164	Sokolowski et al. 2015	Review Article	NGF
29331709; 28809398	Bozorgmehr et al. 2018; Niculescu et al. 2017	Review Article; Gene Expression	NGFR
28769055	Tombacz et al. 2017	WES Study	NHS
28769055	Tombacz et al. 2017	WES Study	NID2
28769055	Tombacz et al. 2017	WES Study	NINJ1
28769055	Tombacz et al. 2017	WES Study	NME1
29331709	Bozorgmehr et al. 2018	Review Article	NOS1
29331709	Bozorgmehr et al. 2018	Review Article	NOS3
25178164	Sokolowski et al. 2015	Review Article	NOTCH1
25178164	Sokolowski et al. 2015	Review Article	NOTCH2
25178164	Sokolowski et al. 2015	Review Article	NOTCH3
29331709	Bozorgmehr et al. 2018	Review Article	NOTCH4
22059935	Galfalvy et al. 2013	GWAS study not in Catalog	NPR3
25178164	Sokolowski et al. 2015	Review Article	NPR351
28769055	Tombacz et al. 2017	WES Study	NPSR1-AS1
27721799	Mirkovic et al. 2016	Review Article	NPTX2
29331709	Bozorgmehr et al. 2018	Review Article	NPY
25178164	Sokolowski et al. 2015	Review Article	NPY2R
28769055	Tombacz et al. 2017	WES Study	NR0B1
27721799	Mirkovic et al. 2016	Review Article	NR3C1
28769055	Tombacz et al. 2017	WES Study	NREP
28769055	Tombacz et al. 2017	WES Study	NSD1
25178164	Sokolowski et al. 2015	Review Article	NTF3
25178164	Sokolowski et al. 2015	Review Article	NTF4
27721799	Mirkovic et al. 2016	Review Article	NTKR2
25178164	Sokolowski et al. 2015	Review Article	NTRK2
28809398	Niculescu et al. 2017	Gene Expression	NUB1
28769055	Tombacz et al. 2017	WES Study	NUDCD3
23958961	Le-Niculescu et al. 2013	Gene Expression	NUDT10
20877300	Perroud et al. 2012	GWAS Catalog	NUGGC
27721799	Mirkovic et al. 2016	Review Article	ODC1
21081163; 28809398	Fiori et al. 2012; Niculescu et al. 2017	Gene Expression	OLIG1
28769055	Tombacz et al. 2017	WES Study	OPHN1
29331709; 25178164	Bozorgmehr et al. 2018; Sokolowski et al. 2015	Review Article; Review Article	OPRM1
28769055	Tombacz et al. 2017	WES Study	OTOG
28769055	Tombacz et al. 2017	WES Study	OTOGL
25178164	Sokolowski et al. 2015	Review Article	OXT
28809398	Niculescu et al. 2017	Gene Expression	PAFAH1B2
25178164	Sokolowski et al. 2015	Review Article	PAH
19724244	Laje et al. 2009	GWAS study not in Catalog	PAPLN
28769055	Tombacz et al. 2017	WES Study	PARD3B
28769055	Tombacz et al. 2017	WES Study	PBDC1
28809398	Niculescu et al. 2017	Gene Expression	PCDH9
28769055	Tombacz et al. 2017	WES Study	PCDHA2
25178164	Sokolowski et al. 2015	Review Article	PCDHB5
28769055	Tombacz et al. 2017	WES Study	PCDHB7
28769055	Tombacz et al. 2017	WES Study	PCSK5
28769055	Tombacz et al. 2017	WES Study	PCYOX1L
28872639	Flory et al. 2017	Gene Expression	PDGFA
23958961	Le-Niculescu et al. 2013	Gene Expression	PDXDC1
28769055	Tombacz et al. 2017	WES Study	PEMT
23958961	Le-Niculescu et al. 2013	Gene Expression	PER1
28769055	Tombacz et al. 2017	WES Study	PER2
25178164	Sokolowski et al. 2015	Review Article	PGAM1
28809398	Niculescu et al. 2017	Gene Expression	PGBD2
28809398	Niculescu et al. 2017	Gene Expression	PGK1
28769055	Tombacz et al. 2017	WES Study	PHF20
25178164	Sokolowski et al. 2015	Review Article	PIK3C2A
28872639; 23958961	Flory et al. 2017, Le-Niculescu et al. 2013	Gene Expression; Gene Expression	PIK3C3
28809398	Niculescu et al. 2017	Gene Expression	PIK3CA
28872639	Flory et al. 2017	Gene Expression	PIK3CD

28809398	Niculescu et al. 2017	Gene Expression	PIK3R1
28769055	Tombacz et al. 2017	WES Study	PIK3R4
23958961	Le-Niculescu et al. 2013	Gene Expression	PIP5K1B
28809398	Niculescu et al. 2017	Gene Expression	PITHD1
28809398	Niculescu et al. 2017	Gene Expression	PKP4
26079190	Galfalvy et al. 2015	GWAS Catalog	PLCB1
28769055	Tombacz et al. 2017	WES Study	PMCH
28769055	Tombacz et al. 2017	WES Study	PMM2
21081163	Fiori et al. 2012	Gene Expression	PMP22
28809398	Niculescu et al. 2017	Gene Expression	POLR2D
28872639	Flory et al. 2017	Gene Expression	POLR3GL
25178164	Sokolowski et al. 2015	Review Article	POMC
28872639	Flory et al. 2017	Gene Expression	PORCN
28809398	Niculescu et al. 2017	Gene Expression	PPAP2B
29331709	Bozorgmehr et al. 2018	Review Article	PPP1R1B
28872639	Flory et al. 2017	Gene Expression	PPP2R5B
23958961	Le-Niculescu et al. 2013	Gene Expression	PRCP
28769055	Tombacz et al. 2017	WES Study	PRDM16
25178164	Sokolowski et al. 2015	Review Article	PRDX6
25178164	Sokolowski et al. 2015	Review Article	PRKACA
28769055	Tombacz et al. 2017	WES Study	PRKAG2
28809398	Niculescu et al. 2017	Gene Expression	PRKAR1A
28872639; 28809398	Flory et al. 2017; Niculescu et al. 2017	Gene Expression; Gene Expression	PRKAR2B
25178164	Sokolowski et al. 2015	Review Article	PRKCA
28809398; 25178164	Niculescu et al. 2017; Sokolowski et al. 2015	Gene Expression; Review Article	PRKCB
21041247	Perlis et al. 2010	GWAS Catalog	PRKCE
25178164	Sokolowski et al. 2015	Review Article	PRKCG
28872639	Flory et al. 2017	Gene Expression	PRKCH
28809398	Niculescu et al. 2017	Gene Expression	PRKCI
24964207	Mullins et al. 2014	GWAS Catalog	PROM1
28769055	Tombacz et al. 2017	WES Study	PRR27
28769055	Tombacz et al. 2017	WES Study	PRSS56
28769055	Tombacz et al. 2017	WES Study	PRTG
28769055	Tombacz et al. 2017	WES Study	PSKH1
28872639	Flory et al. 2017	Gene Expression	PSMA1
28872639	Flory et al. 2017	Gene Expression	PSMA2
28872639	Flory et al. 2017	Gene Expression	PSMB1
28872639	Flory et al. 2017	Gene Expression	PSMB4
28872639	Flory et al. 2017	Gene Expression	PSMD7
26079190	Galfalvy et al. 2015	GWAS Catalog	PSME2
28809398	Niculescu et al. 2017	Gene Expression	PSME4
28809398	Niculescu et al. 2017	Gene Expression	PSNB4
25178164	Sokolowski et al. 2015	Review Article	PTEN
28769055	Tombacz et al. 2017	WES Study	PTGIS
28809398	Niculescu et al. 2017	Gene Expression	PTK2
25178164	Sokolowski et al. 2015	Review Article	PTPRR
25178164	Sokolowski et al. 2015	Review Article	QKI
23958961	Le-Niculescu et al. 2013	Gene Expression	R8M48
28769055	Tombacz et al. 2017	WES Study	RAB36
28769055	Tombacz et al. 2017	WES Study	RAB3GAP2
27721799	Mirkovic et al. 2016	Review Article	RABAC1
28769055	Tombacz et al. 2017	WES Study	RABGGTA
28872639	Flory et al. 2017	Gene Expression	RAC3
23958961	Le-Niculescu et al. 2013	Gene Expression	RAPH1
24964207	Mullins et al. 2014	GWAS Catalog	RARRES2
28769055	Tombacz et al. 2017	WES Study	RBM10
28769055	Tombacz et al. 2017	WES Study	RBM12B
28809398	Niculescu et al. 2017	Gene Expression	RBM3
28769055	Tombacz et al. 2017	WES Study	RBMXL2
28769055	Tombacz et al. 2017	WES Study	RERE
28769055	Tombacz et al. 2017	WES Study	REST
28769055	Tombacz et al. 2017	WES Study	RGL4
25178164	Sokolowski et al. 2015	Review Article	RGS18
29331709	Bozorgmehr et al. 2018	Review Article	RGS2
25178164	Sokolowski et al. 2015	Review Article	RGS4
22030708	Menke et al. 2012	GWAS study not in Catalog	RHEB
28809398	Niculescu et al. 2017	Gene Expression	RIMS3
28809398	Niculescu et al. 2017	Gene Expression	RNF6
28769055	Tombacz et al. 2017	WES Study	RNU6-71P
28769055	Tombacz et al. 2017	WES Study	RP2
28809398	Niculescu et al. 2017	Gene Expression	RPAP3
28769055	Tombacz et al. 2017	WES Study	RPE

28769055	Tombacz et al. 2017	WES Study	RPH3A
25917933	Zai et al. 2015	GWAS Catalog	RPL37P6
28872639	Flory et al. 2017	Gene Expression	RPS6
28872639	Flory et al. 2017	Gene Expression	RRAGA
25178164	Sokolowski et al. 2015	Review Article	S100A13
25178164	Sokolowski et al. 2015	Review Article	S100A8
21081163	Fiori et al. 2012	Gene Expression	S100B
29331709	Bozorgmehr et al. 2018	Review Article	SAT1
25178164	Sokolowski et al. 2015	Review Article	SAT2
28769055	Tombacz et al. 2017	WES Study	SBF1
28809398	Niculescu et al. 2017	Gene Expression	SCAF11
25178164	Sokolowski et al. 2015	Review Article	SCD
29331709	Bozorgmehr et al. 2018	Review Article	SCL1A3
28769055	Tombacz et al. 2017	WES Study	SCLY
25178164	Sokolowski et al. 2015	Review Article	SCN2B
27721799	Mirkovic et al. 2016	Review Article	SCN8A
28809398	Niculescu et al. 2017	Gene Expression	SECISBP2L
25178164	Sokolowski et al. 2015	Review Article	SELENBP1
28809398	Niculescu et al. 2017	Gene Expression	SEPT8
28872639	Flory et al. 2017	Gene Expression	SERPINF1
28809398	Niculescu et al. 2017	Gene Expression	SET
22059935	Galfalvy et al. 2013	GWAS study not in Catalog	SFRS11
28872639	Flory et al. 2017	Gene Expression	SHC3
28769055	Tombacz et al. 2017	WES Study	SHISA6
25073599	Guintivano	Methylation	SKA2
28769055	Tombacz et al. 2017	WES Study	SKOR2
26666204	Sokolowski et al. 2016	GWAS	SLC19A2
29331709	Bozorgmehr et al. 2018	Review Article	SLC1A2
21081163	Fiori et al. 2012	Gene Expression	SLC1A3
28809398	Niculescu et al. 2017	Gene Expression	SLC4A4
28809398	Niculescu et al. 2017	Gene Expression	SLC5A3
25178164	Sokolowski et al. 2015	Review Article	SLC6A1
29331709	Bozorgmehr et al. 2018	Review Article	SLC6A2
25178164	Sokolowski et al. 2015	Review Article	SLC6A3
27721799	Mirkovic et al. 2016	Review Article	SLC6A4
21041247	Perlis et al. 2010	GWAS	SLCA4
29331709	Bozorgmehr et al. 2018	Review Article	SLIT2
28769055	Tombacz et al. 2017	WES Study	SMAD3
23958961	Le-Niculescu et al. 2013	Gene Expression	SMARCA2
28769055	Tombacz et al. 2017	WES Study	SMARCC1
28809398	Niculescu et al. 2017	Gene Expression	SMCR8
25178164	Sokolowski et al. 2015	Review Article	SMOX
25178164	Sokolowski et al. 2015	Review Article	SMS
21081163	Fiori et al. 2012	Gene Expression	SNAP23
21081163	Fiori et al. 2012	Gene Expression	SNAP25
21081163	Fiori et al. 2012	Gene Expression	SNAP29
21081163	Fiori et al. 2012	Gene Expression	SNPH
25178164	Sokolowski et al. 2015	Review Article	SNTG2
28809398	Niculescu et al. 2017	Gene Expression	SNX6
28809398	Niculescu et al. 2017	Gene Expression	SOD2
28769055	Tombacz et al. 2017	WES Study	SOGA1
21041247	Perlis et al. 2010	GWAS Catalog	SORBS1
28769055	Tombacz et al. 2017	WES Study	SORL1
21041247	Perlis et al. 2010	GWAS	SPACA6
28769055	Tombacz et al. 2017	WES Study	SPAG17
28809398	Niculescu et al. 2017	Gene Expression	SPATA18
28769055	Tombacz et al. 2017	WES Study	SPATA31C2
28769055	Tombacz et al. 2017	WES Study	SPHKAP
28769055	Tombacz et al. 2017	WES Study	SPINK13
23958961	Le-Niculescu et al. 2013	Gene Expression	SPON1
28809398	Niculescu et al. 2017	Gene Expression	SPTBN1
22059935	Galfalvy et al. 2013	GWAS study not in Catalog	SPTLC1
28872639	Flory et al. 2017	Gene Expression	SRC
28769055	Tombacz et al. 2017	WES Study	SRRM4
25178164	Sokolowski et al. 2015	Review Article	SRSF11
28809398	Niculescu et al. 2017	Gene Expression	SSR
25178164	Sokolowski et al. 2015	Review Article	SST
28769055	Tombacz et al. 2017	WES Study	ST14
28769055	Tombacz et al. 2017	WES Study	STARD8
28769055	Tombacz et al. 2017	WES Study	STC2
26079190	Galfalvy et al. 2015	GWAS Catalog	STK3
28769055	Tombacz et al. 2017	WES Study	STRA8

28769055	Tombacz et al. 2017	WES Study	STX16-NPEPL1
21081163	Fiori et al. 2012	Gene Expression	STX1A
21081163	Fiori et al. 2012	Gene Expression	STXBP1
28809398	Niculescu et al. 2017	Gene Expression	SUMF2
28769055	Tombacz et al. 2017	WES Study	SURF4
21081163	Fiori et al. 2012	Gene Expression	SV2B
21081163	Fiori et al. 2012	Gene Expression	SYN2
21081163	Fiori et al. 2012	Gene Expression	SYNGR2
21081163	Fiori et al. 2012	Gene Expression	SYNJ2
21081163	Fiori et al. 2012	Gene Expression	SYNPO2
28809398	Niculescu et al. 2017	Gene Expression	SYNPO2L
21081163	Fiori et al. 2012	Gene Expression	SYPL
21081163	Fiori et al. 2012	Gene Expression	SYT1
21081163	Fiori et al. 2012	Gene Expression	SYT13
21081163	Fiori et al. 2012	Gene Expression	SYT4
21081163	Fiori et al. 2012	Gene Expression	SYT5
28769055	Tombacz et al. 2017	WES Study	SYTL3
29331709	Bozorgmehr et al. 2018	Review Article	TAAR6
28769055	Tombacz et al. 2017	WES Study	TAB3
25178164	Sokolowski et al. 2015	Review Article	TAC1
29331709	Bozorgmehr et al. 2018	Review Article	TACR1
28769055	Tombacz et al. 2017	WES Study	TATDN2
25178164	Sokolowski et al. 2015	Review Article	TBC1D1
25178164	Sokolowski et al. 2015	Review Article	TBC1D25
28872639	Flory et al. 2017	Gene Expression	TBL1X
28809398	Niculescu et al. 2017	Gene Expression	TBL1XR1
29331709	Bozorgmehr et al. 2018	Review Article	TBX19
26079190	Galfalvy et al. 2015	GWAS Catalog	TBX20
28809398	Niculescu et al. 2017	Gene Expression	TDG
28769055	Tombacz et al. 2017	WES Study	TECTA
28769055	Tombacz et al. 2017	WES Study	TFE3
28769055	Tombacz et al. 2017	WES Study	TG
25178164	Sokolowski et al. 2015	Review Article	TGFB1
28769055	Tombacz et al. 2017	WES Study	TGIF2LY
28809398; 25178164	Niculescu et al. 2017; Sokolowski et al. 2015	Gene Expression; Review Article	TGOLN2
29331709	Bozorgmehr et al. 2018	Review Article	TH
23958961	Le-Niculescu et al. 2013	Gene Expression	TIM1
28769055	Tombacz et al. 2017	WES Study	TIMP1
28872639	Flory et al. 2017	Gene Expression	TIMP4
28809398	Niculescu et al. 2017	Gene Expression	TM4SF1
28769055	Tombacz et al. 2017	WES Study	TMA16
21423239	Willour et al. 2012	GWAS Catalog	TMEM132C
22030708	Menke et al. 2012	GWAS study not in Catalog	TMEM138
28809398	Niculescu et al. 2017	Gene Expression	TMEM254
28769055	Tombacz et al. 2017	WES Study	TMPRSS11F
25917933	Zai et al. 2015	GWAS Catalog	TMX3
25178164	Sokolowski et al. 2015	Review Article	TNF
29331709	Bozorgmehr et al. 2018	Review Article	TNF-A
28769055	Tombacz et al. 2017	WES Study	TNFRSF11B
28769055	Tombacz et al. 2017	WES Study	TNS1
28769055	Tombacz et al. 2017	WES Study	TP53RK
28809398	Niculescu et al. 2017	Gene Expression	TPD52
28769055	Tombacz et al. 2017	WES Study	TPGS2
27721799	Mirkovic et al. 2016	Review Article	TPH1
27721799	Mirkovic et al. 2016	Review Article	TPH2
28809398	Niculescu et al. 2017	Gene Expression	TRAF3
28769055	Tombacz et al. 2017	WES Study	TRIM15
28809398	Niculescu et al. 2017	Gene Expression	TRIM23
28769055	Tombacz et al. 2017	WES Study	TRO
28769055	Tombacz et al. 2017	WES Study	TSC2
28769055	Tombacz et al. 2017	WES Study	TSPAN6
28809398	Niculescu et al. 2017	Gene Expression	TTBK1
28769055	Tombacz et al. 2017	WES Study	TTC28
28769055	Tombacz et al. 2017	WES Study	TTC34
25178164	Sokolowski et al. 2015	Review Article	TUBA1A
22059935; 28809398	Galfalvy et al. 2013; Niculescu et al. 2017	GWAS study not in Catalog; Gene Expression	TUBGCP3
28769055	Tombacz et al. 2017	WES Study	TXLNA
28872639	Flory et al. 2017	Gene Expression	UBC
28769055	Tombacz et al. 2017	WES Study	UBE2E3
23958961	Le-Niculescu et al. 2013	Gene Expression	UCHL5
28809398	Niculescu et al. 2017	Gene Expression	UQCC1
28769055	Tombacz et al. 2017	WES Study	VAC14

21081163; 28809398	Fiori et al. 2012; Niculescu et al. 2017	Gene Expression; Gene Expression	VAMP3
27721799	Mirkovic et al. 2016	Review Article	VAMP4
29331709	Bozorgmehr et al. 2018	Review Article	VEGFA
25178164	Sokolowski et al. 2015	Review Article	VGF
25178164	Sokolowski et al. 2015	Review Article	VIM
28809398	Niculescu et al. 2017	Gene Expression	VIP
28809398	Niculescu et al. 2017	Gene Expression	VPREB3
23958961	Le-Niculescu et al. 2013	Gene Expression	VPS53
28809398	Niculescu et al. 2017	Gene Expression	VTA1
28809398	Niculescu et al. 2017	Gene Expression	WARS
28769055	Tombacz et al. 2017	WES Study	WDR12
29331709	Bozorgmehr et al. 2018	Review Article	WFS1
28872639	Flory et al. 2017	Gene Expression	WIF1
28809398	Niculescu et al. 2017	Gene Expression	WIPF3
28809398	Niculescu et al. 2017	Gene Expression	WNK1
28872639	Flory et al. 2017	Gene Expression	WNT1
28872639	Flory et al. 2017	Gene Expression	WNT3
28809398	Niculescu et al. 2017	Gene Expression	WWP2
28809398	Niculescu et al. 2017	Gene Expression	XRCC5
28769055	Tombacz et al. 2017	WES Study	YES1
29331709	Bozorgmehr et al. 2018	Review Article	YWHAE
28809398; 25178164	Niculescu et al. 2017; Sokolowski et al. 2015	Gene Expression; Review Article	YWHAH
28872639	Flory et al. 2017	Gene Expression	YWHAQ
25178164	Sokolowski et al. 2015	Review Article	YWHAZ
28769055	Tombacz et al. 2017	WES Study	ZBTB49
28769055	Tombacz et al. 2017	WES Study	ZCCHC2
28769055	Tombacz et al. 2017	WES Study	ZEB2
28769055	Tombacz et al. 2017	WES Study	ZFC3H1
25178164	Sokolowski et al. 2015	Review Article	ZFP36
28809398	Niculescu et al. 2017	Gene Expression	ZFYVE21
28809398	Niculescu et al. 2017	Gene Expression	ZNF302
28769055	Tombacz et al. 2017	WES Study	ZNF510
28809398	Niculescu et al. 2017	Gene Expression	ZNF565
28769055	Tombacz et al. 2017	WES Study	ZNF646
28769055	Tombacz et al. 2017	WES Study	ZNF718
28809398	Niculescu et al. 2017	Gene Expression	ZNF75D
25178164	Sokolowski et al. 2015	Review Article	ZNF804A
28769055	Tombacz et al. 2017	WES Study	ZSCAN1

Table S5. Case characteristics of follow-up Utah suicides with significant SNP findings. No cases were related (out to 15th degree) to any case in the high risk family responsible for SGS evidence for the region containing that gene.

**Gene, SNP, N suicide cases**	**% male**	**Avg. age at death (SD)**	**Method of death[a]**	**Relatedness among cases with minor allele**
SP110, rs181058279, 5 heterozygotes	100% (5/5)	33.20 (5.85)	20% gun (1/5)<br>60% other violent (3/5)<br>20% overdose (1/5)	one 14th degree relative pair
AGBL2, rs76215382, 61 heterozygotes, 2 homozygotes	**64.06% (41/64)[b]**	34.05 (16.44)[c]	50% gun (32/64)<br>37.5% other violent (24/64)<br>12.5% overdose (8/64)	23 relative pairs: two 5th, one 7th, one 8th, six 9th, four 10th, three 12th, four 13th, two 14th
SUCLA2, rs121908538, 9 heterozygotes	77.78% (7/9)	30.94 (12.88)	33.3% gun (3/9)<br>55.5% other violent (5/9)<br>11.1% overdose (1/9)	two pairs of 12th degree relatives
APH1B, rs745918508, 23 heterozygotes	73.91% (17/23)	37.91 (17.86)[c]	56.5% gun (13/23)<br>34.8% other violent (8/23)<br>8.7% overdose (2/23)	two 11th degree relative pairs; one 12th degree relative pair

[a] For comparison, in 4370 Utah suicides with DNA, rates of causes of death were 52.6% gun-related, 32.0% other violent methods, and 15.3% overdose.
[b] Significantly fewer males (41 of 64 cases) compared to the expected rate from all Utah suicides; chi-square (1 df) = 7.82, p=0.003.
[c] Distribution is significantly right-skewed.

Additional information: diagnostic characteristics in cases for each of the four Table S5 SNPs appear in four additional tables on the following pages. Cases from this follow-up sample of 4370 cases matched the initial 215 familial cases in the discovery sample well. Young age at death, increases in personality disorders, and increases in previous suicide attempts/suicidal ideation were therefore expected.

Gene:
AGBL2

Case id	sex	age	method	Depr	BD	Anx	SCZ	Subst	Psnlty	ADHD	Sui-attempt	Sum	Totals	N Total	Percent
29445	M	21	chest wound	0	0	0	0	0	0	0	1	1	depression	30	0.47
33276	F	17	gun	0	0	0	0	0	0	0	0	0	bipolar	7	0.11
36087	M	22	gun	1	0	0	0	1	0	0	0	2	anxiety	16	0.25
42945	M	17	hanging	0	0	0	0	0	0	0	0	0	psychosis/SCZ	2	0.03
46829	M	30	gun	0	0	0	0	0	0	0	1	1	subst. abuse	9	0.14
47912	M	15	gun	0	0	0	0	0	0	0	1	1	personality	9	0.14
95743	F	52	hanging	1	0	1	0	0	0	0	0	2	adhd	5	0.08
117222	F	18	hanging	0	0	0	0	0	0	0	0	0	attempt/ideation	30	0.47
76063	M	19	gun	1	0	0	0	0	0	0	1	2	2 or more dx	27	0.42
117227	F	44	overdose-opioids-antipsychotics	1	0	0	0	0	0	0	1	2	4 or more dx	10	0.16
95789	M	54	gun	1	0	1	0	0	1	0	0	3	no codes	20	0.31
95795	F	36	hanging	1	0	1	0	1	0	1	1	5			
117274	F	59	overdose-narcotics-hallucinogens	1	0	1	0	0	0	0	0	2	male	41/64	0.64
95972	F	54	gun	0	0	0	0	0	0	0	0	0	mean age		34.05
84749	M	20	gun	0	0	0	0	0	0	0	1	1	sd age		16.44
85475	M	41	gun	0	0	0	0	0	0	0	0	0			
85468	F	37	overdose-narcotics-opioids	1	0	0	0	0	0	0	1	2	method:gun	32	0.50
85649	M	20	gun	0	0	0	0	0	0	0	0	0	method:od (includes CO)	7	0.11
85816	M	62	gun	0	0	0	0	0	0	0	0	0	method:hanging	22	0.34
86803	M	22	gun	0	0	0	0	0	0	0	0	0	method:other	3	0.05
87337	M	20	hanging	0	0	0	0	0	0	1	0	1	(stab wound, jump, other trauma)		
87461	M	17	hanging	0	0	0	0	0	0	0	1	1			
87990	M	62	gun	0	0	0	0	0	0	0	0	0			
88785	M	35	hanging	0	0	0	0	0	0	0	1	1			
89185	F	17	hanging	0	0	0	0	0	0	0	0	0			
89434	M	26	gun	0	0	0	0	0	0	0	0	0			
90923	M	57	gun	1	0	1	0	1	0	0	1	4			
114023	M	43	gun	1	0	0	0	0	0	0	1	2			
91755	M	16	gun	0	0	0	0	0	0	0	0	0			
93534	F	17	jump from high place	1	1	1	0	0	1	0	1	5			
93966	M	22	overdose-narcotics-alcohol	0	0	0	0	0	0	0	1	1			
94810	M	57	hanging	1	1	0	0	0	1	1	1	5			
95370	M	28	hanging	1	0	0	0	0	0	0	0	1			

115003	F	21	gun	1	1	1	1	0	1	0	1	6
96568	M	28	hanging	0	0	0	0	0	0	0	0	0
97264	F	41	hanging	1	0	1	0	0	0	0	1	3
97870	F	34	hanging	1	1	1	0	0	1	0	1	5
98950	F	25	hanging	1	1	1	0	1	1	0	1	6
99143	F	29	hanging	1	0	0	0	0	1	0	1	3
117317	M	62	gun	0	0	0	0	1	0	0	0	1
100781	M	17	gun	0	0	0	0	0	0	1	0	1
101347	M	14	gun	0	0	0	0	0	0	0	1	1
101629	M	56	poisoning-CO	0	0	0	0	0	0	1	0	1
101811	F	56	gun	0	0	0	0	0	0	0	0	0
101918	M	31	hanging	0	0	0	0	0	0	0	0	0
102189	F	20	hanging	1	0	0	0	0	0	0	1	2
102686	M	82	gun	0	0	1	0	0	0	0	0	1
103224	M	60	gun	0	0	0	0	0	0	0	0	0
103353	M	32	hanging	0	0	0	0	0	0	0	0	0
103666	M	25	hanging	1	1	1	1	1	1	0	1	7
104587	M	51	gun	1	0	0	0	0	0	0	0	1
104714	F	40	hanging	1	0	1	0	1	0	0	1	4
105692	M	15	gun	1	0	0	0	0	0	0	1	2
105841	M	32	gun	1	1	1	0	1	1	0	1	6
117351	F	60	overdose-antidepressants	1	0	0	0	0	0	0	0	1
106770	F	32	poisoning-CO	1	0	0	0	0	0	0	1	2
117357	M	28	gun	0	0	0	0	0	0	0	0	0
109312	F	16	gun	0	0	0	0	0	0	0	0	0
109862	F	51	hanging	1	0	0	0	1	0	0	0	2
109956	F	31	gun	1	0	1	0	0	0	0	1	3
117366	M	18	gun	1	0	1	0	0	0	0	1	3
112061	M	35	gun	0	0	0	0	0	0	0	0	0
113906	M	23	hanging	1	0	0	0	0	0	0	1	2
114189	M	37	blunt force trauma	0	0	0	0	0	0	0	0	0

Gene:
APH1B

Case id	sex	age	method	Depr	BD	Anx	SCZ	Subst	Psnlty	ADHD	Sui-attempt	Sum
27629	F	17	overdose-stimulants-otherdrugs	1	0	0	0	0	0	0	1	2
32989	M	23	gun	1	1	0	0	0	0	0	0	2
38885	F	32	overdose-opioids-methadone	0	0	0	0	0	0	0	0	0
41970	M	16	hanging	1	0	0	0	0	0	0	0	1
46301	M	48	gun	0	0	0	0	0	0	0	0	0
78440	F	33	gun	1	0	0	0	0	1	0	1	3
95972	F	54	gun	0	0	0	0	0	0	0	0	0
84261	M	46	hanging	0	0	0	0	0	0	0	0	0
89530	M	71	gun	1	0	1	0	0	0	0	0	2
89759	M	46	hanging	0	1	0	0	1	1	0	1	4
89761	M	19	gun	0	0	0	0	0	0	0	0	0
90772	M	56	gun	0	0	0	0	0	0	0	0	0
93400	F	18	hanging	0	0	0	0	0	0	0	0	0
97591	M	30	hanging	1	0	0	0	0	0	1	1	3
117306	M	28	gun	1	0	0	0	0	0	0	0	1
100016	M	16	hanging	0	0	0	0	0	0	0	0	0
102510	M	18	hanging	0	0	0	0	0	0	0	0	0
103553	M	59	gun	1	0	1	0	0	0	0	1	3
105153	F	52	gun	0	0	0	0	0	0	0	0	0
105841	M	32	gun	1	1	1	0	1	1	0	1	6
108968	M	74	gun	0	0	0	0	1	0	0	0	1
112061	M	35	gun	0	0	0	0	0	0	1	0	1
113614	M	49	stab wound of chest	0	0	0	0	0	0	0	0	0

Totals	N_Total	Percent
depression	9	0.39
bipolar	3	0.13
anxiety	3	0.13
psychosis	0	0.00
subst. abuse	3	0.13
personality	3	0.13
ADHD	2	0.09
attempt/ideation	6	0.26
2 or more dx	8	0.35
4 or more dx	5	0.22
No codes	11	0.48
males	17	0.74
avg age		37.91
sd age		17.86
method:gun	13	0.57
method:od	2	0.09
method:hanging	7	0.30
method:other	1	0.04
(stab wound)		

**Gene: *SUCLA2***															
**Case id**	**sex**	**age**	**method**	**Depr**	**BD**	**Anx**	**SCZ**	**Subst**	**Psnlty**	**ADHD**	**Sui-attempt**	**Sum**	Totals	N_Total	Percent
38884	M	36	hanging	0	0	0	0	0	0	0	0	0	depression	2	0.22
40128	F	18	hanging	0	0	0	0	0	0	0	0	0	bipolar	1	0.11
42052	M	42	poisoning-cyanide	0	0	0	0	0	0	0	0	0	psychosis	0	0.00
89185	F	17	hanging	0	0	0	0	0	0	0	0	0	subst. abuse	1	0.11
90067	M	53	gun	1	1	1	0	0	0	0	1	4	personality	0	0.00
90767	M	41	hanging	0	0	0	0	1	0	0	0	1	attempt/ideation	3	0.33
97703	M	20	gun	0	0	0	0	0	0	0	1	1	2 or more	2	0.22
98236	M	31	gun	1	0	1	0	0	0	0	1	3	4 or more	1	0.11
104982	M	21	hanging	0	0	0	0	0	0	0	0	0	no codes	5	0.56
													males	7	0.78
													avg age		31.00
													sd age		12.83
													method:gun	3	0.33
													method:od	1	0.11
													method:hanging	5	0.56

**gene: *SP110***															
**Case id**	**sex**	**age**	**method**	**Depr**	**BD**	**Anx**	**SCZ**	**Subst**	**Psnlty**	**ADHD**	**Sui-attempt**	**Sum**	**Totals**	**N_Total**	**Percent**
31426	M	26	head injury; blunt force trauma	1	0	0	0	0	0	0	1	2	**depression**	**2**	**0.40**
85481	M	39	gun	0	0	0	0	0	0	0	0	0	**bipolar**	**0**	**0.00**
87902	M	29	hanging	1	0	0	0	0	1	1	0	3	**anxiety**	**0**	**0.00**
95049	M	33	CO poisoning	0	0	0	0	0	0	0	0	0	**psychosis/SCZ**	**0**	**0.00**
102878	M	39	hanging	0	0	0	0	0	0	0	0	0	**subst.abuse**	**0**	**0.00**
													**personality**	**1**	**0.20**
													**ADHD**	**1**	**0.20**
													**attempt/ideation**	**1**	**0.20**
													**2 or more**	**2**	**0.40**
													**no codes**	**3**	**0.60**
													**Males**	**5**	**1.00**
													**avg age**		**33.20**
													**sd age**		**5.85**
													**method:gun**	**1**	**0.20**
													**method:od**	**1**	**0.20**
													**method:hanging**	**2**	**0.40**
													**method:other**	**1**	**0.20**
													(head injury)		

References appearing in the Supplementary material

1 Schosser A, Butler AW, Ising M, Perroud N, Uher R, Ng MY *et al.* Genomewide association scan of suicidal thoughts and behaviour in major depression. *PLoS One* 2011; **6**. doi:10.1371/journal.pone.0020690.

2 Fullerton JM, Willis-Owen SAG, Yalcin B, Shifman S, Copley RR, Miller SR *et al.* Human-Mouse Quantitative Trait Locus Concordance and the Dissection of a Human Neuroticism Locus. *Biol Psychiatry* 2008; **63**: 874–883.

3 Xie Z, Chan EC, Druey KM. R4 Regulator of G Protein Signaling (RGS) Proteins in Inflammation and Immunity. *AAPS J* 2016; **18**: 294–304.

4 Galfalvy H, Haghighi F, Hodgkinson C, Goldman D, Oquendo MA, Burke A *et al.* A genome-wide association study of suicidal behavior. *Am J Med Genet Part B Neuropsychiatr Genet* 2015; **168**: 557–563.

5 Chen X, Chen X, Xu Y, Yang W, Wu N, Ye H *et al.* Association of six CpG-SNPs in the inflammation-related genes with coronary heart disease. *Hum Genomics* 2016; **10**. doi:10.1186/s40246-016-0067-1.

6 Casado PL, Aguiar DP, Costa LC, Fonseca MA, Vieira TCS, Alvim-Pereira CCK *et al.* Different contribution of BRINP3 gene in chronic periodontitis and peri-implantitis: A cross-sectional study. *BMC Oral Health* 2015; **15**. doi:10.1186/s12903-015-0018-6.

7 Smith PJ, Levine AP, Dunne J, Guilhamon P, Turmaine M, Sewell GW *et al.* Mucosal transcriptomics implicates under expression of BRINP3 in the pathogenesis of ulcerative colitis. *Inflamm Bowel Dis* 2014; **20**: 1802–1812.

8 Wang J, Campbell IL, Zhang H. Systemic interferon-α regulates interferon-stimulated genes in the central nervous system. *Mol Psychiatry* 2008; **13**: 293–301.

9 Hoyo-Becerra C, Huebener A, Trippler M, Lutterbeck M, Liu ZJ, Truebner K *et al.* Concomitant interferon alpha stimulation and TLR3 activation induces neuronal expression of depression-related genes that are elevated in the brain of suicidal persons. *PLoS One* 2013; **8**. doi:10.1371/journal.pone.0083149.

10 Liu Y, Ramot Y, Torrelo A, Paller AS, Si N, Babay S *et al.* Mutations in proteasome subunit β type 8 cause chronic atypical neutrophilic dermatosis with lipodystrophy and elevated temperature with evidence of genetic and phenotypic heterogeneity. *Arthritis Rheum* 2012; **64**: 895–907.

11 Chmielewski S, Piaszyk-Borychowska A, Wesoly J, Bluyssen HAR. STAT1 and IRF8 in Vascular Inflammation and Cardiovascular Disease: Diagnostic and Therapeutic Potential. *Int Rev Immunol* 2016; **35**: 434–454.

12 Chen Z, Guo Z, Ma J, Liu F, Gao C, Liu S *et al.* STAT1 single nucleotide polymorphisms and susceptibility to immune thrombocytopenia. *Autoimmunity* 2015; **48**: 305–312.

13 Zimmerman O, Rosen LB, Swamydas M, Ferre EMN, Natarajan M, van de Veerdonk F *et al.* Autoimmune regulator deficiency results in a decrease in STAT1 levels in human monocytes. *Front Immunol* 2017; **8**. doi:10.3389/fimmu.2017.00820.

14 Sequeira A, Mamdani F, Ernst C, Vawter MP, Bunney WE, Lebel V *et al.* Global brain gene expression analysis links Glutamatergic and GABAergic alterations to suicide and major depression. *PLoS One* 2009; **4**. doi:10.1371/journal.pone.0006585.

15 Xu FF, Huang Y, Wang XQ, Qiu YH, Peng YP. Modulation of immune function by glutamatergic neurons in the cerebellar interposed nucleus via hypothalamic and sympathetic pathways. *Brain Behav Immun* 2014; **38**: 263–271.

16 Mitjans M, Arias B, Jiménez E, Goikolea JM, Sáiz PA, García-Portilla MP *et al.* Exploring Genetic Variability at PI, GSK3, HPA, and Glutamatergic Pathways in Lithium Response: Association with IMPA2, INPP1, and GSK3B Genes. *J Clin Psychopharmacol* 2015; **35**: 600–604.

17 Lindén M, Ramírez Sepúlveda JI, James T, Thorlacius GE, Brauner S, Gómez-Cabrero D *et al.* Sex influences eQTL effects of SLE and Sjögren's syndrome-associated genetic polymorphisms. *Biol Sex Differ* 2017; **8**. doi:10.1186/s13293-017-0153-7.

18 Niculescu AB, Le-Niculescu H, Levey DF, Phalen PL, Dainton HL, Roseberry K *et al.* Precision medicine for suicidality: From universality to subtypes and personalization. *Mol Psychiatry* 2017; **22**: 1250–1273.

19 Menke A, Domschke K, Czamara D, Klengel T, Hennings J, Lucae S *et al.* Genome-wide association study of antidepressant treatment-emergent suicidal ideation. *Neuropsychopharmacology* 2012; **37**: 797–807.

20 Harraz MM, Snyder SH. Antidepressant Actions of Ketamine Mediated by the Mechanistic Target of Rapamycin, Nitric Oxide, and Rheb. Neurotherapeutics. 2017; **14**: 728–733.

21 Li K, Zhang Y, Liang KY, Xu S, Zhou XJ, Tan K *et al.* Rheb1 deletion in myeloid cells aggravates OVA-induced allergic

inflammation in mice. *Sci Rep* 2017; **7**. doi:10.1038/srep42655.
22 Tombácz D, Maróti Z, Kalmár T, Csabai Z, Balázs Z, Takahashi S *et al.* High-Coverage Whole-Exome Sequencing Identifies Candidate Genes for Suicide in Victims with Major Depressive Disorder. *Sci Rep* 2017; **7**: 1–11.
23 Shen C, Liu L, Jiang Z, Zheng X, Meng L, Yin X *et al.* Four genetic variants interact to confer susceptibility to atopic dermatitis in Chinese Han population. *Mol Genet Genomics* 2015; **290**: 1493–8.
24 Gao L, Emond MJ, Louie T, Cheadle C, Berger AE, Rafaels N *et al.* Identification of Rare Variants in ATP8B4 as a Risk Factor for Systemic Sclerosis by Whole-Exome Sequencing. *Arthritis Rheumatol* 2016; **68**: 191–200.
25 Rujescu D, Giegling I, Mandelli L, Schneider B, Hartmann AM, Schnabel A *et al.* NOS-I and -III gene variants are differentially associated with facets of suicidal behavior and aggression-related traits. *Am J Med Genet Part B Neuropsychiatr Genet* 2008; **147**: 42–48.
26 Kanwar JR, Kanwar RK, Burrow H, Baratchi S. Recent advances on the roles of NO in cancer and chronic inflammatory disorders. *Curr Med Chem* 2009; **16**: 2373–94.
27 Choi K, Le T, Xing G, Johnson LR, Ursano RJ. Analysis of Kinase Gene Expression in the Frontal Cortex of Suicide Victims: Implications of Fear and Stress†. *Front Behav Neurosci* 2011; **5**. doi:10.3389/fnbeh.2011.00046.
28 Bavley CC, Fischer DK, Rizzo BK, Rajadhyaksha AM. Cav1.2 channels mediate persistent chronic stress-induced behavioral deficits that are associated with prefrontal cortex activation of the p25/Cdk5-glucocorticoid receptor pathway. *Neurobiol Stress* 2017; **7**: 27–37.
29 Song H, Kim W, Choi JH, Kim SH, Lee D, Park CH *et al.* Stress-induced nuclear translocation of CDK5 suppresses neuronal death by downregulating ERK activation via VRK3 phosphorylation. *Sci Rep* 2016; **6**. doi:10.1038/srep28634.
30 Quintanilla RA, Orellana DI, González-Billault C, Maccioni RB. Interleukin-6 induces Alzheimer-type phosphorylation of tau protein by deregulating the cdk5/p35 pathway. *Exp Cell Res* 2004; **295**: 245–257.
31 Kékesi KA, Juhász G, Simor A, Gulyássy P, Szego ÉM, Hunyadi-Gulyás É *et al.* Altered Functional Protein Networks in the Prefrontal Cortex and Amygdala of Victims of Suicide. *PLoS One* 2012; **7**. doi:10.1371/journal.pone.0050532.
32 Martins-De-Souza D, Gattaz WF, Schmitt A, Rewerts C, Maccarrone G, Dias-Neto E *et al.* Prefrontal cortex shotgun proteome analysis reveals altered calcium homeostasis and immune system imbalance in schizophrenia. *Eur Arch Psychiatry Clin Neurosci* 2009; **259**: 151–163.
33 Lucotte B, Tajhizi M, Alkhatib D, Samuelsson EB, Wiehager B, Schedin-Weiss S *et al.* Stress Conditions Increase Vimentin Cleavage by Omi/HtrA2 Protease in Human Primary Neurons and Differentiated Neuroblastoma Cells. *Mol Neurobiol* 2015; **52**: 1077–1092.
34 Wegner N, Lundberg K, Kinloch A, Fisher B, Malmström V, Feldmann M *et al.* Autoimmunity to specific citrullinated proteins gives the first clues to the etiology of rheumatoid arthritis. Immunol. Rev. 2010; **233**: 34–54.
35 Folmsbee SS, Gottardi CJ. Cardiomyocytes of the heart and pulmonary veins: Novel contributors to asthma? Am. J. Respir. Cell Mol. Biol. 2017; **57**: 512–518.
36 Flory JD, Donohue D, Muhie S, Yang R, Miller SA, Hammamieh R *et al.* Gene expression associated with suicide attempts in US veterans. *Transl Psychiatry* 2017; **7**. doi:10.1038/tp.2017.179.
37 Watanabe Y, Fujiyama A, Ichiba Y, Hattori M, Yada T, Sakaki Y *et al.* Chromosome-wide assessment of replication timing for human chromosomes 11q and 21q: disease-related genes in timing-switch regions. *Hum Mol Genet* 2002; **11**: 13–21.
38 Kerns D, Vong GS, Barley K, Dracheva S, Katsel P, Casaccia P *et al.* Gene expression abnormalities and oligodendrocyte deficits in the internal capsule in schizophrenia. *Schizophr Res* 2010; **120**: 150–158.
39 Riester A, Spyroglou A, Neufeld-Cohen A, Chen A, Beuschlein F. Urocortin-dependent effects on adrenal morphology, growth, and expression of steroidogenic enzymes in vivo. *J Mol Endocrinol* 2012; **48**: 159–167.
40 Katoh M, Katoh M. STAT3-induced WNT5A signaling loop in embryonic stem cells, adult normal tissues, chronic persistent inflammation, rheumatoid arthritis and cancer (Review). Int. J. Mol. Med. 2007; **19**: 273–278.
41 Bani-Fatemi A, Howe AS, Matmari M, Koga A, Zai C, Strauss J *et al.* Interaction between methylation and CpG single-nucleotide polymorphisms in the HTR2A gene: Association analysis with suicide attempt in schizophrenia. *Neuropsychobiology* 2016; **73**: 10–15.
42 Höfer P, Schosser A, Calati R, Serretti A, Massat I, Kocabas NA *et al.* The impact of serotonin receptor 1A and 2A gene polymorphisms and interactions on suicide attempt and suicide risk in depressed patients with insufficient

response to treatment - A European multicentre study. *Int Clin Psychopharmacol* 2016; **31**: 1–7.
43 McMahon FJ, Buervenich S, Charney D, Lipsky R, Rush AJ, Wilson AF *et al.* Variation in the Gene Encoding the Serotonin 2A Receptor Is Associated with Outcome of Antidepressant Treatment. *Am J Hum Genet* 2006; **78**: 804–814.
44 Idzko M, Panther E, Stratz C, Müller T, Bayer H, Zissel G *et al.* The serotoninergic receptors of human dendritic cells: identification and coupling to cytokine release. *J Immunol* 2004; **172**: 6011–9.
45 Takata Y, Hamada D, Miyatake K, Nakano S, Shinomiya F, Scafe CR *et al.* Genetic association between the PRKCH gene encoding protein kinase Ceta isozyme and rheumatoid arthritis in the Japanese population. *Arthritis Rheum* 2007; **56**: 30–42.
46 Starnawska A, Demontis D, McQuillin A, O'Brien NL, Staunstrup NH, Mors O *et al.* Hypomethylation of FAM63B in bipolar disorder patients. *Clin Epigenetics* 2016; **8**: 1–6.
47 Goes FS, Mcgrath J, Avramopoulos D, Wolyniec P, Pirooznia M, Ruczinski I *et al.* Genome-wide association study of schizophrenia in Ashkenazi Jews. *Am J Med Genet Part B Neuropsychiatr Genet* 2015; **168**: 649–659.
48 Hesselbrock V, Dick D, Hesselbrock M, Foroud T, Schuckit M, Edenberg H *et al.* The Search for Genetic Risk Factors Associated With Suicidal Behavior. *Alcohol Clin Exp Res* 2004; **28**: 70S–76S.
49 Zubenko GS, Maher BS, Hughes HB, Zubenko WN, Scott Stiffler J, Marazita ML. Genome-wide linkage survey for genetic loci that affect the risk of suicide attempts in families with recurrent, early-onset, major depression. *Am J Med Genet* 2004; **129B**: 47–54.
50 Willour VL, Zandi PP, Badner JA, Steele J, Miao K, Lopez V *et al.* Attempted Suicide in Bipolar Disorder Pedigrees: Evidence for Linkage to 2p12. *Biol Psychiatry* 2007; **61**: 725–727.
51 Butler AW, Breen G, Tozzi F, Craddock N, Gill M, Korszun A *et al.* A genomewide linkage study on suicidality in major depressive disorder confirms evidence for linkage to 2p12. *Am J Med Genet Part B Neuropsychiatr Genet* 2010; **153**: 1465–1473.
52 Cheng R, Juo SH, Loth JE, Nee J, Iossifov I, Blumenthal R *et al.* Genome-wide linkage scan in a large bipolar disorder sample from the National Institute of Mental Health genetics initiative suggests putative loci for bipolar disorder, psychosis, suicide, and panic disorder. *Mol Psychiatry* 2006; **11**: 252–260.
