# Supplementary material for: Genome-wide significant regions in 43 Utah high-risk families implicate multiple genes involved in risk for completed suicide
Source: Mol Psychiatry. 2018 Oct 23;25(11):3077–90. doi: 10.1038/s41380-018-0282-3 (PMC6478563; doi:10.1038/s41380-018-0282-3)

Supplementary Materials

Figure 1. Flow diagram of the study.

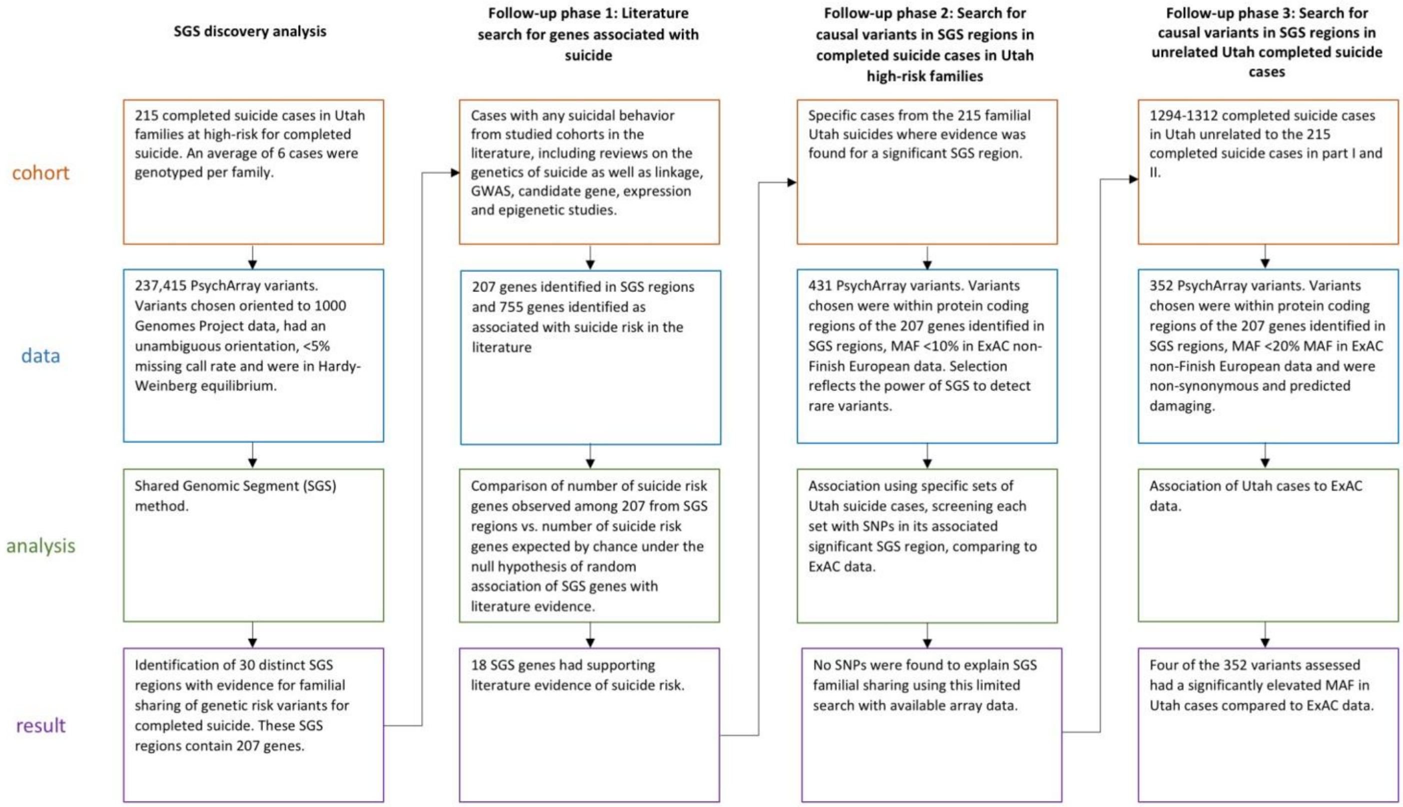

Figure S2. Simplified hypothetical data defining familial shared genomic segments.

**Family 1**	**SNP 1**	**SNP 2**	**SNP 3**	**SNP 4**	***Risk Variant***	**SNP 5**	**SNP 6**	**SNP 7**	***Risk Variant***	**SNP 8**	**SNP 9**
**Case 1**	1/ 2	1/ 2	1/ 1	1/ 2	*1/ 2*	1/ 2	1/ 2	1/ 2	*1/ 1*	1/ 2	2/ 2
**Case 2**	2/ 2	1/ 2	1/ 2	1/ 2	*1/ 2*	1/ 1	1/ 1	2/ 2	*1/ 1*	2/ 2	1/ 2
**Case 3**	1/ 2	1/ 1	1/ 2	2/ 2	*1/ 2*	1/ 2	1/ 2	1/ 2	*1/ 1*	1/ 1	1/ 1
**Case 4**	2/ 2	1/ 1	1/ 2	1/ 1	*1/ 1*	2/ 2	1/ 2	1/ 1	*1/ 1*	2/ 2	1/ 1
**Case 5**	2/ 2	1/ 2	1/ 1	1/ 2	*2/ 2*	1/ 2	1/ 1	2/ 2	*1/ 1*	1/ 2	2/ 2
**Family 2**											
**Case 1**	1/1	2/ 2	2/ 2	1/ 2	*1/ 1*	1 /2	1/ 2	1/ 2	*1/ 2*	1/ 1	1 /2
**Case 2**	2/2	1/ 2	2/ 2	2/ 2	*1/ 1*	1/ 1	1/ 2	2/ 2	*1/ 2*	1/ 2	1/ 1
**Case 3**	1/1	1/ 1	1/ 2	1/ 2	*1/ 1*	1/ 2	1/ 1	2/ 2	*1/ 2*	1/ 2	1/ 1
**Case 4**	1/2	2/ 2	1/ 1	1/ 1	*1/ 1*	2/ 2	2/ 2	1/ 1	*1/ 1*	2/ 2	1/ 2

The figure depicts highly simplified genotypes in a region with evidence for sharing across four cases in one high risk family and three cases in a second high risk family. For SGS significance, sharing of contiguous alleles at each polymorphic SNP must be rare in reference data from the 1000 Genomes Project. Statistically significant, unusually long familial sharing segments must also occur above and beyond chance sharing due to linkage disequilibrium. Significant SGS results are based on hundreds of thousands of simulations of each family. These simulations create a distribution of possible lengths of sharing which is independent of case status. The distribution of simulated results allows the derivation of significance thresholds for observed sharing. These thresholds account for testing across the genome and across possible subsets of case sharing within each family; they are additionally specific to the overall family size and dispersion of cases within each family.

Significant SGS regions are likely to harbor rare risk variants. Assumptions regarding risk variants are:

1) Risk variants within a shared region may not be the same across two families that have evidence for the region, as depicted in this hypothetical figure.
2) It is possible that a risk variant does not exist on the genotyping array used to define shared regions, and may therefore be unknown.
3) Shared regions may contain no genes or many genes, and while a risk variant may occur in a gene coding region, it may also be regulatory without clear annotation that describes functional consequences—in this case, it may be difficult to determine even with whole genome sequence data.
4) A risk variant likely interacts with other risk variants at other genomic locations, with aspects of polygenic risk, and/or with environmental risks.
5) Risk may actually be a more complex event than a single base pair variant, such as an insertion, deletion, or a haplotype of multiple variants.

Figure S3. Example of suicide cases with complex family relationships to more than one founding couple.

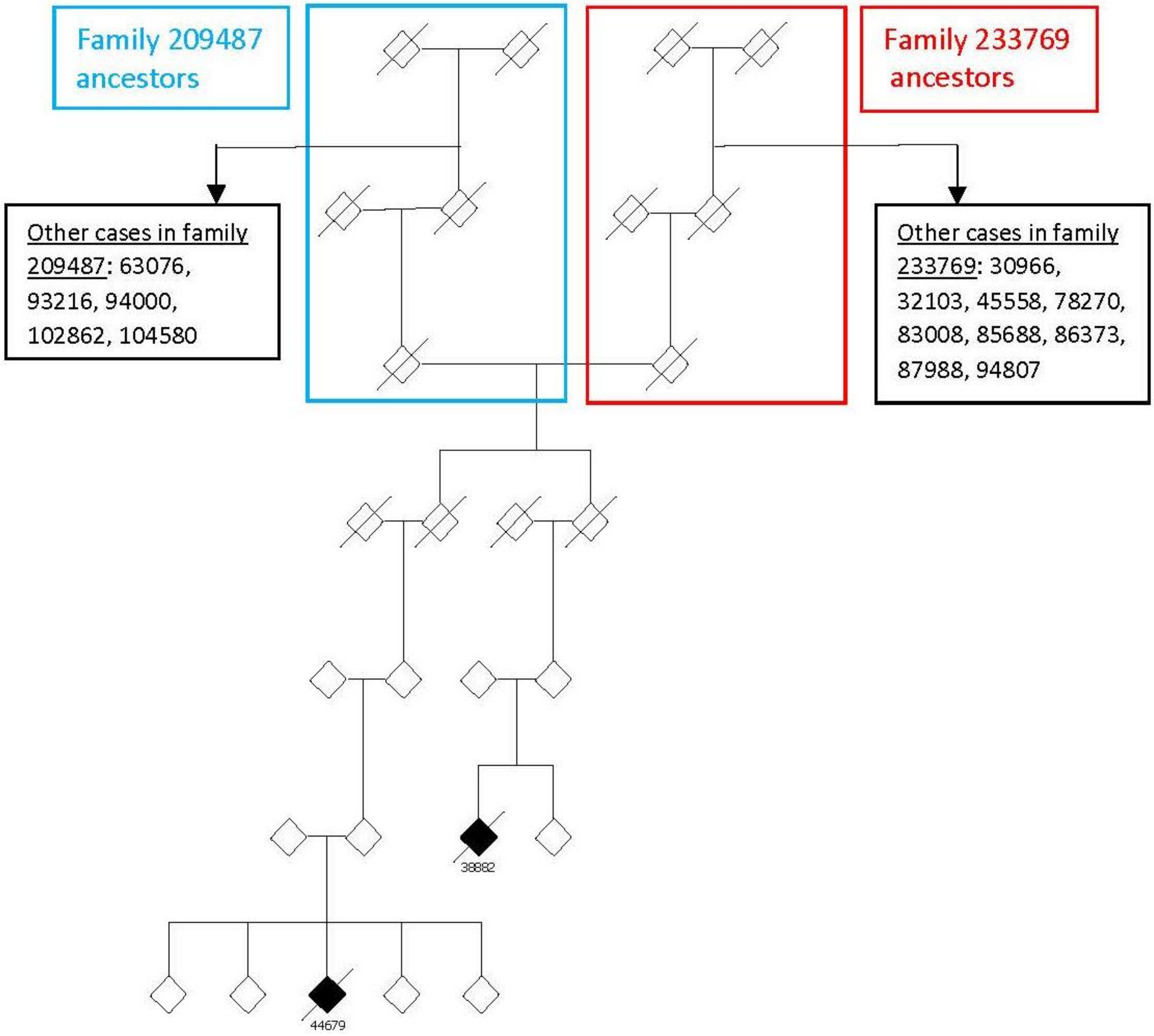

Figure S4a. Extended family structures linking cases used for SGS analyses in the 10 families with genome-wide significant results within a single family. All analyzed cases are represented by a numeric ID. Only suicide cases in the line of descent to analyzed cases are shown. Gender is disguised and sibship order is randomized in order to protect the privacy of family members. NOTE: Suicide cases are not as evident in upper generations because suicide status from death certificates is only available back to 1904.

Family 709

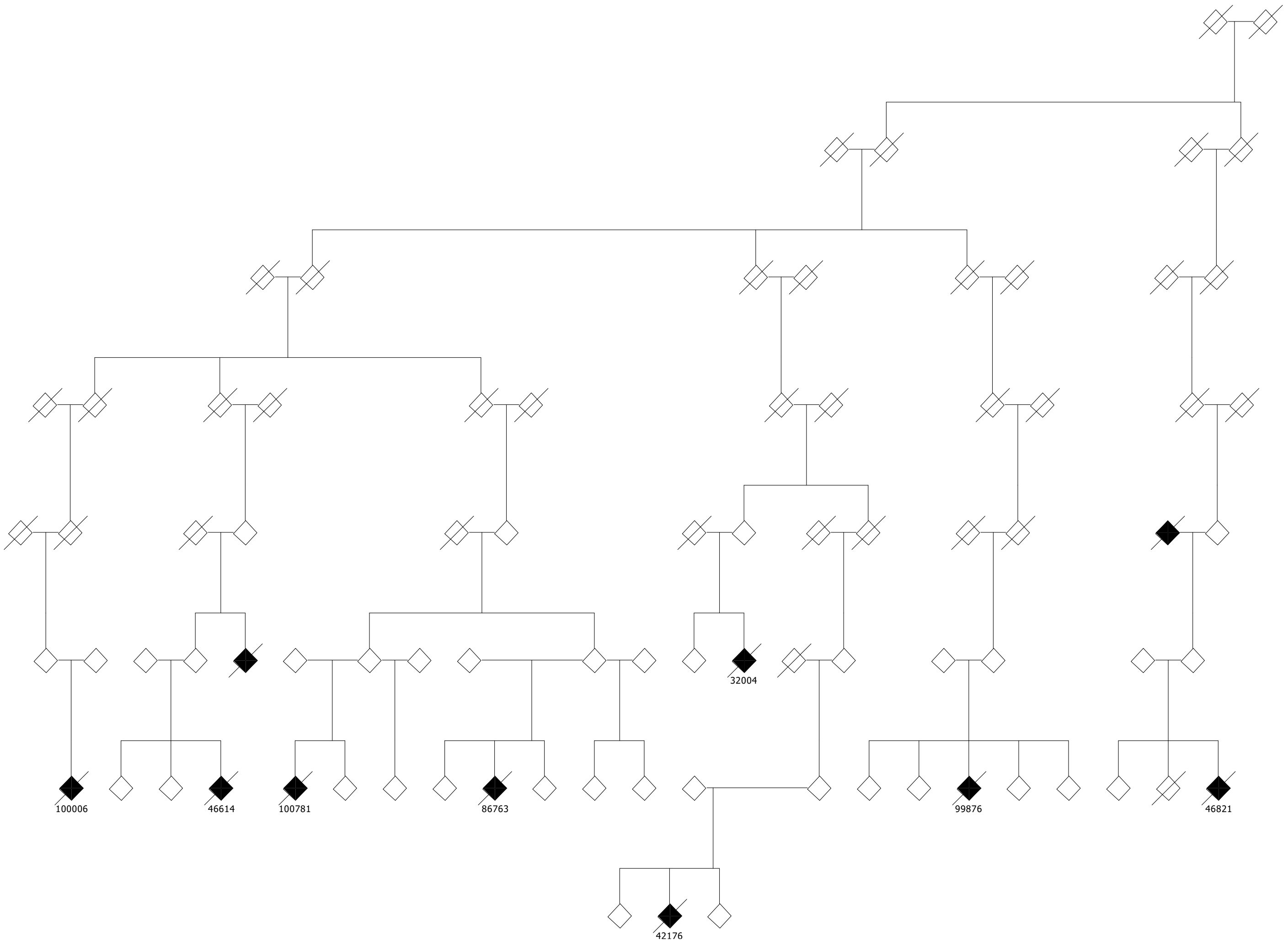

Family 60205

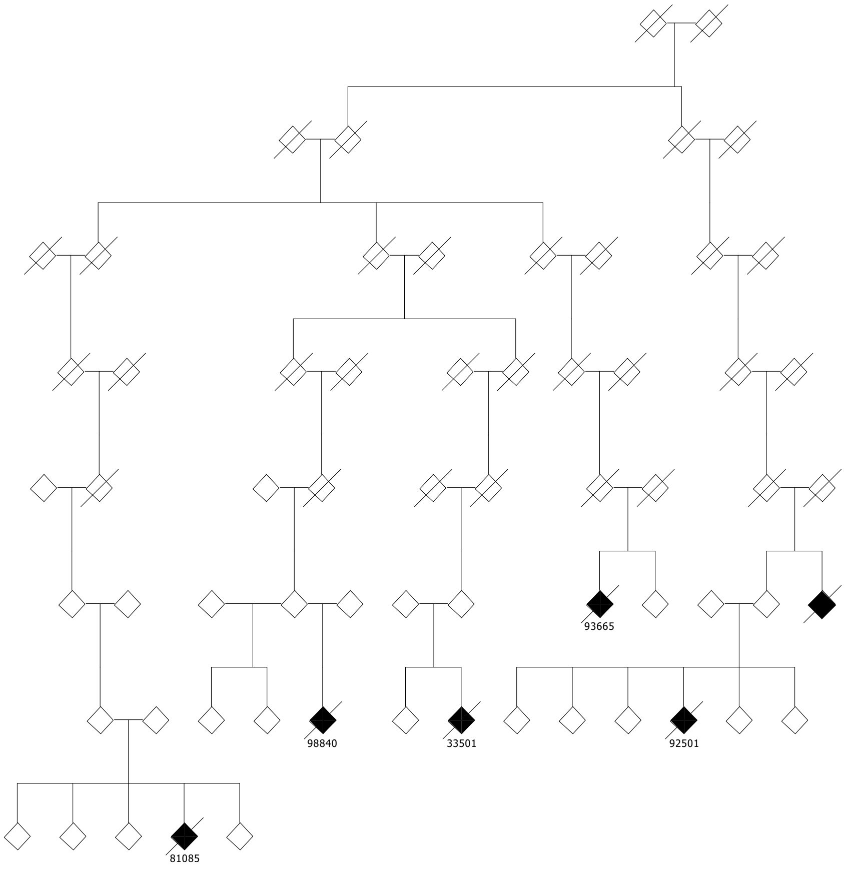

Family 66494

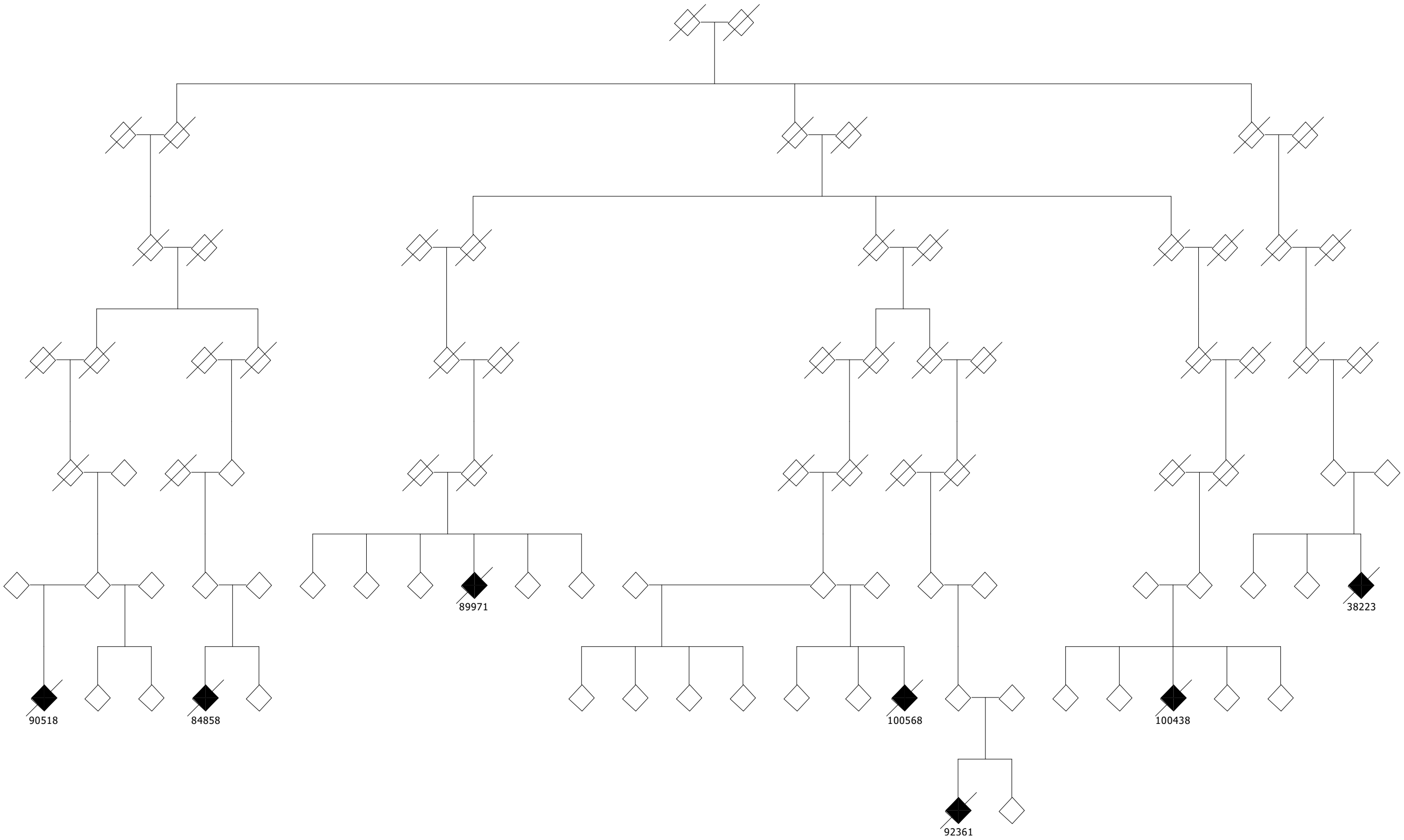

Family 209487

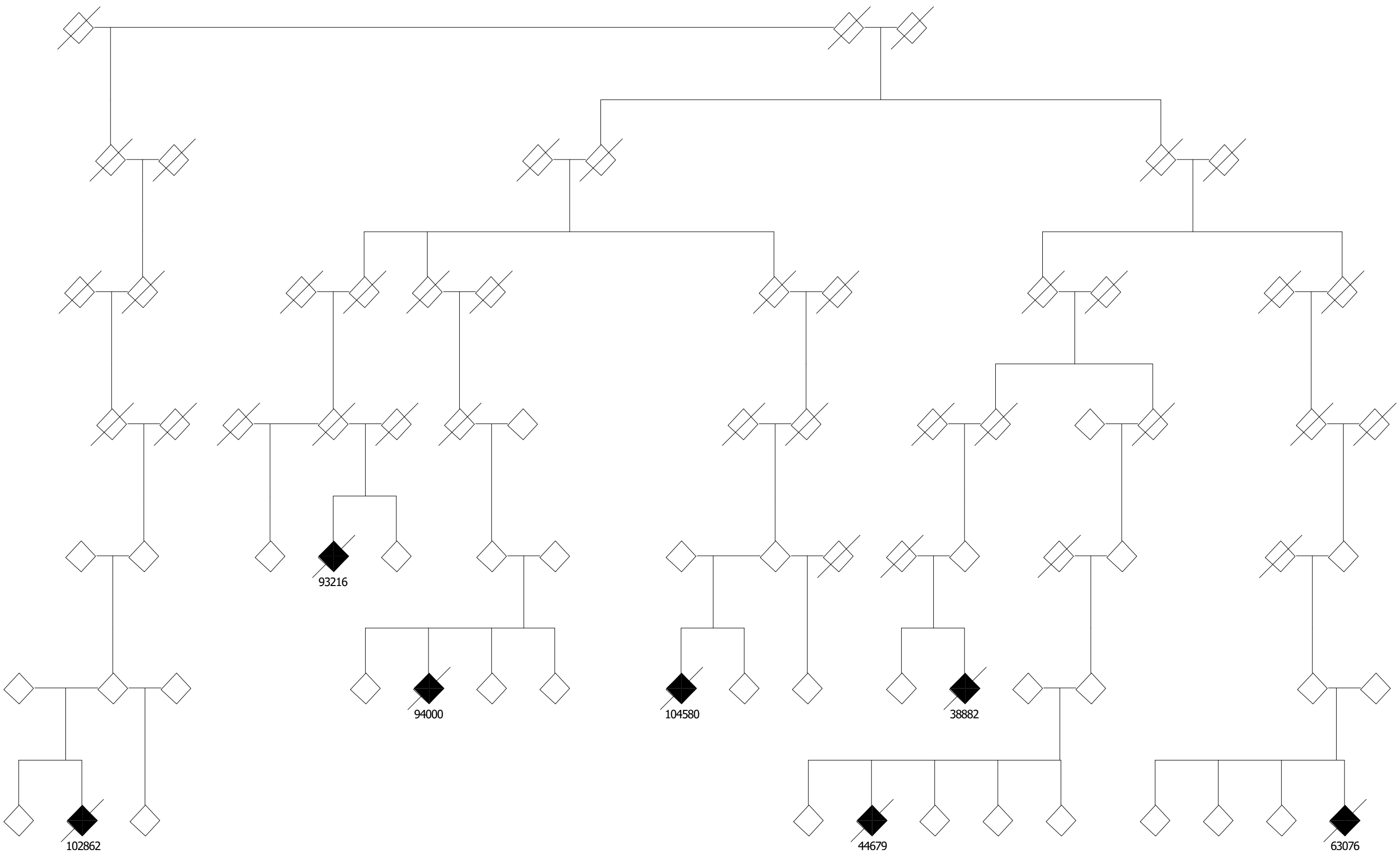

Family 233769

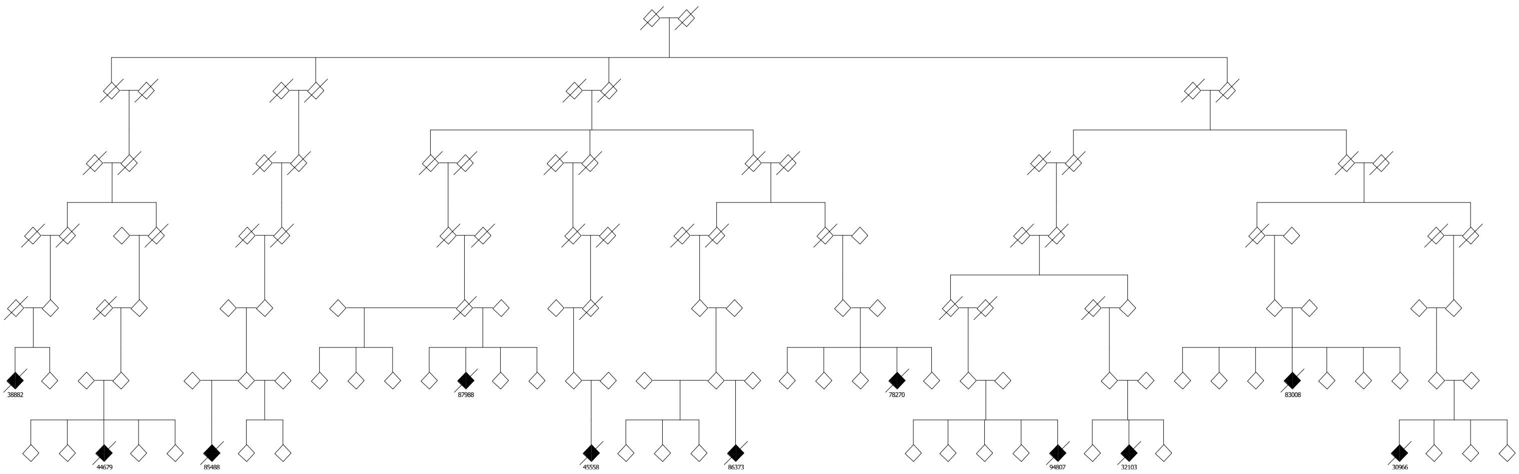

Family 540775

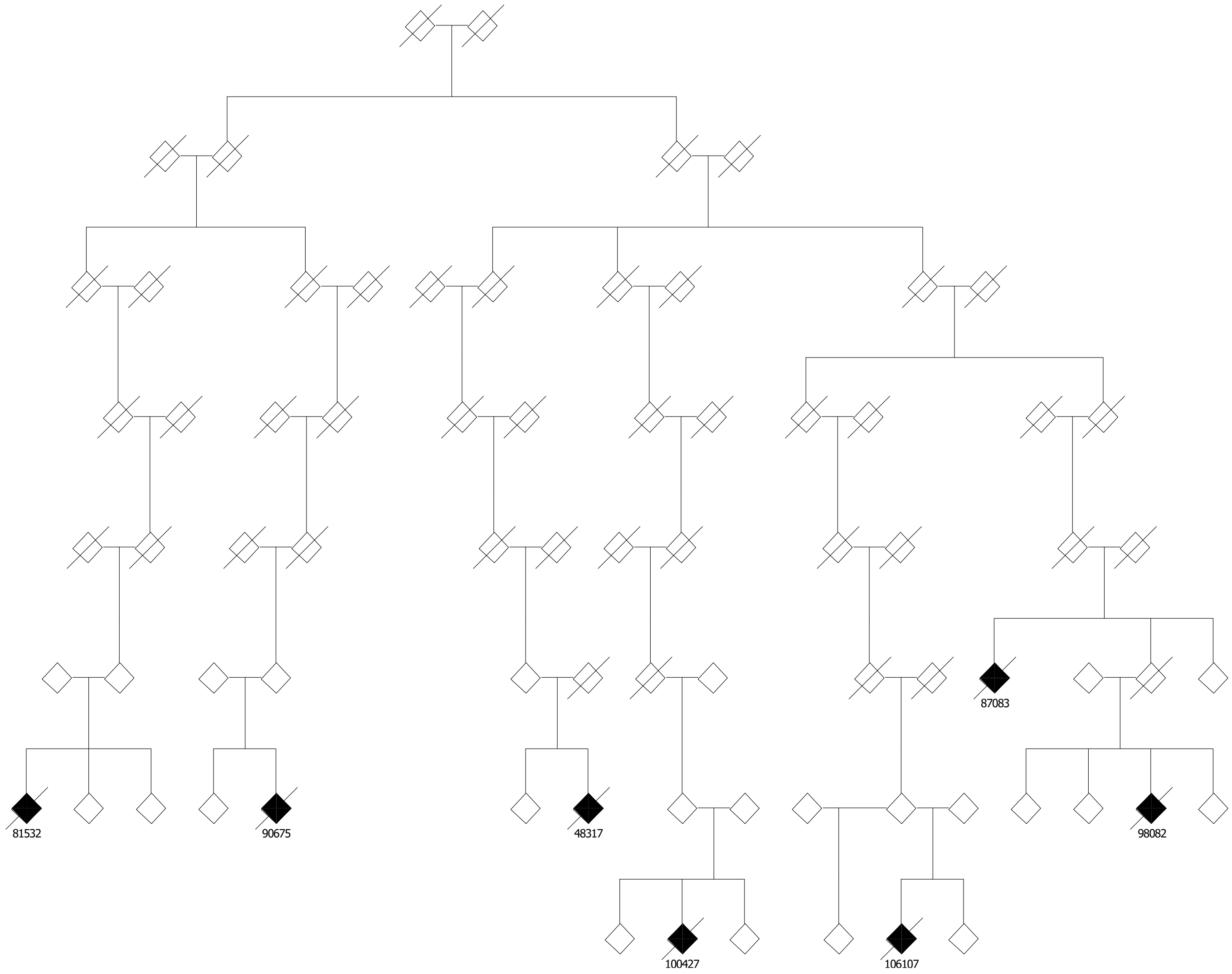

Family 553615

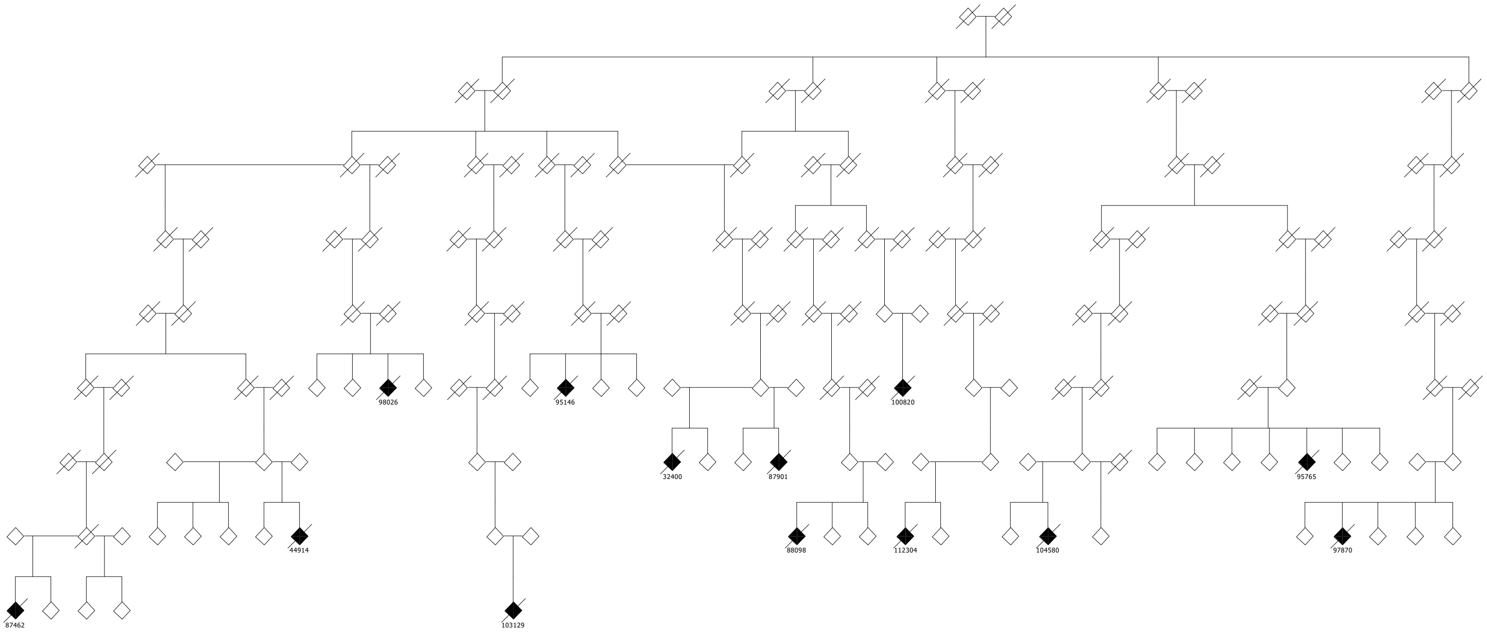

Family 601627

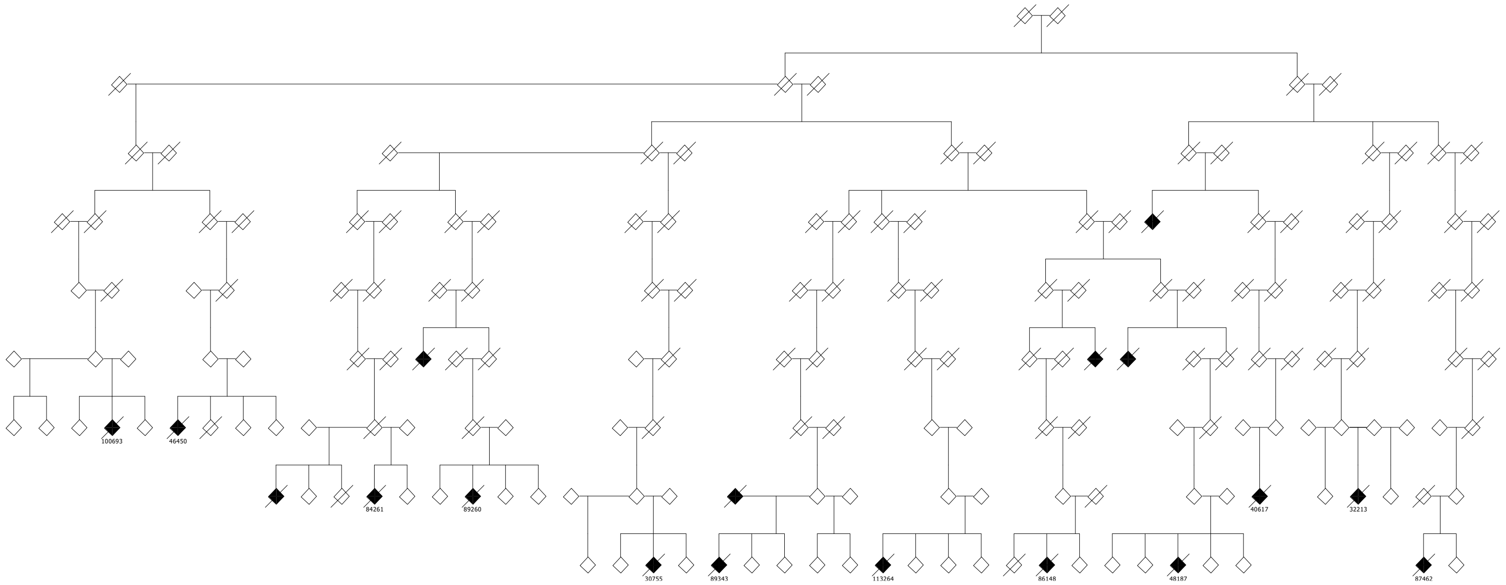

Family 603481

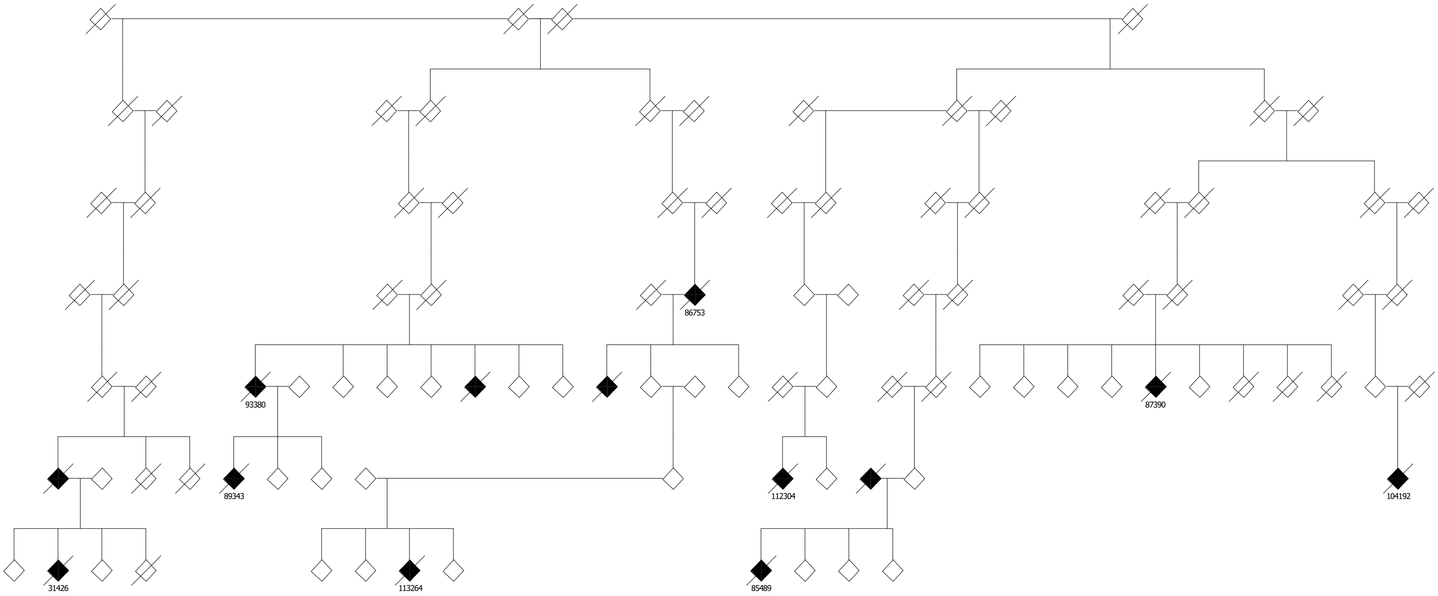

Family 807334

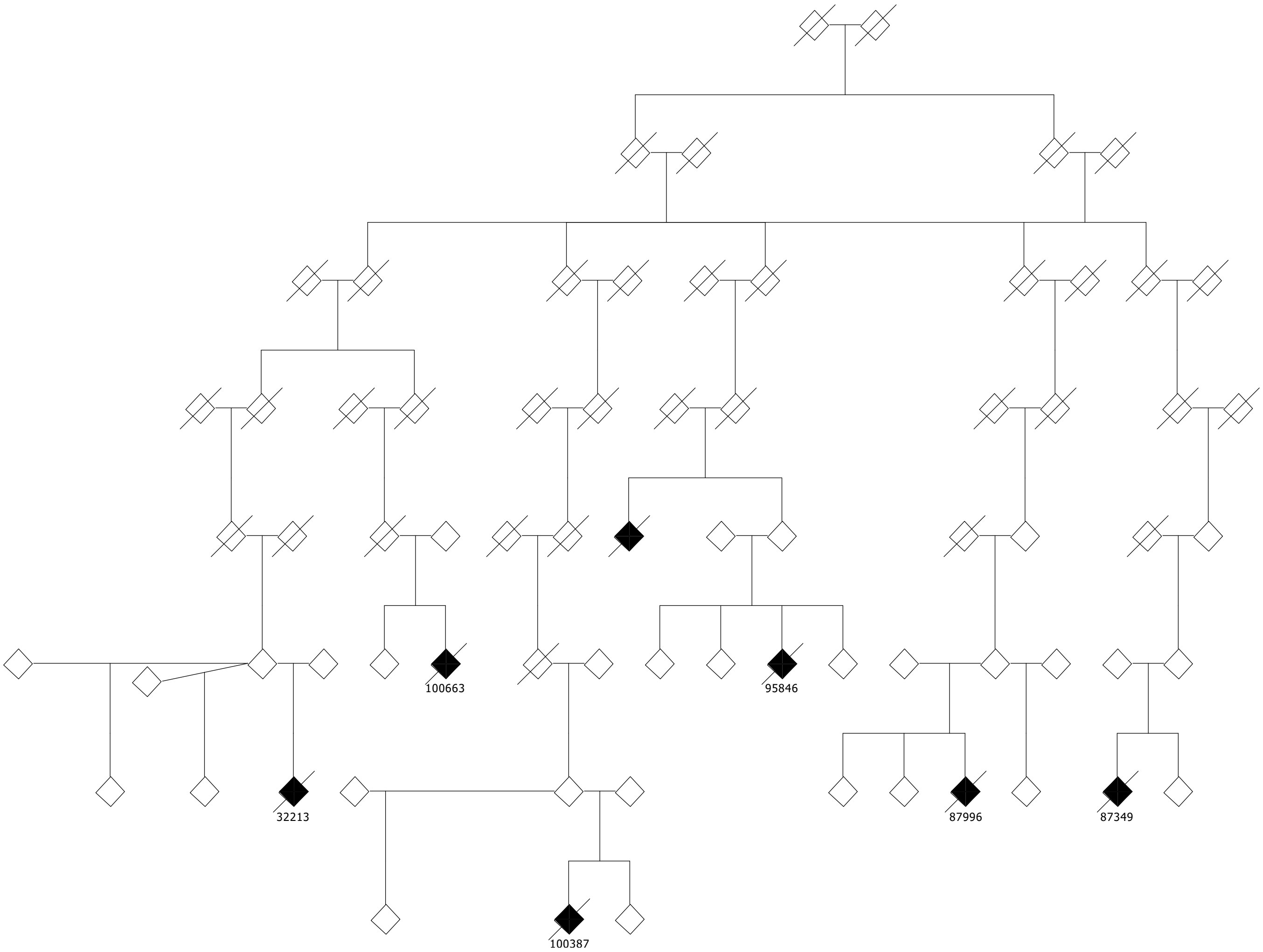

Figure S4b. Detail of extended family structures in the families with SGS evidence of shared regions overlapping across more than one family. All regions shared across two families are presented in drawings i through xiv. The region on chromosome five, shared across three families, is presented in drawing xv.

i. Six cases in Family 709 share a region on chromosome 1p34.2 with five cases in family 8556 (the sharing cases in the two families are circled).

Family 709

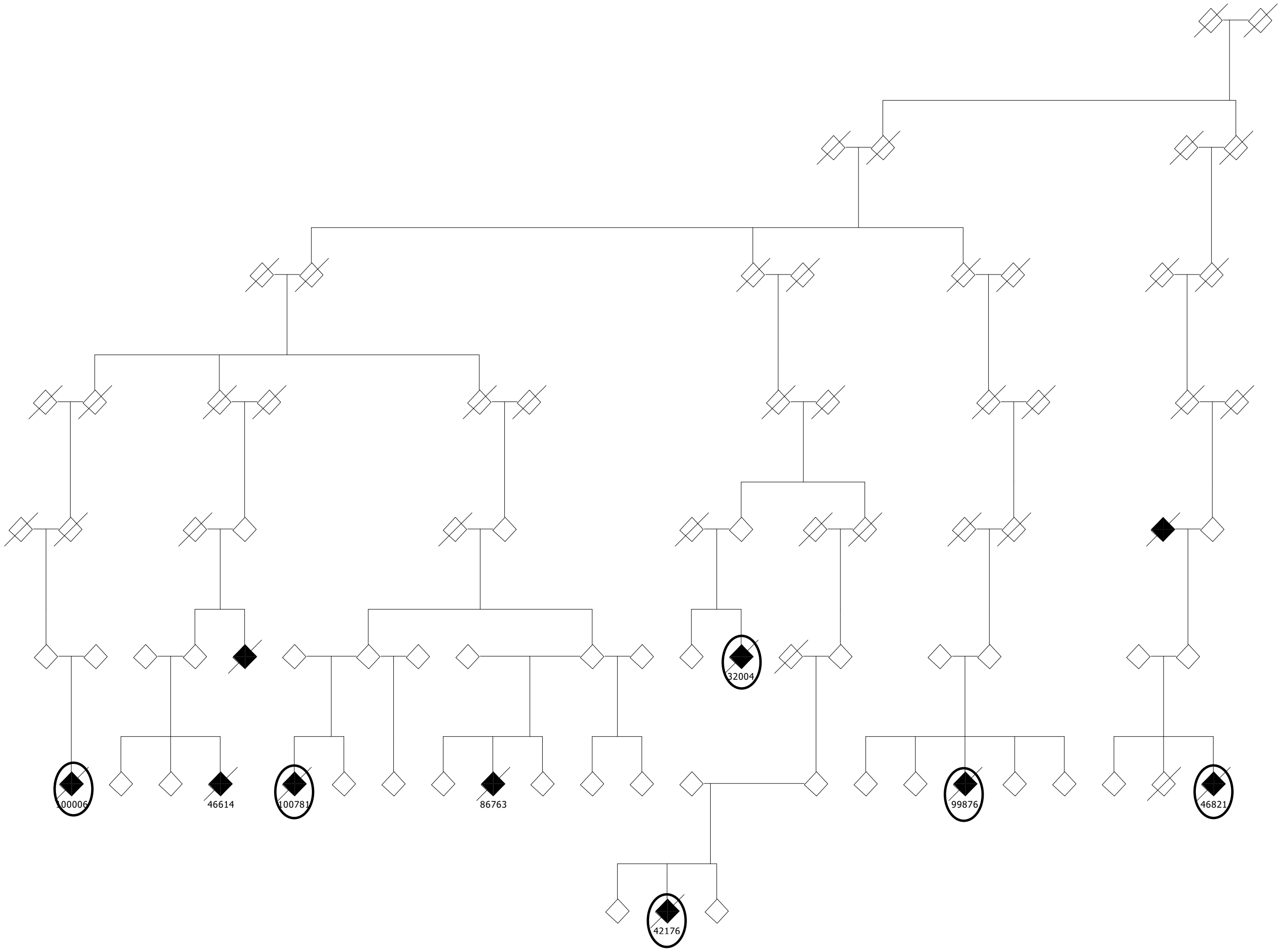

Family 8556

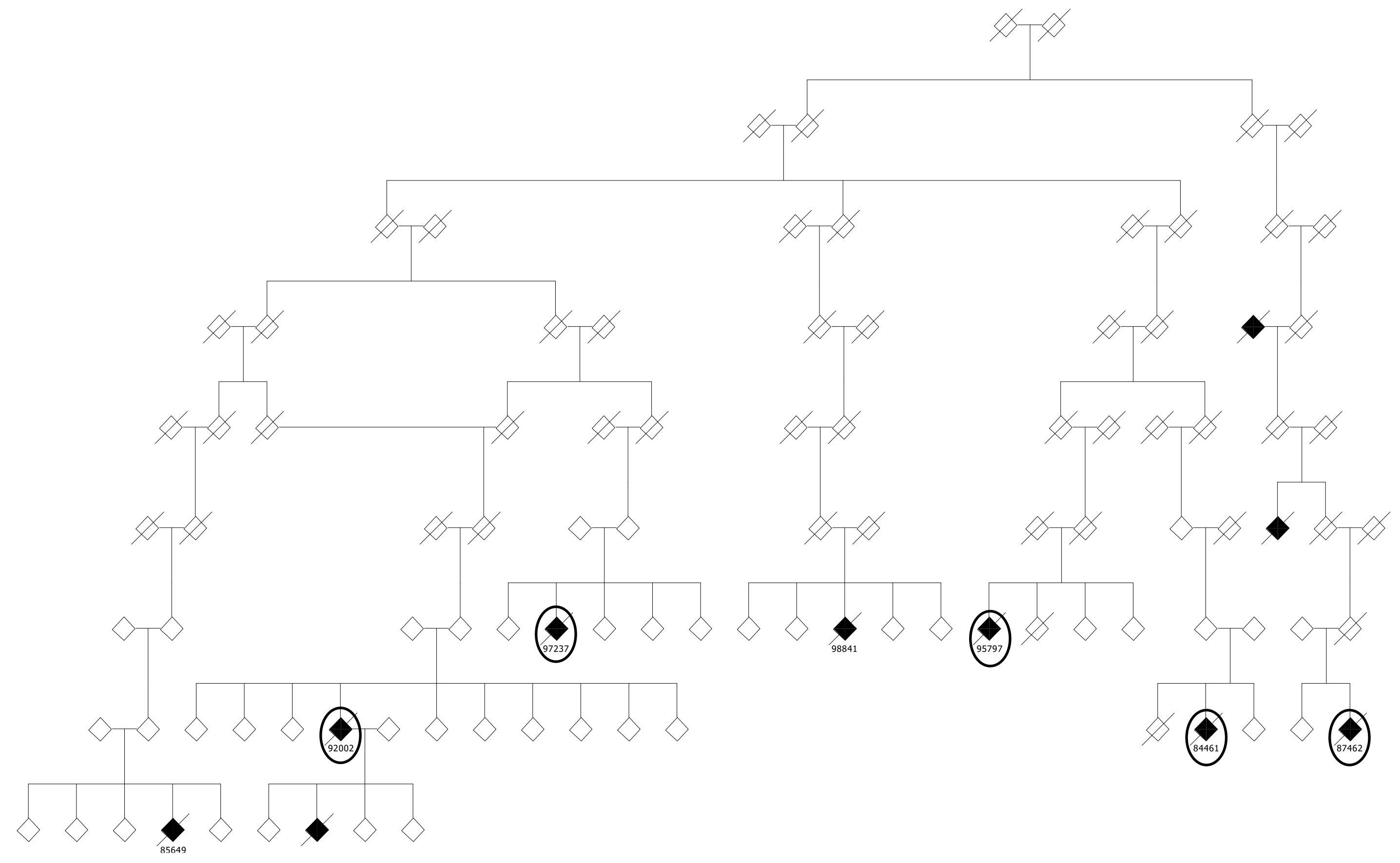

ii. All three analyzed cases in Family 791533 share a region on chromosome 1q31.2-q31.1 with all seven analyzed cases in Family 540775.

FAMILY 791533

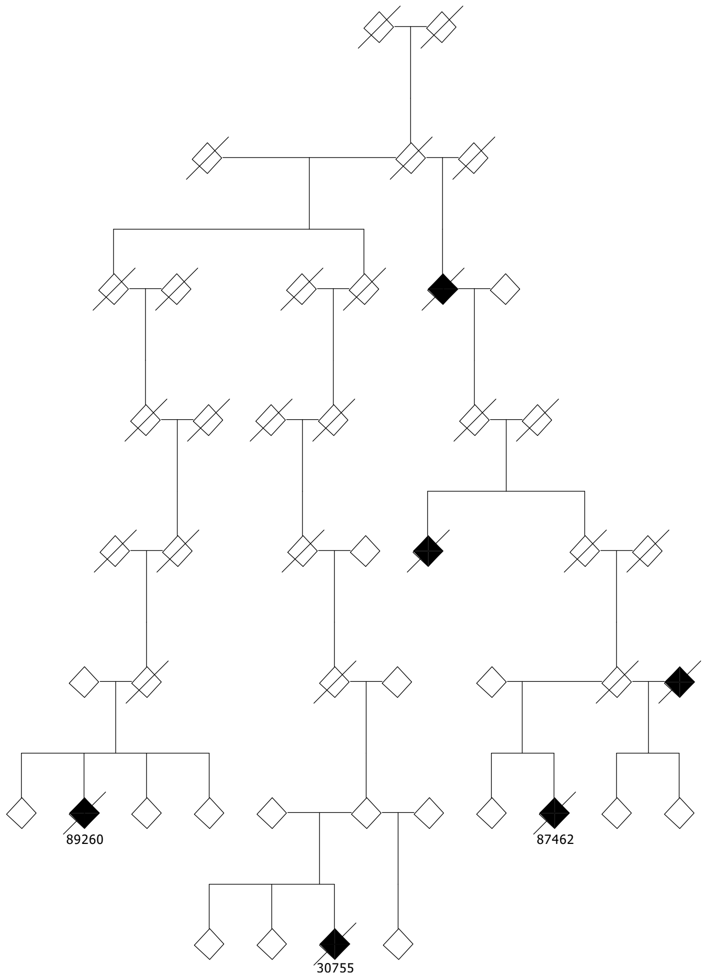

FAMILY 540775

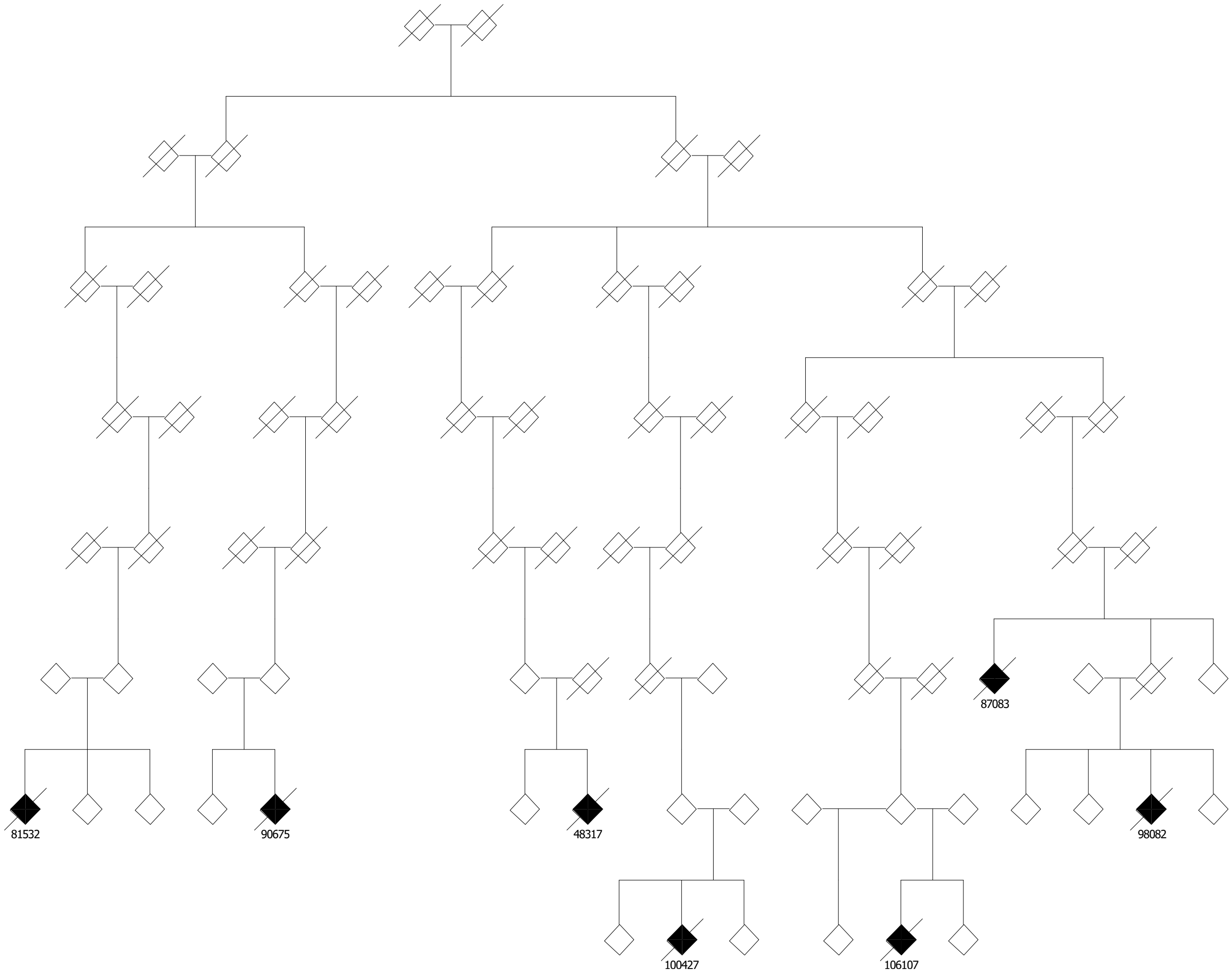

iii. The six analyzed cases in Family 11593 share a region on chromosome 2q32.2 – q32.3 with five of the cases in Family 176860 (the sharing cases are circled).

FAMILY 11593

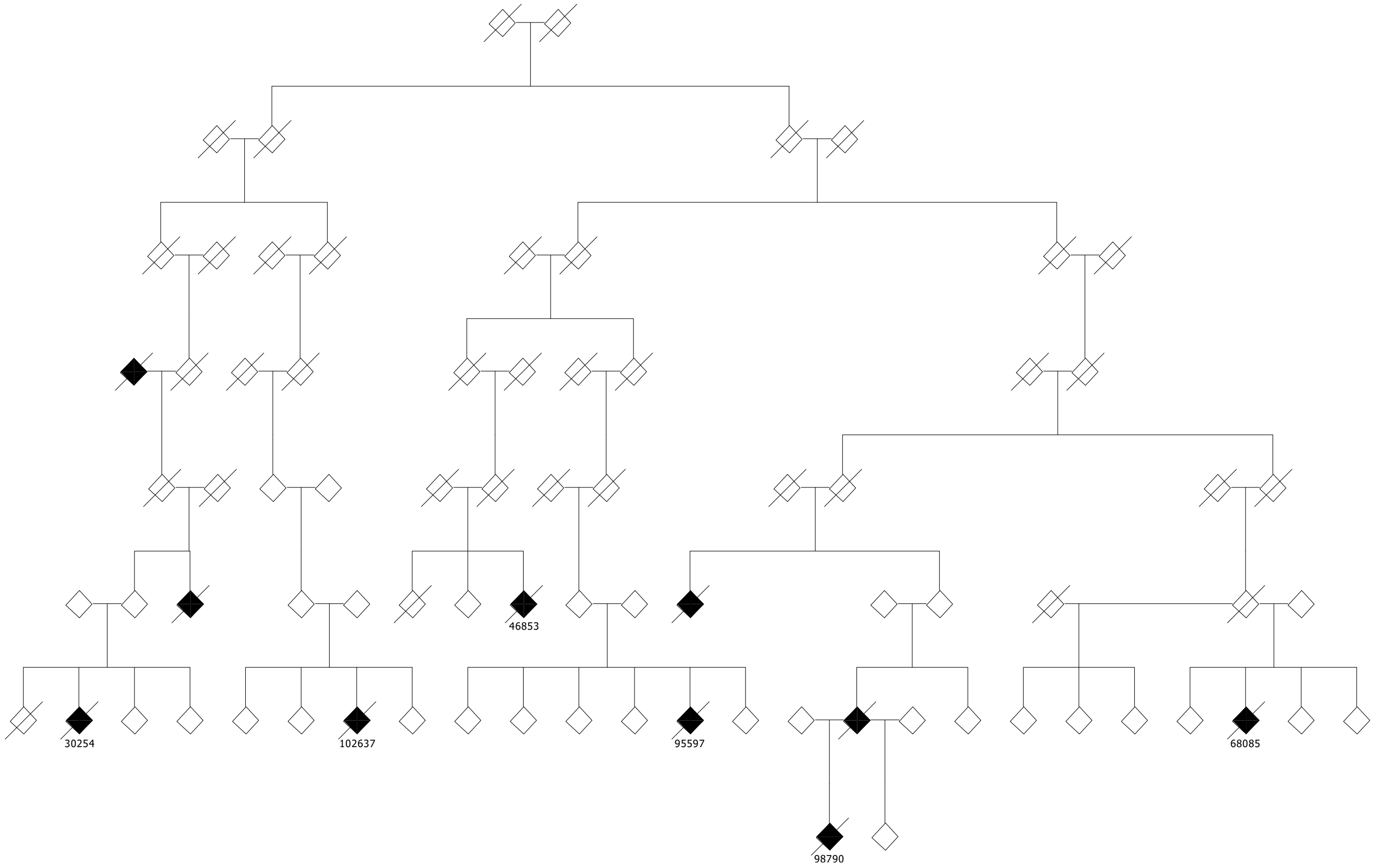

FAMILY 176860

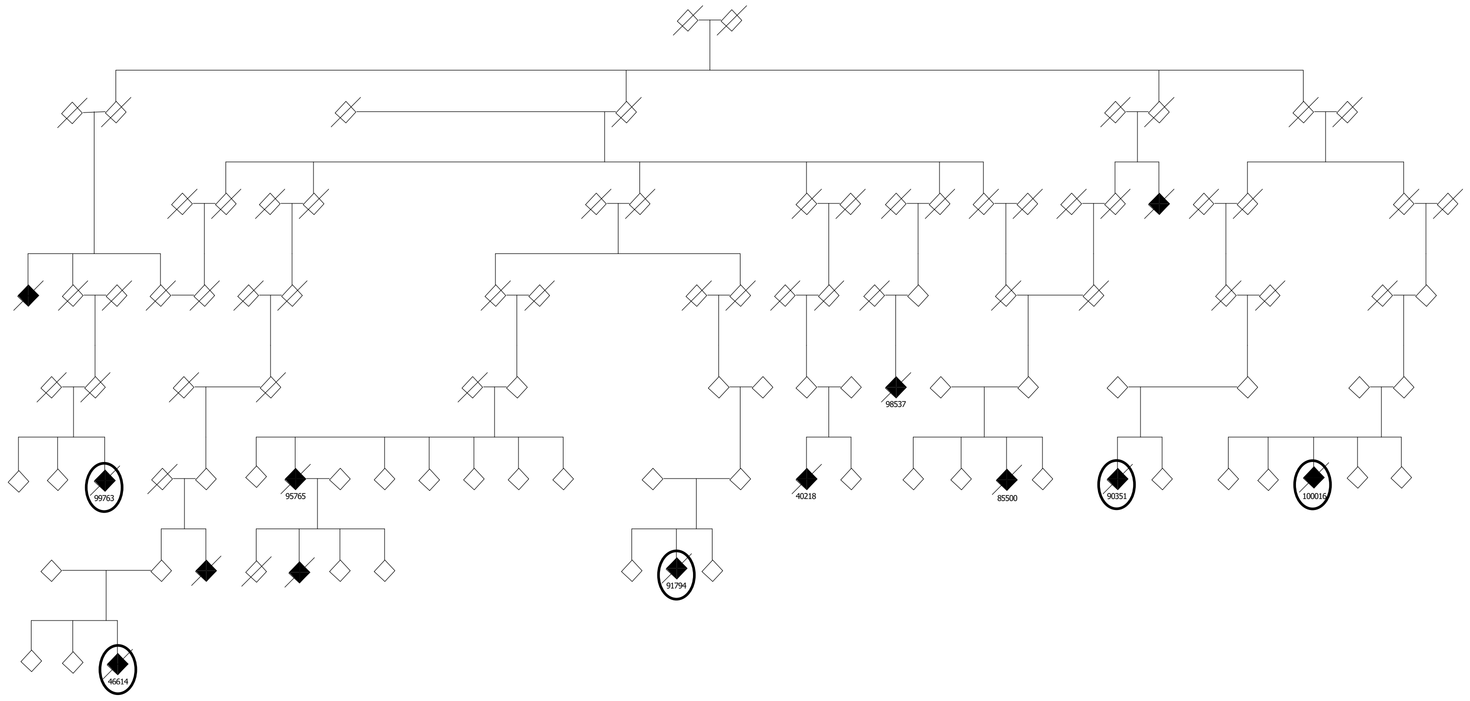

iv. Five cases in family 11593 share a region on 3q26.33 with four cases in family 129334 (the sharing cases are circled).

FAMILY 11593

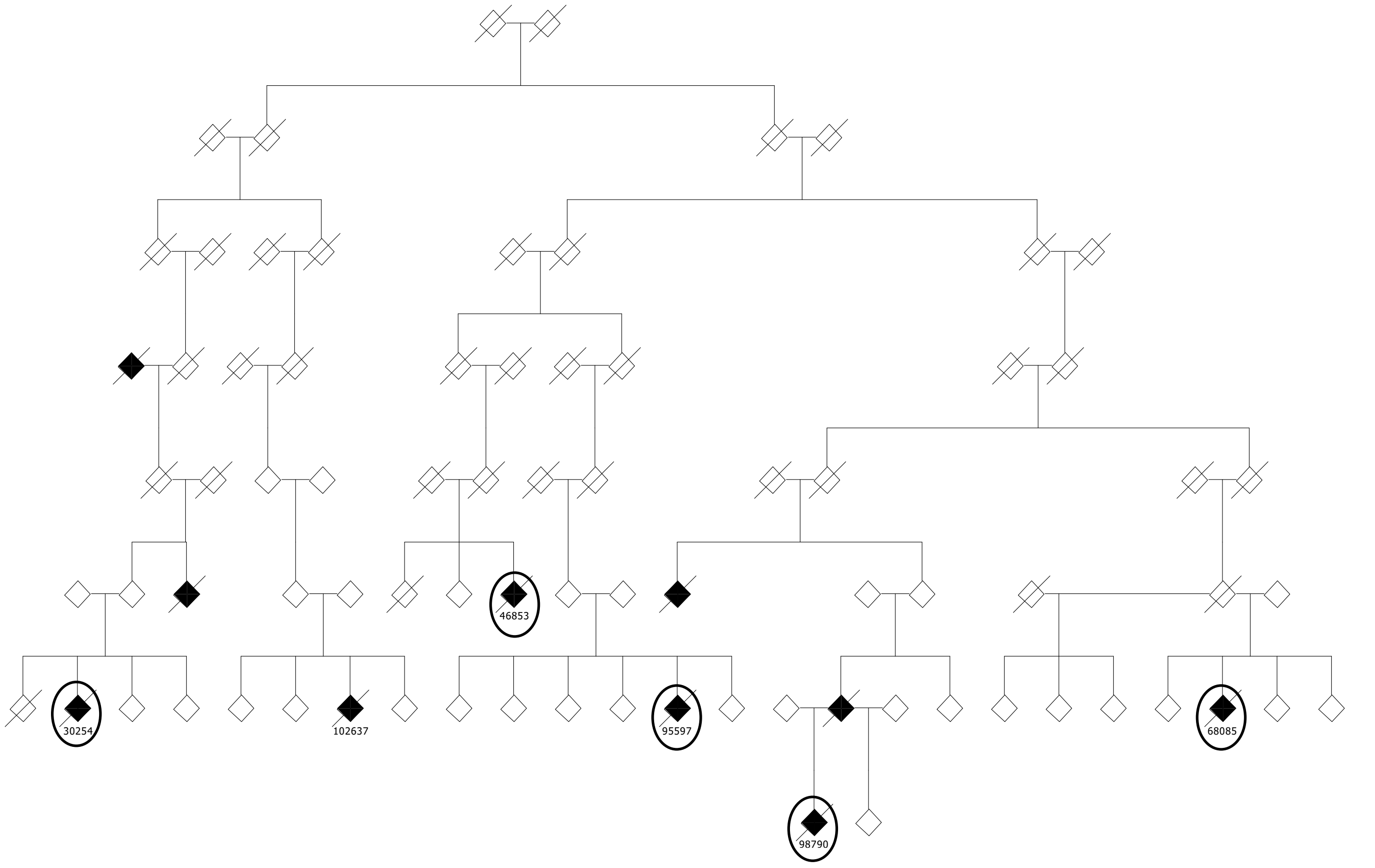

FAMILY 129334

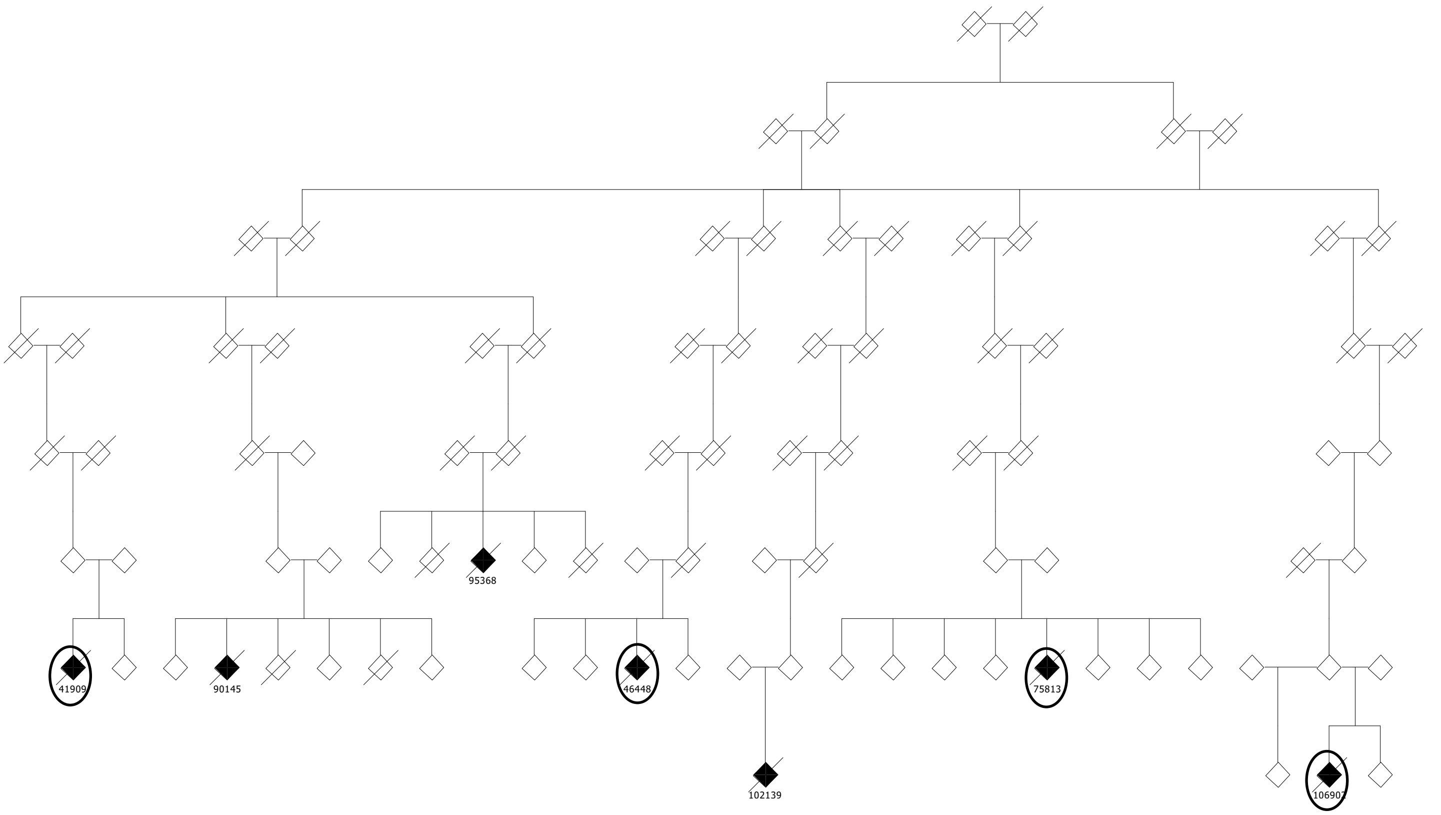

v. Four cases in family 8556 share a region on chromosome 4q35.1-35.2 with five cases in family 66494 (the sharing cases are circled).

FAMILY 8556

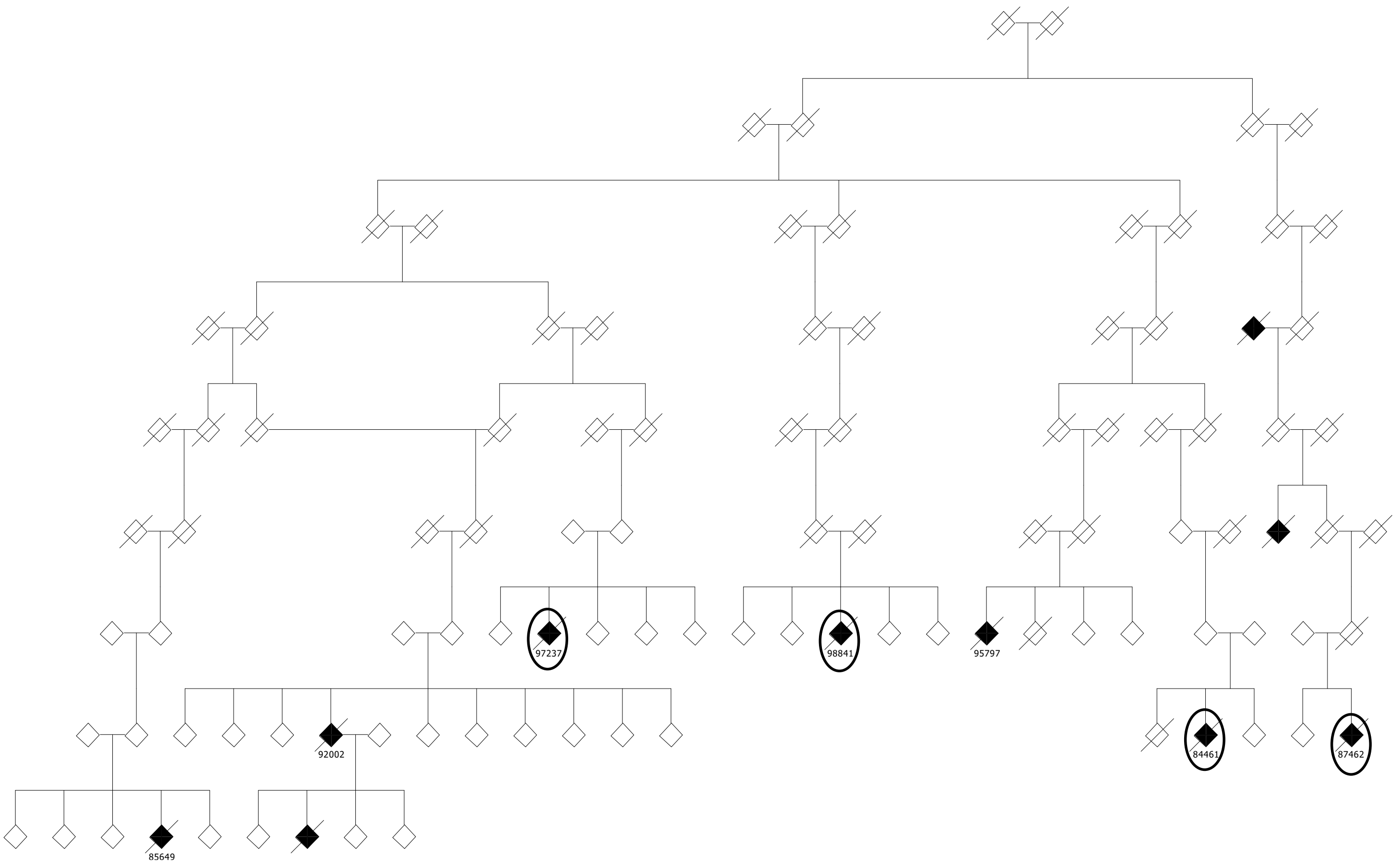

FAMILY 66494

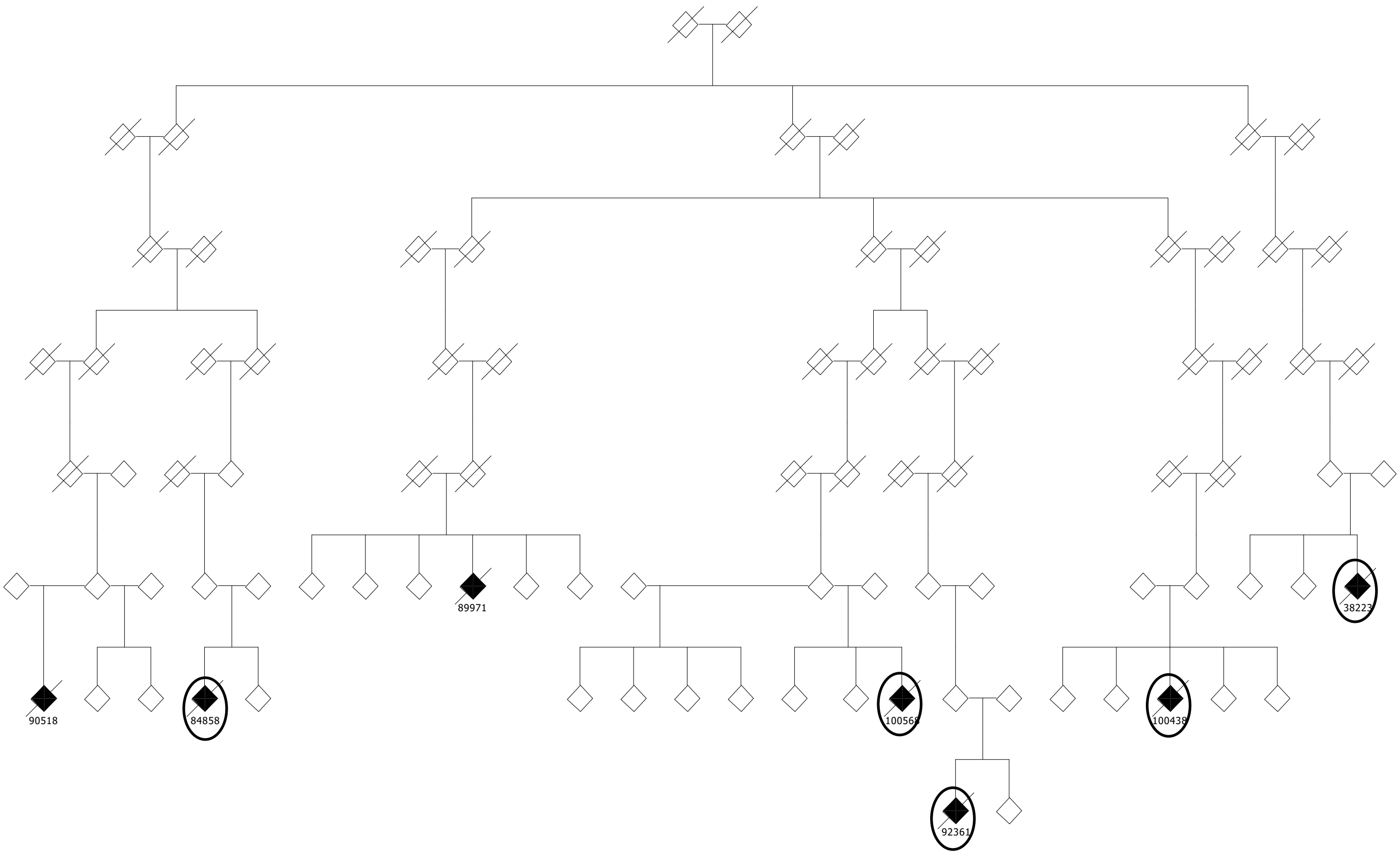

All three analyzed cases in Family 957634 share a region on chromosome 7q36.1 with four cases in Family 595955 (sharing cases are circled).

FAMILY 957634

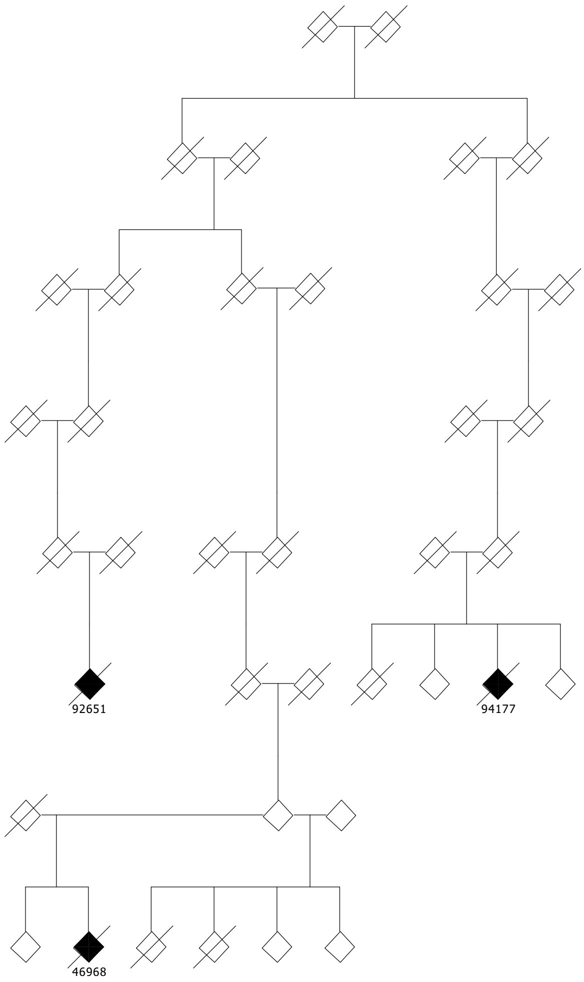

FAMILY 595955

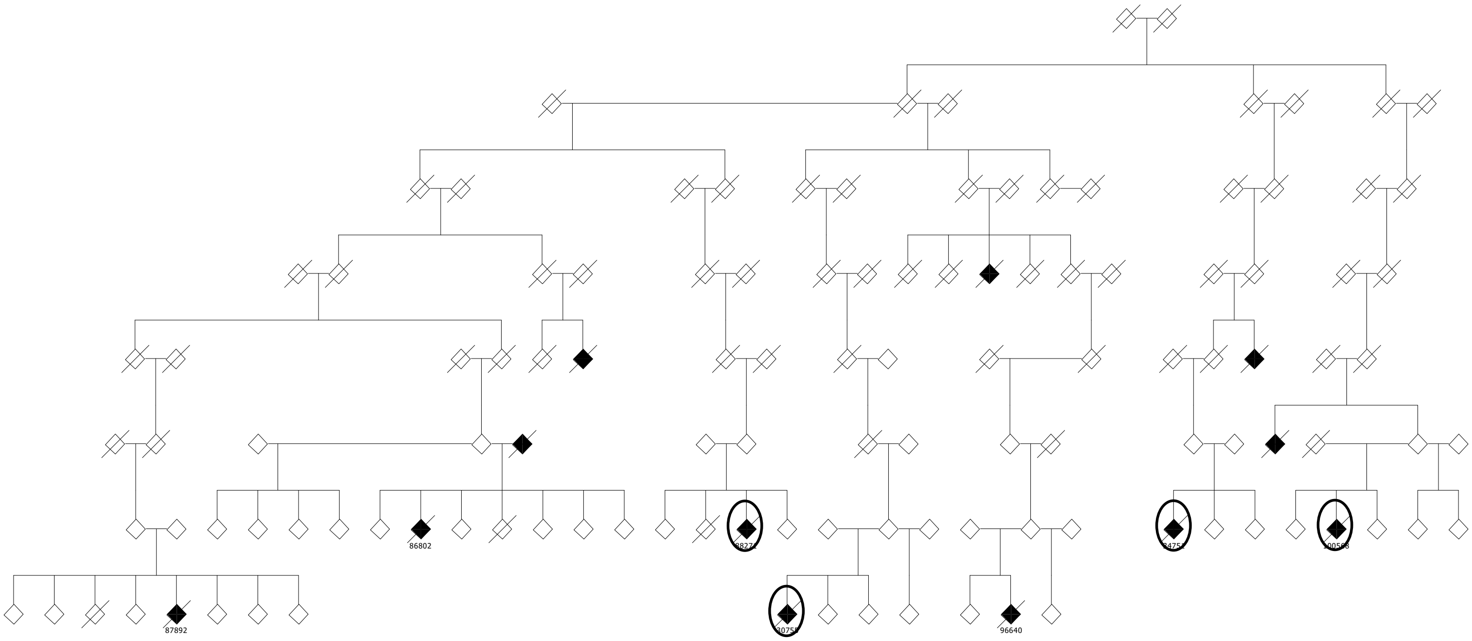

vi. Four cases in family 587072 share a region on chromosome 8p23.1 with five cases in family 595955 (sharing cases in the two families are circled).

FAMILY 587072

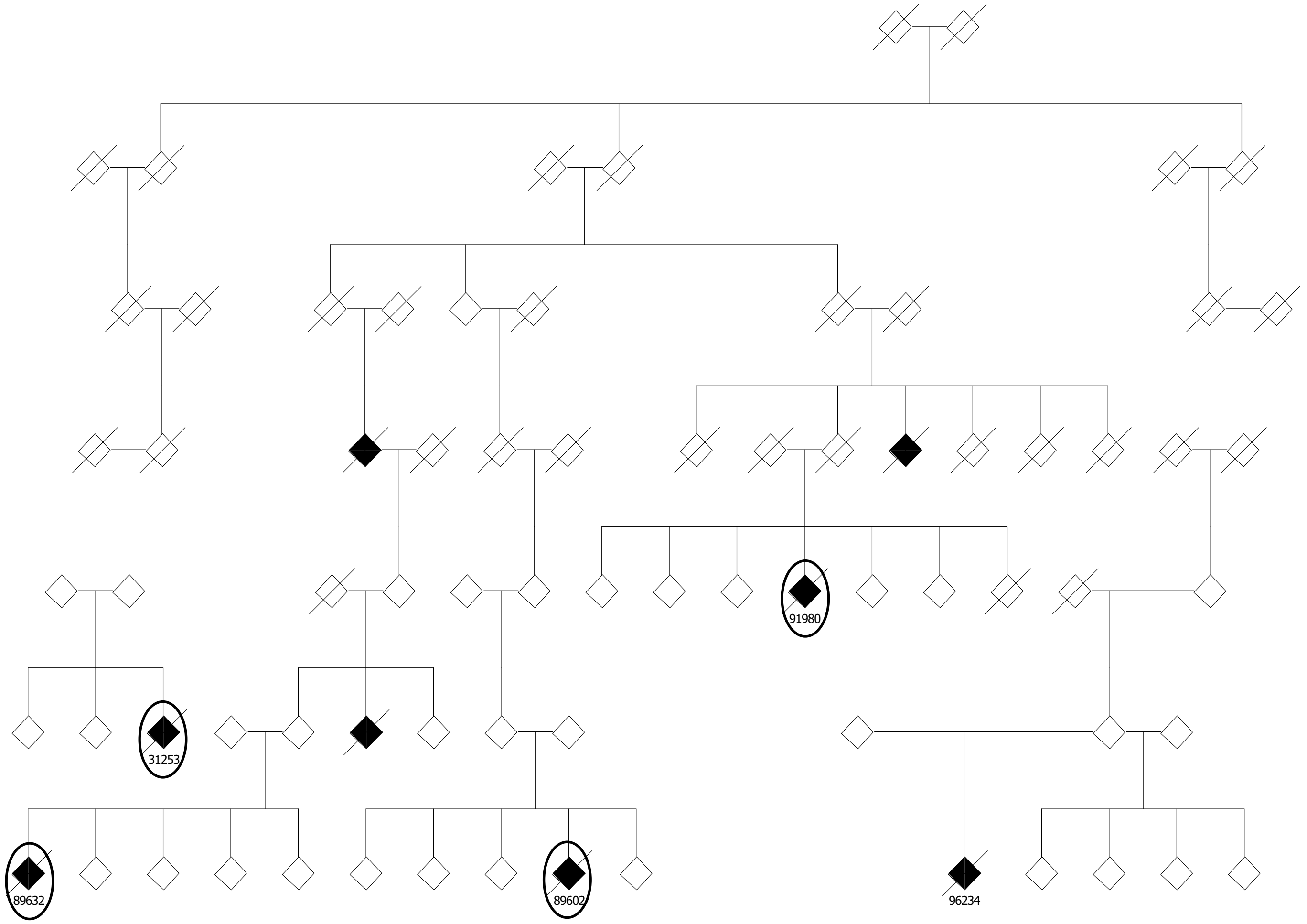

FAMILY 595955

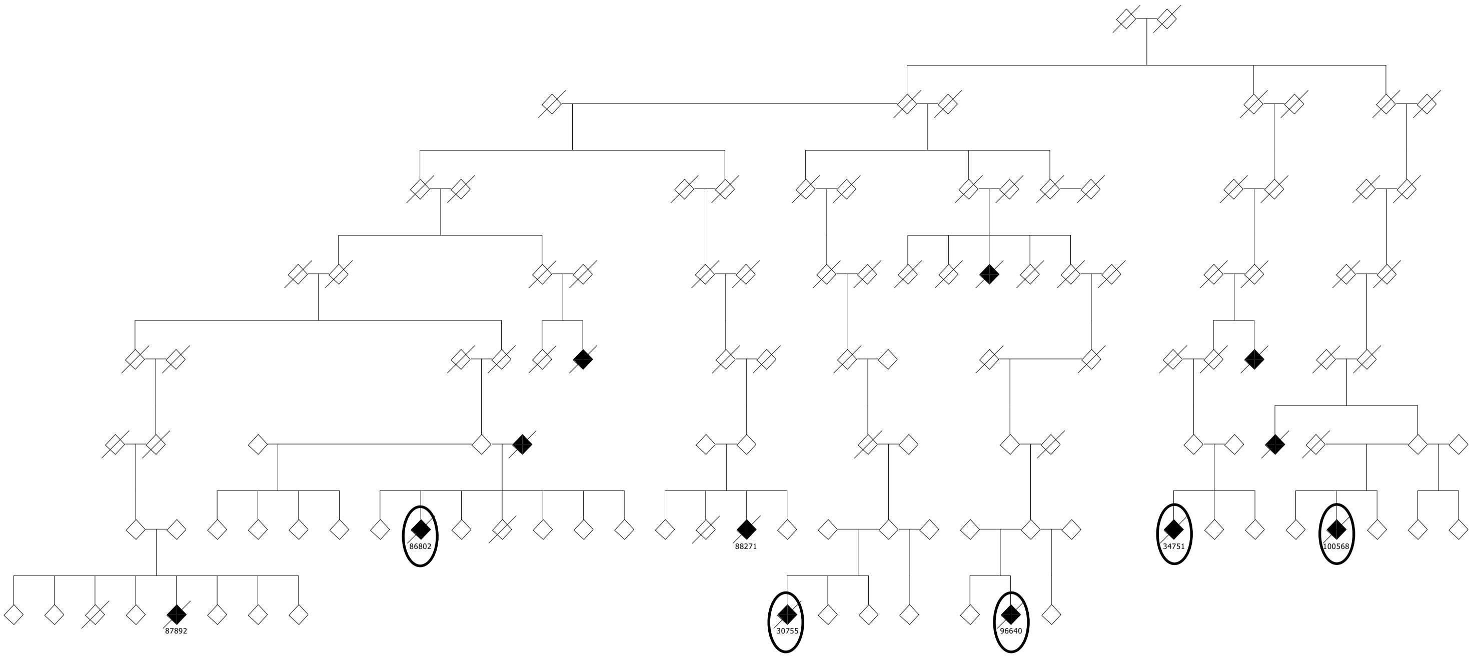

vii. Five cases in family 8556 share a region on chromosome 10p12.33 with four cases in family 11593 (sharing cases in the two families are circled).

FAMILY 8556

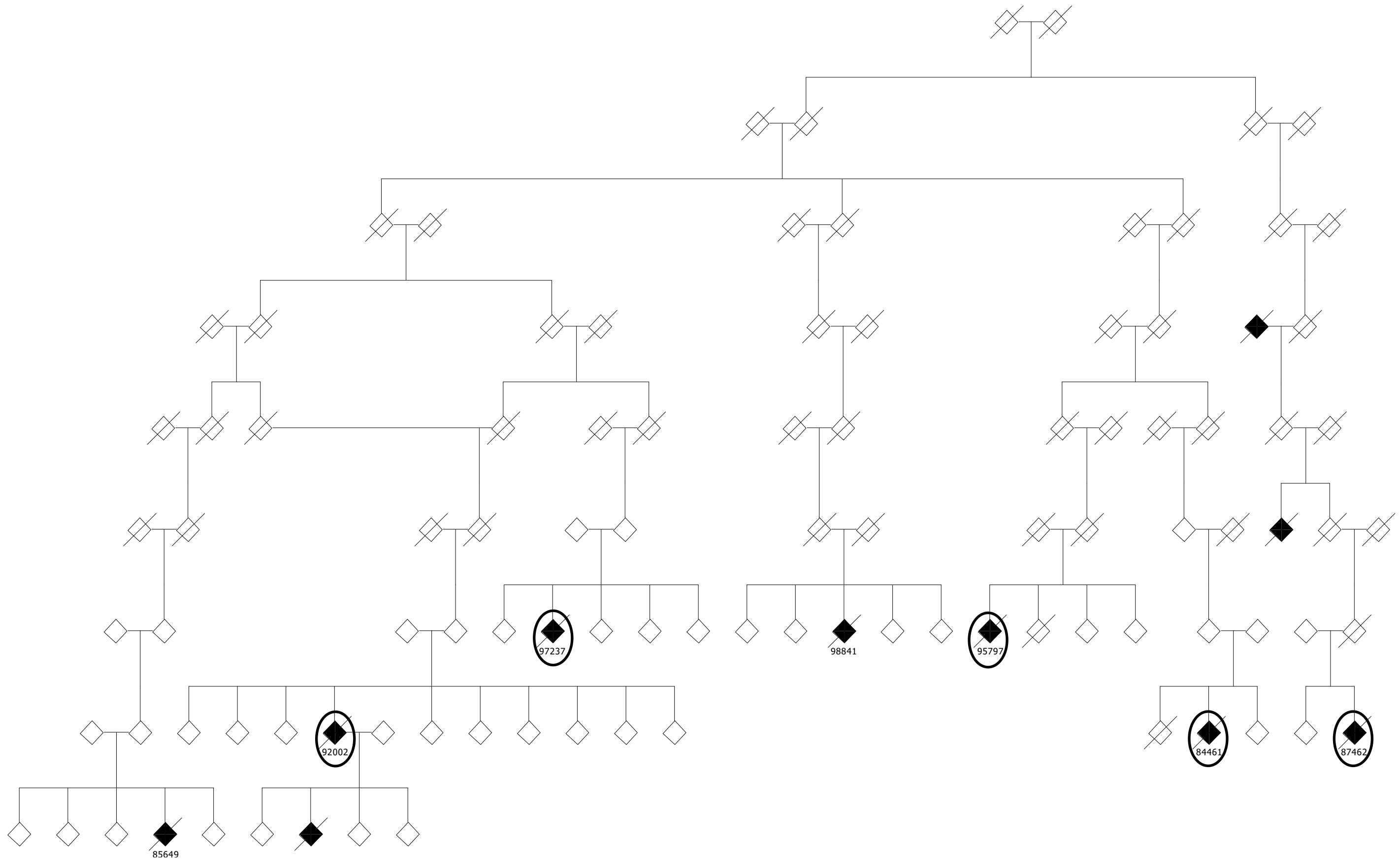

FAMILY 11593

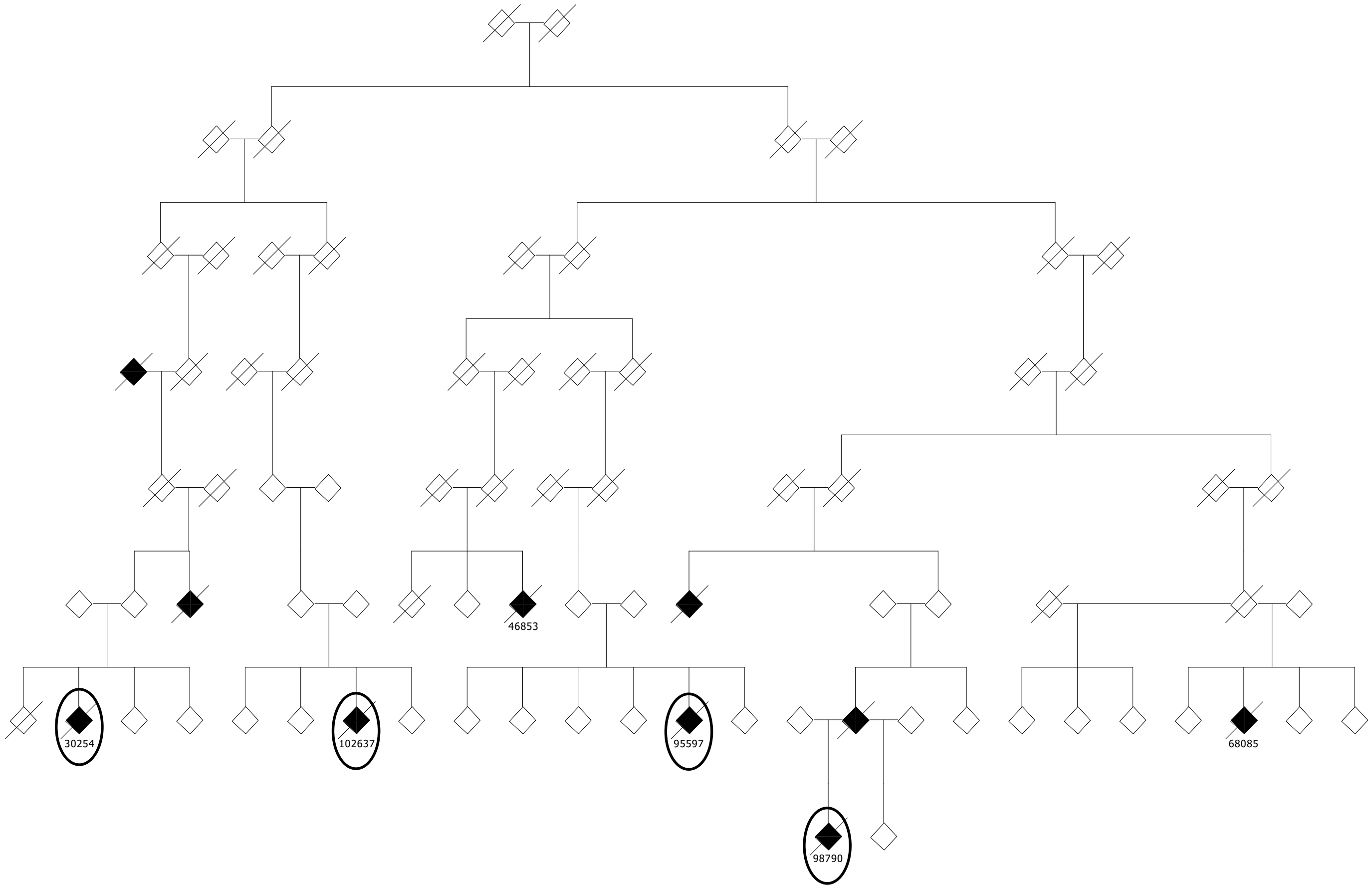

viii. Seven cases in Family 27251 share a region on chromosome 10q21.3 with five cases in Family 233769 (sharing cases in the two families are circled).

FAMILY 27251

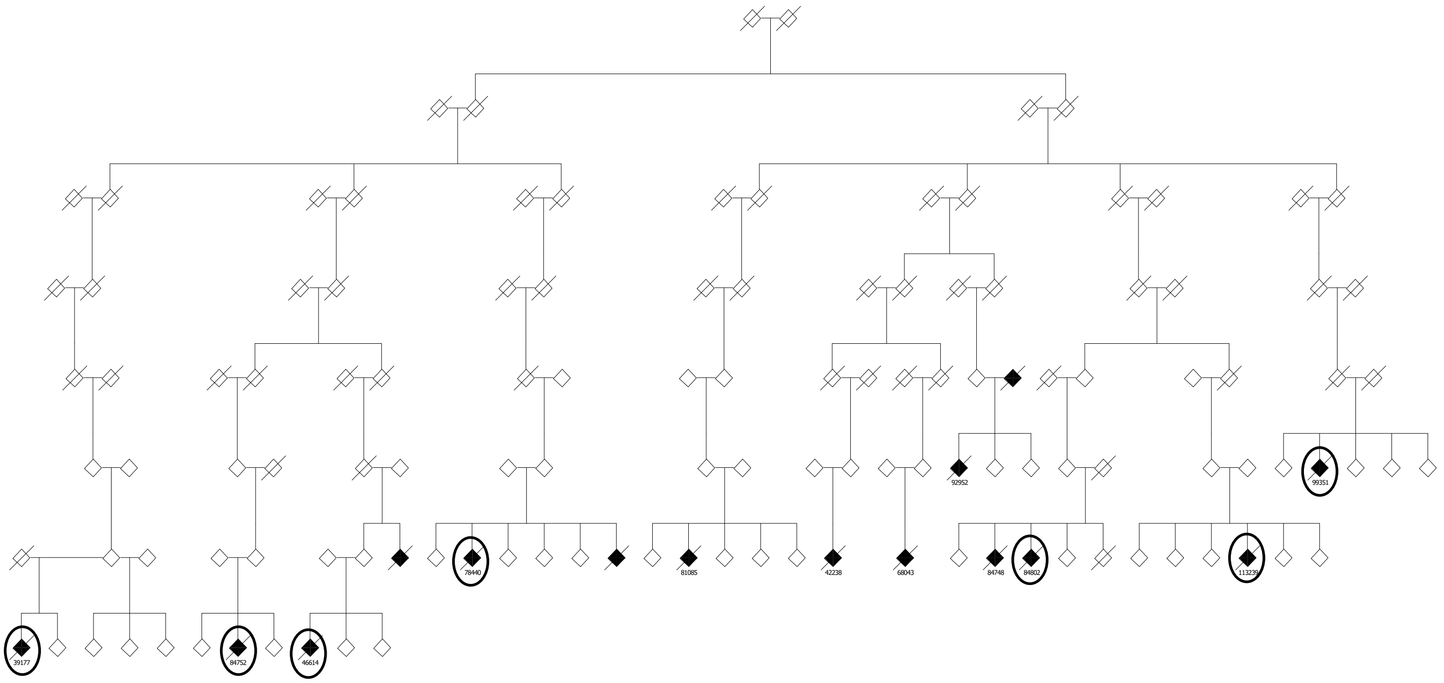

FAMILY 233769

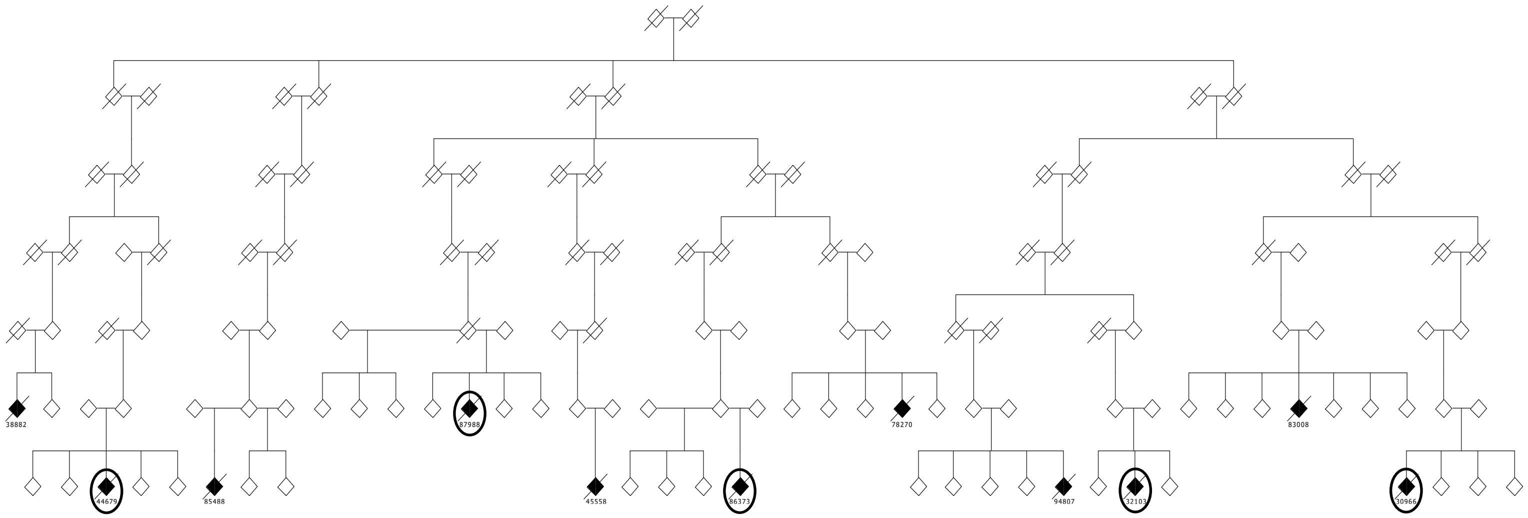

ix. Five cases in Family 66494 share a region on chromosome 12q.12 with five cases in Family 209487 (sharing cases in the two families are circled).

FAMILY 66494

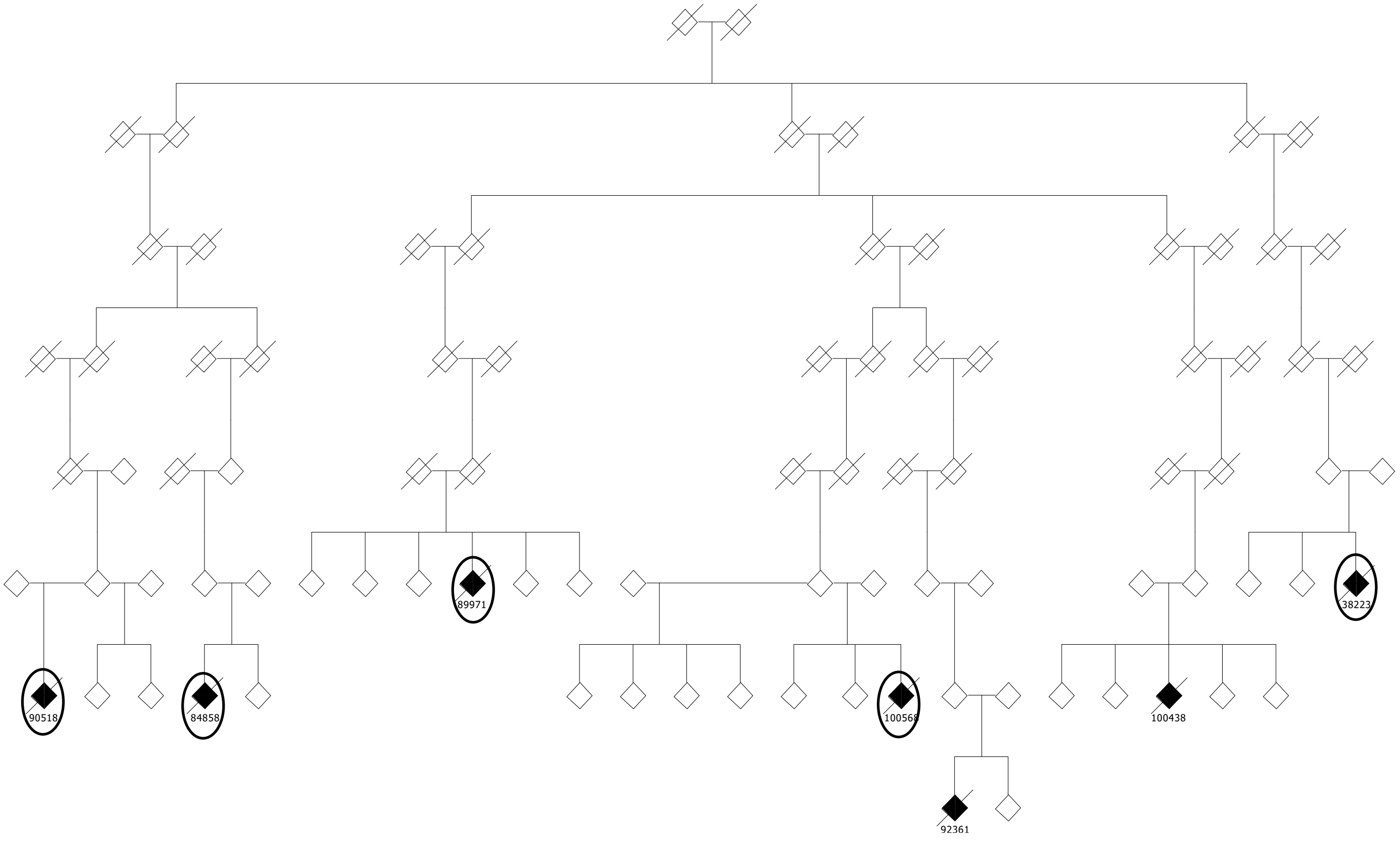

FAMILY 209487

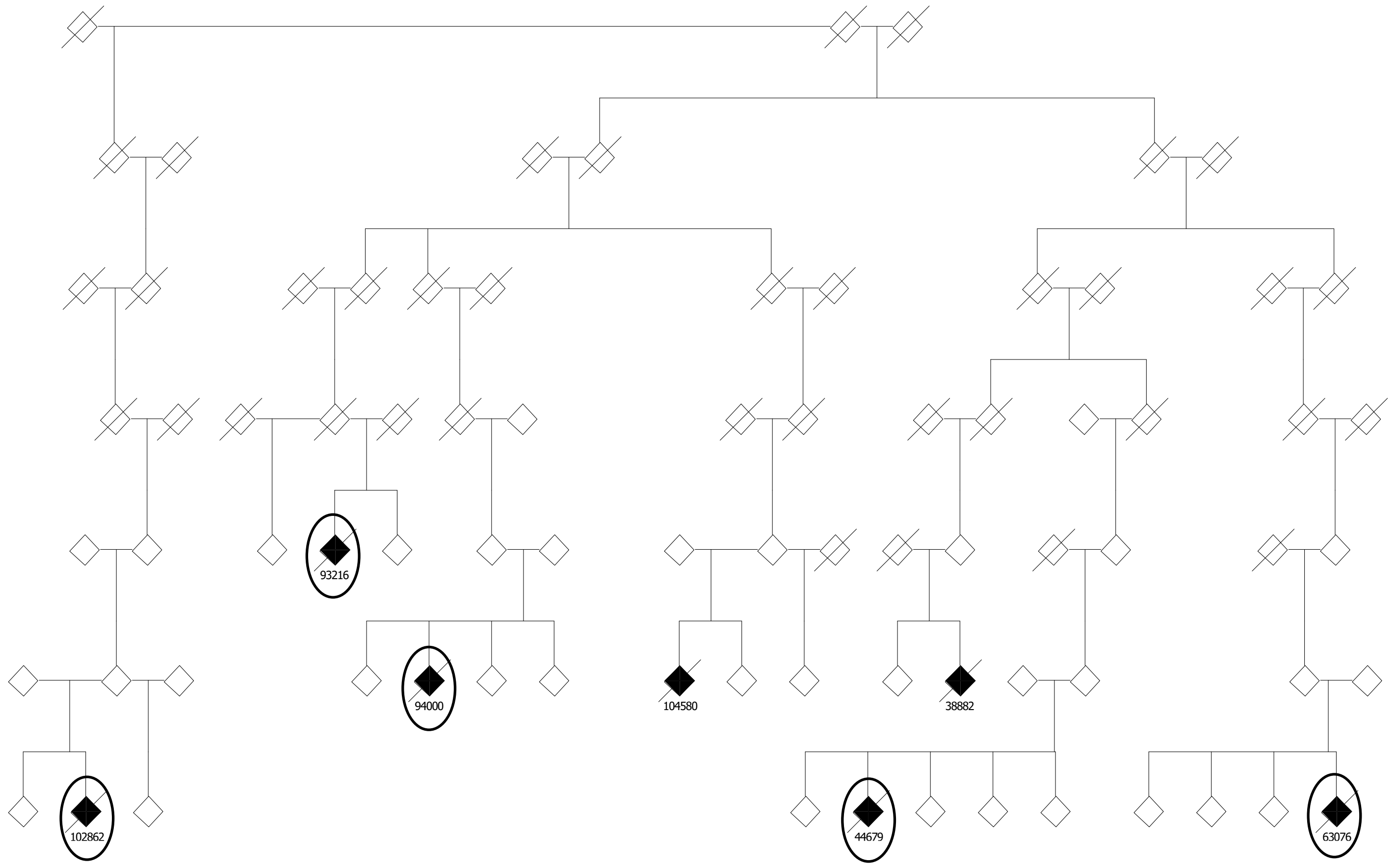

x. All four analyzed cases in Family 41469 share a region on chromosome 13q14.2 with five cases in Family 27251 (circled).

FAMILY 41469

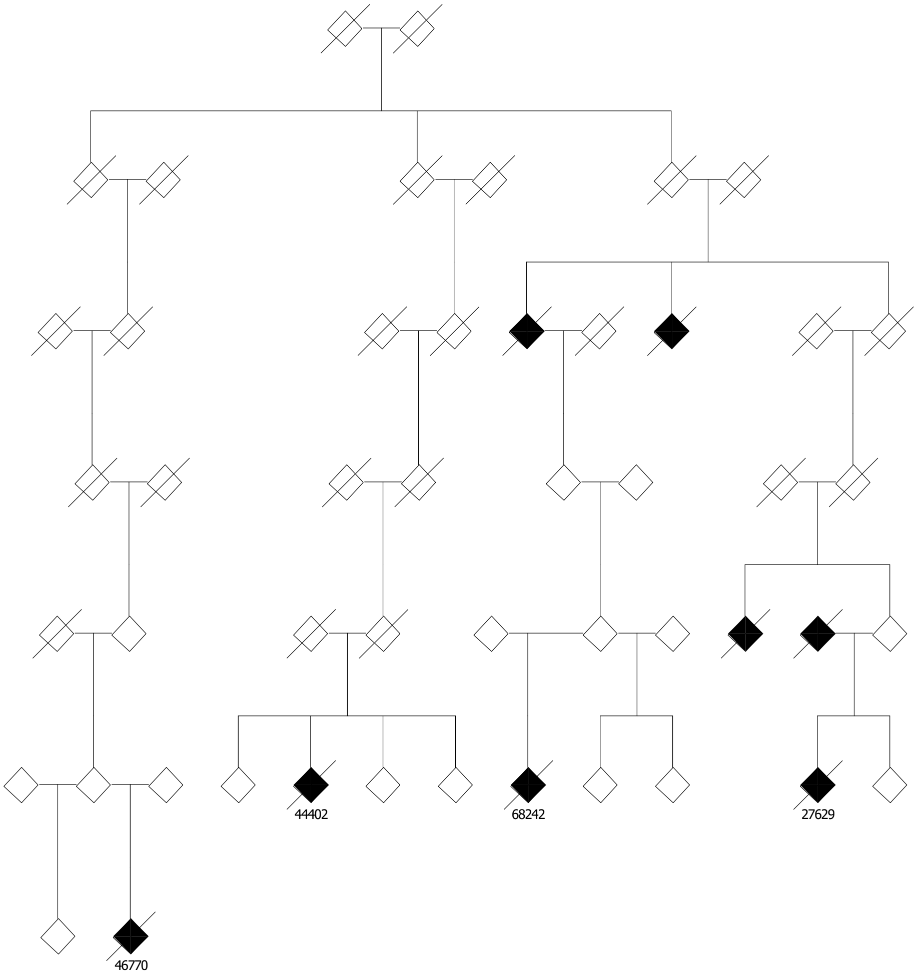

FAMILY 27251

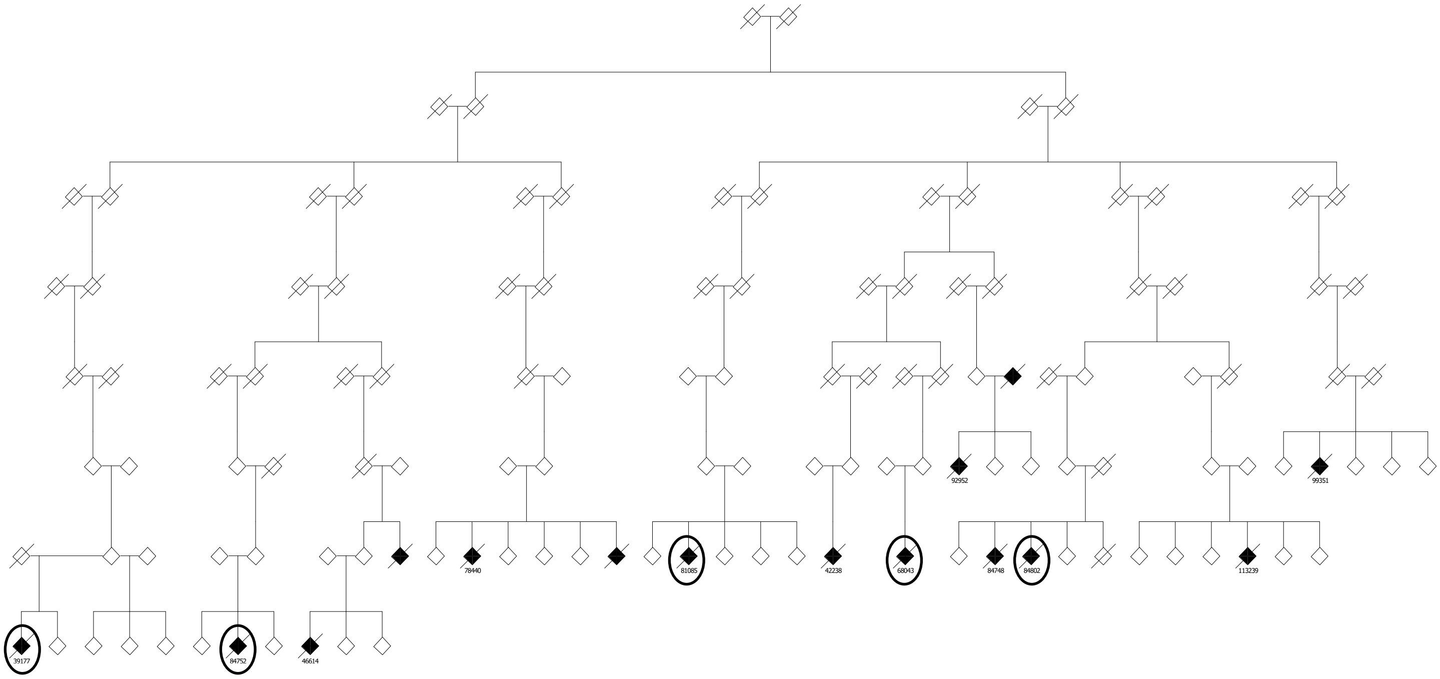

xi. Five cases in Family 590241 share a region on chromosome 14q23.1-q23.2 with eight cases in family 601627 (sharing cases circled).

Family 590241

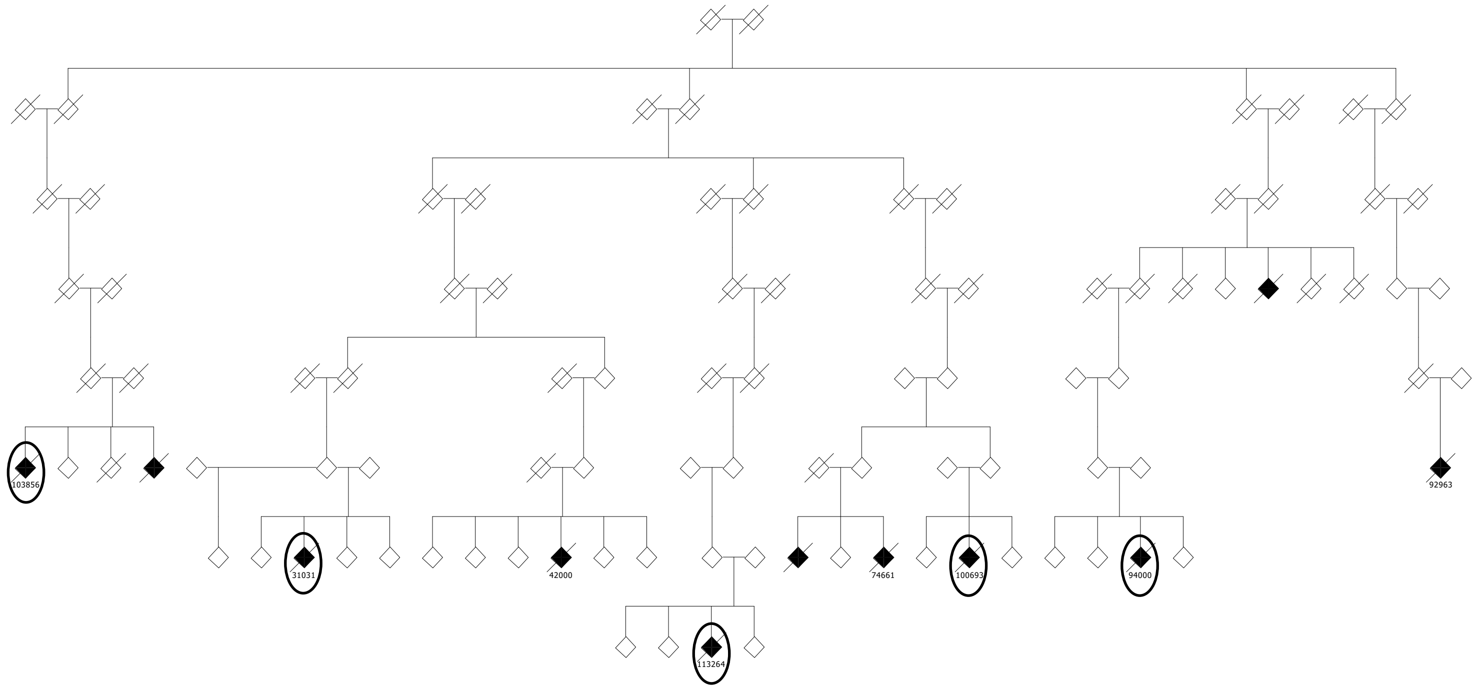

Family 601627

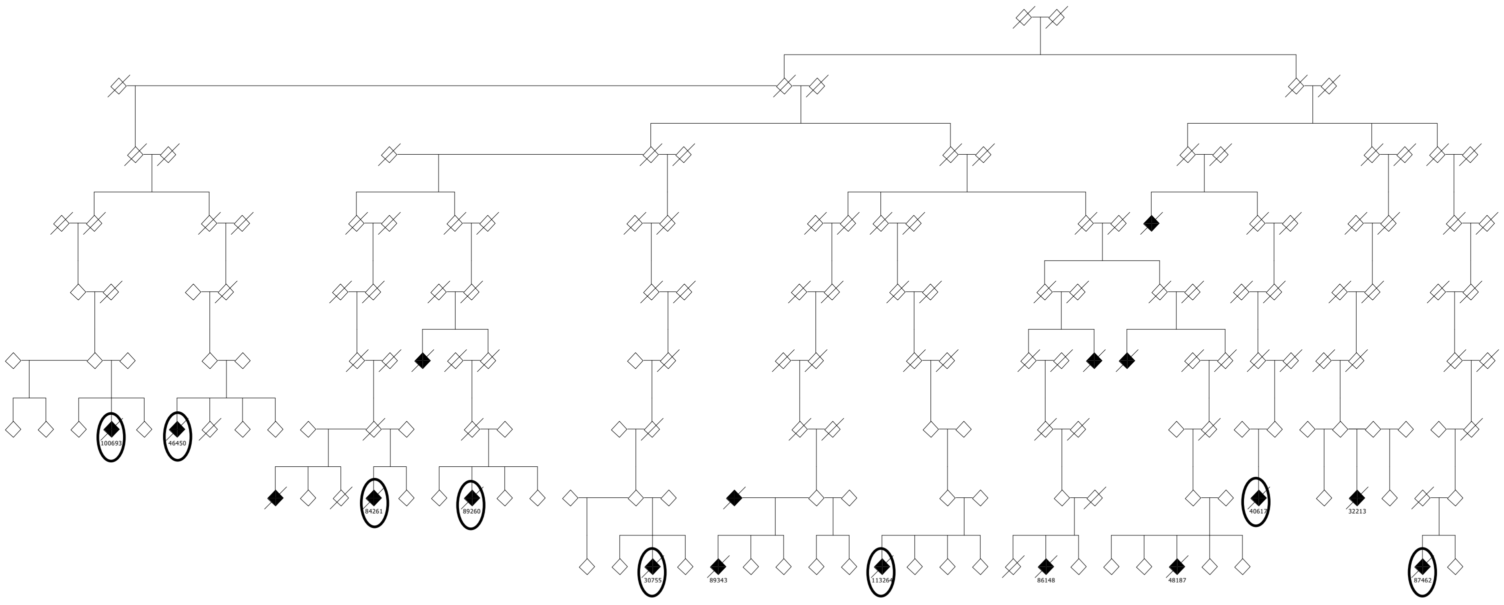

xii. Eight cases in Family 27251 share a region on chromosome 18q11.2 with six cases in Family 233769 (sharing cases are circled).

FAMILY 27251

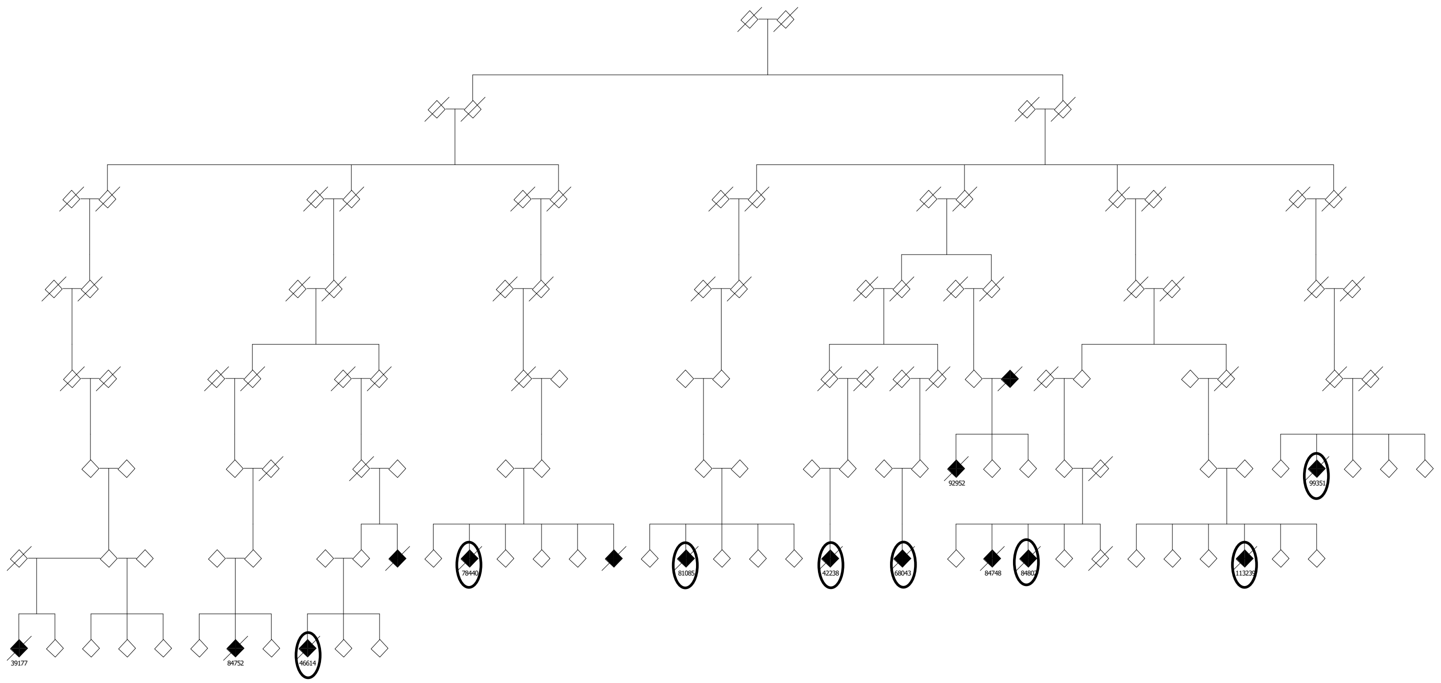

FAMILY 233769

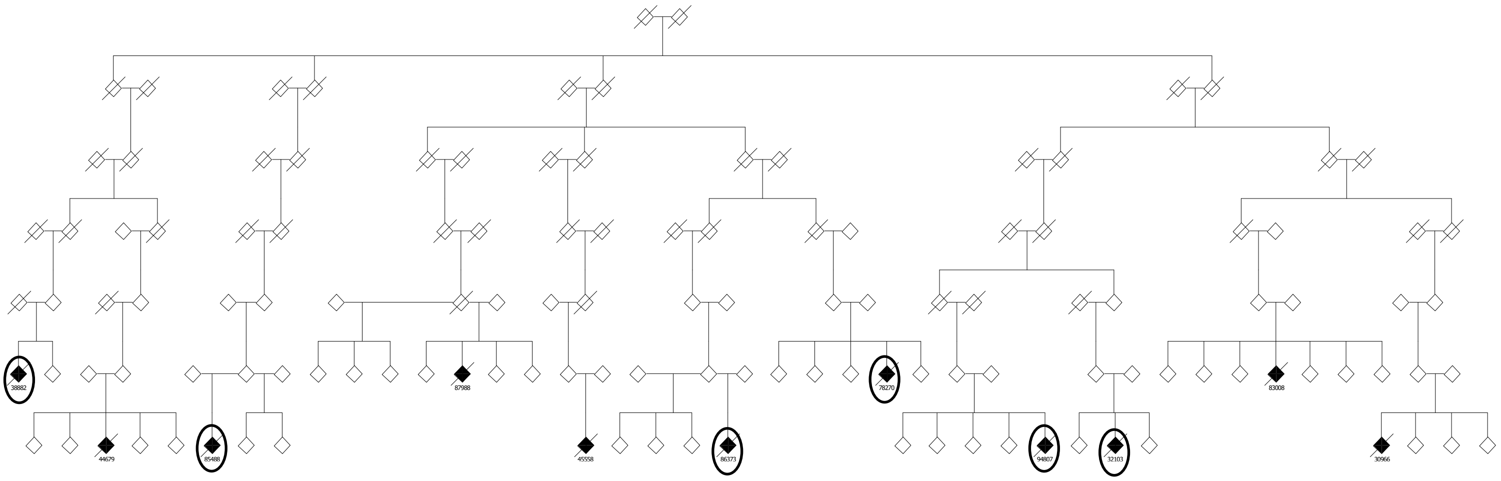

xiii. Eight cases in Family 27251 share a region on chromosome 19q13.12 with three cases in family 622459 (sharing cases are circled).

FAMILY 27251

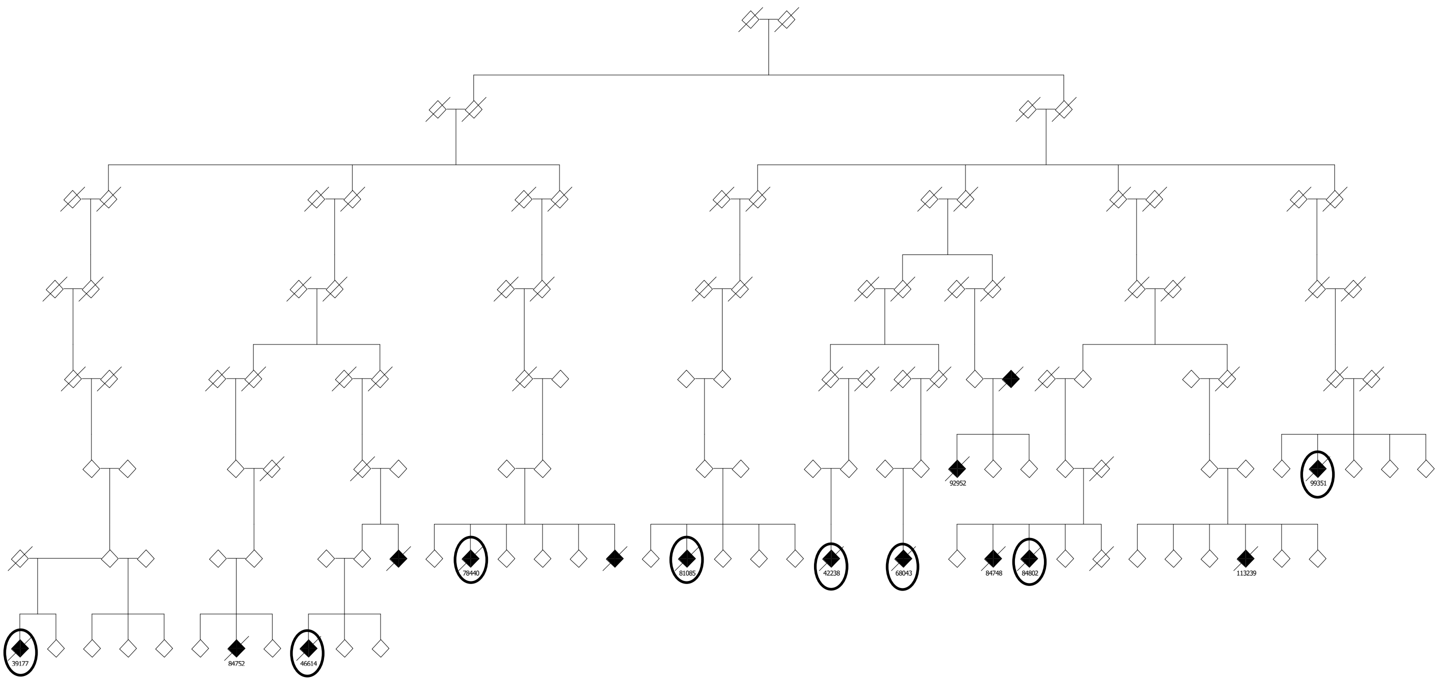

FAMILY 622459

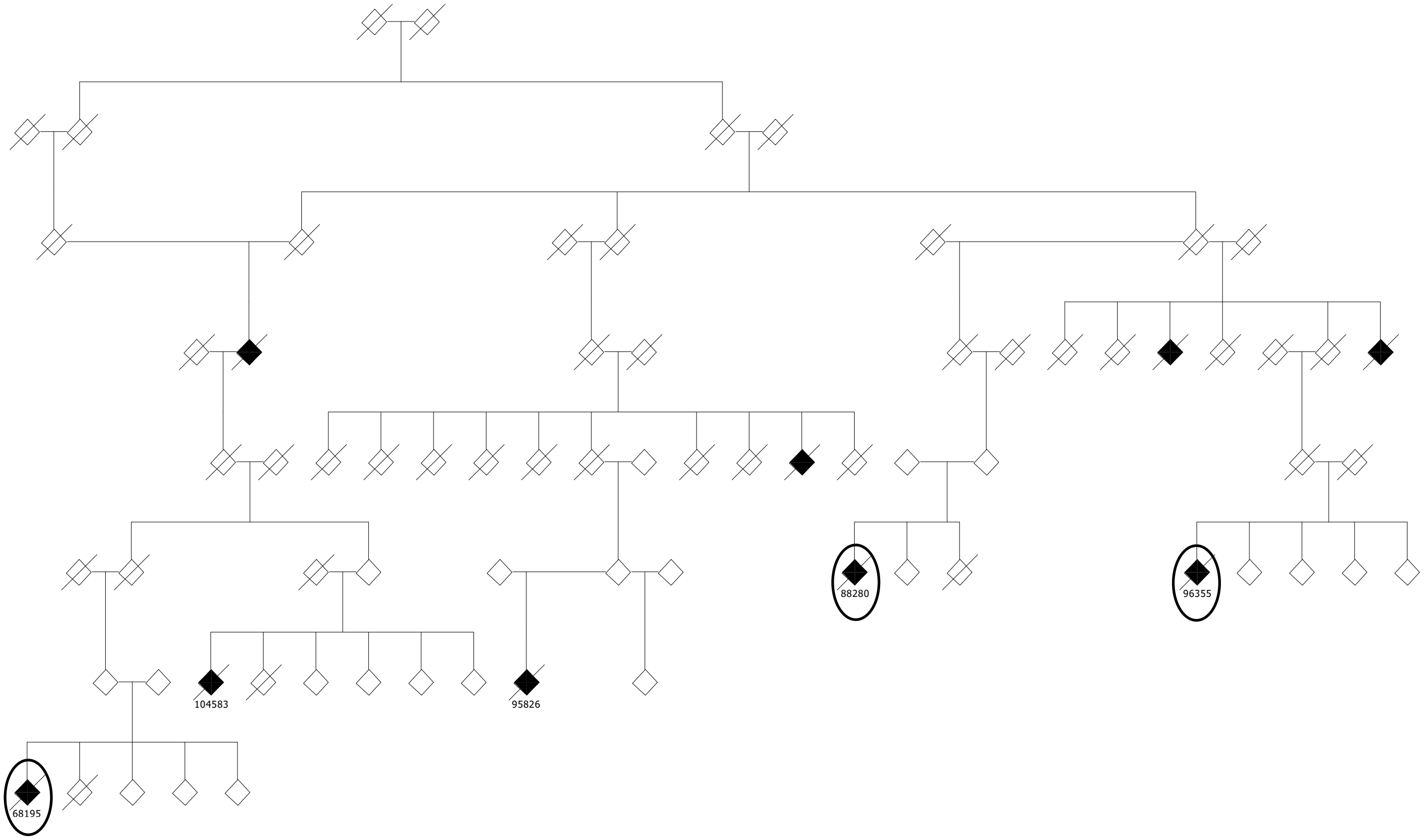

xiv. Seven cases in Family 176860 (circled) share a region on chromosome 5q23.3-q31.1 with seven cases in Family 603481 (circled) and seven cases in Family 553615 (circled). NOTE: To assess overlap significance, case 95765 which occurs in Families 175860 and 553615 is only analyzed in Family 175860. Similarly, case 112304, which occurs in Families 603481 and 553615 is only analyzed in Family 603481. Studies to determine potential loops, inbreeding, or relatedness above the known top generations of these families that may explain these two cases are underway.

FAMILY 176860

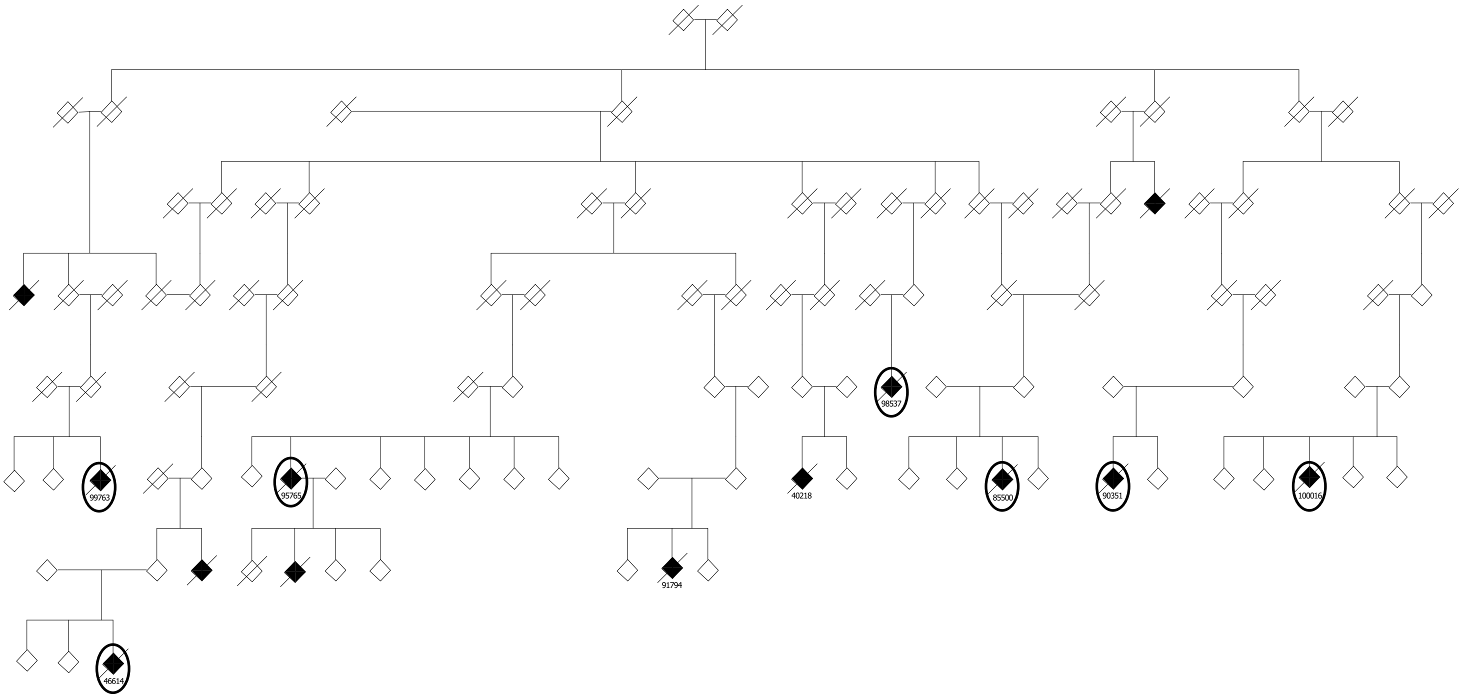

FAMILY 603481

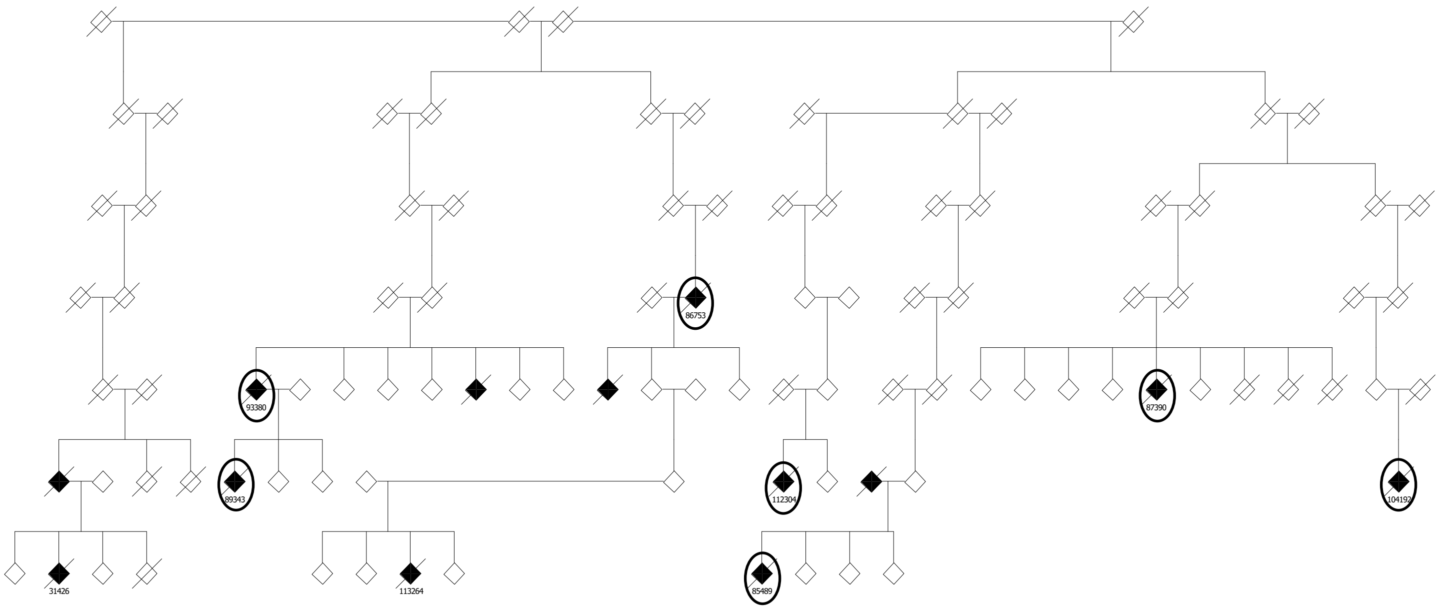

FAMILY 553615

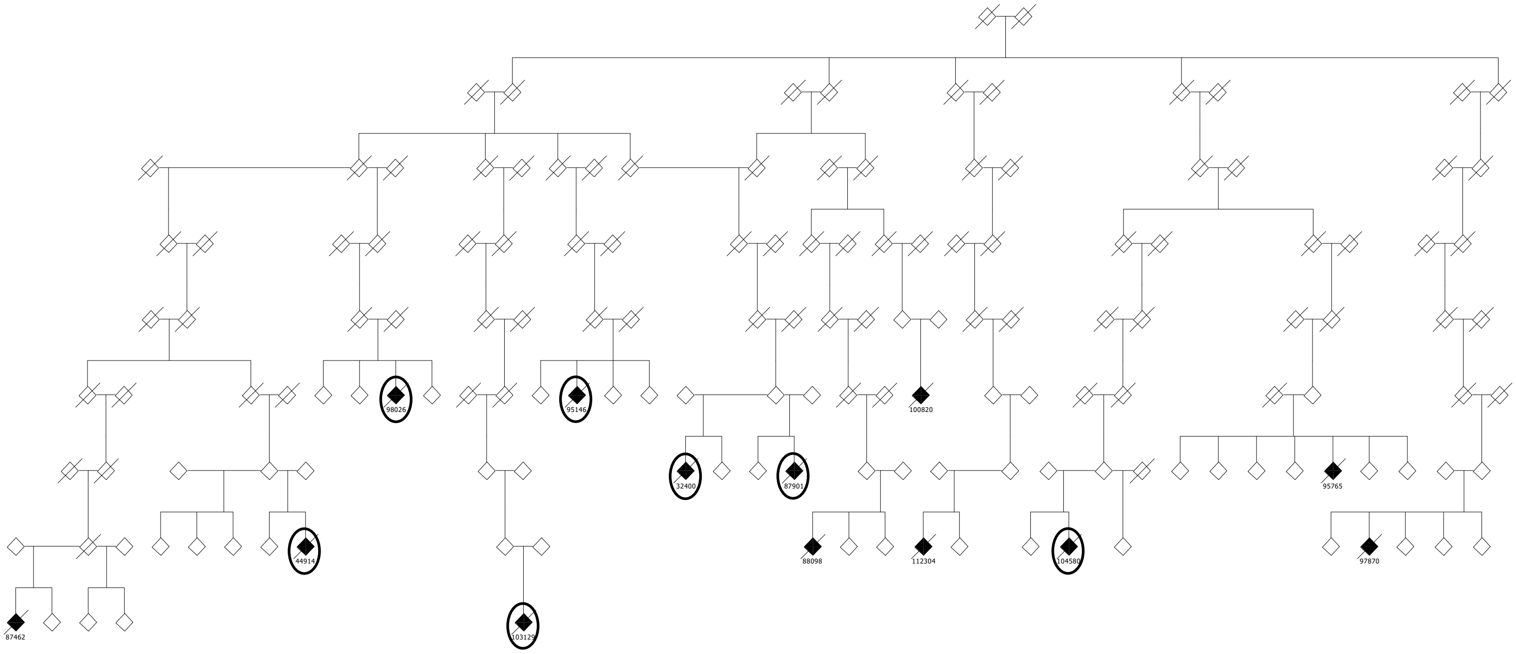

Supplement: Supplementary file 3 — Supplementary Materials [file 41380_2018_282_MOESM3_ESM.pdf]
